# Targeting Cystine Metabolism in the Lung Cancer Environment Enhances the Efficacy of Immune Checkpoint Inhibition

**DOI:** 10.1002/advs.202413084

**Published:** 2025-07-10

**Authors:** Yun Xu, Shumin Li, Jiaji Wu, Shumin Xu, Mo Shen, Chenyang Wang, Lundqvist Andreas, Jianghao Yu, Zhiyong Xu, Yueli Shi, Nana Liu, Yunke Yang, Jiangnan Zhao, Ying Yang, Pingli Wang, Peng Yi, Jin Cheng, Junhui Sun, Mengshu Li, Peng Xiao, Kai Wang

**Affiliations:** ^1^ Department of Respiratory and Critical Care Medicine Center for Oncology Medicine the Fourth Affiliated Hospital of School of Medicine International School of Medicine International Institutes of Medicine Zhejiang University Yiwu 322000 China; ^2^ Zhejiang Key Laboratory of Precision Diagnosis and Treatment for Lung Cancer Yiwu 322000 China; ^3^ Department of Respiratory and Critical Care Medicine The First Affiliated Hospital of Wenzhou Medical University Wenzhou 325015 China; ^4^ Shanghai Fengxian District Central Hospital Shanghai 201400 China; ^5^ Department of oncology Beijing Chest Hospital Capital Medical University Beijing Tuberculosis and Thoracic Tumor Research Institute Beijing 101149 China; ^6^ Department of Oncology‐Pathology Karolinska Institutet Stockholm 17177 Sweden; ^7^ Department of cardiothoracic surgery Center for Oncology Medical the Fourth Affiliated Hospital of School of Medicine International School of Medicine International Institutes of Medicine Zhejiang University Yiwu 322000 China; ^8^ Department of Respiratory and Critical Care Medicine The Second Affiliated Hospital of Zhejiang University School of Medicine No. 1511, Jianghong Road Hangzhou 310003 China; ^9^ The Quzhou Affiliated Hospital of Wenzhou Medical University Quzhou People's Hospital Quzhou 324000 China; ^10^ The Affiliated Wuxi Center for Disease Control and Prevention of Nanjing Medical University Wuxi Center for Disease Control and Prevention Wuxi 214023 China; ^11^ Department of Reproductive Medicine Center The First Affiliated Hospital of Wenzhou Medical University Wenzhou 325015 China; ^12^ Sir Run Run Shaw Hospital Zhejiang University School of Medicine Hangzhou 310015 China

**Keywords:** cystine, immunotherapy, lung cancer, macrophage polarization, PD‐L1, tumor microenvironment

## Abstract

Immunotherapy with Immune Checkpoint Inhibitors (ICIs) has shown promising therapeutic effects in the treatment of lung cancer, the overall efficacy of PD‐1/PD‐L1 inhibitors is only 20%‐30%. Thus, more effective combination therapies are needed. This study finds that cystine and cysteine levels in tumor tissues of lung cancer patients are significantly higher than adjacent non‐tumor tissues. Cystine deficiency polarizes macrophages toward an M1 phenotype, secreting more TNF‐α, CXCL9, and CXCL10. However, using a cystine‐free diet marginally reduces the development of lung cancer in vivo. A cystine‐free diet slightly reduces lung cancer progression in vivo. Further studies show that cystine deprivation or erastin‐mediated transport inhibition increased PD‐L1 expression in macrophages both in vitro and in vivo. Combining a cystine‐free diet or IKE injection with PD‐L1 antibody treatment significantly inhibited subcutaneous tumor growth in mice. Mechanistic studies indicat that cystine deficiency‐induced GSH depletion activates NF‐κB in macrophages by reducing its glutathionylation. This effect can be reversed by replenishing GSH or using an NF‐κB inhibitor. At the same time, lung cancer patients with better responses to immunotherapy are found to have lower serum GSH levels. These findings suggest that targeting cystine metabolism combined with PD‐L1 inhibition is a promising therapeutic strategy.

## Graphical abstract

The tumor‐associated macrophages (TAMs) in lung cancer tissues exhibit active cystine metabolism. Restricting cystine concentration in macrophages induces M1 phenotypic polarization and upregulation of PD‐L1 expression through the GSH‐NF‐κB signaling pathway. A cystine‐free diet combined with PD‐L1 monoclonal antibody synergistically inhibits the progression of lung cancer in mice.

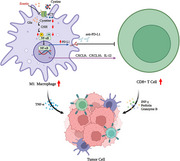

## 1 Introduction

Non‐small cell lung cancer (NSCLC) is the most common histologic subtype of lung cancer, accounting for ≈80–85% of all cases.^[^ ^1^ ^]^ In recent years, with the rapid advancement and application of immune checkpoint inhibitors (ICIs) represented by PD‐1/PD‐L1 antibodies in cancer treatment, many patients with advanced NSCLC have achieved a long‐term survival.^[^ ^2^, ^3^ ^]^ Several clinical studies have shown that ≈20% of patients with advanced lung cancer show a long‐term clinical benefit from ICI monotherapy or when combined with chemotherapy.^[^ ^4^, ^5^, ^6^, ^7^ ^]^ Accumulating evidence suggests that the composition of the tumor microenvironment (TME) significantly impacts the efficacy of ICI therapy. Along with the heterogeneous tumor cell populations, the TME encompasses infiltrating immune cells, vascular endothelial cells, fibroblasts, extracellular matrix, and various secreted factors.^[^ ^8^, ^9^ ^]^ Therefore, in‐depth exploration and understanding of the impact of the TME on lung cancer immunotherapy can contribute to more precise and effective enhancement of immunotherapy outcomes and improvement of patient prognosis.

Changes in amino acid metabolism in the TME affect the survival, proliferation, migration, and invasion of tumor cells, and influence the function and metabolic adaptability of immune cells.^[^ ^10^, ^11^ ^]^ In recent years, multiple studies have indicated that specific amino acids play crucial roles in regulating anti‐tumor processes. For instance, arginine is essential for the survival and effector function of T cells.^[^ ^12^ ^]^ Tumor‐associated macrophages (TAMs) can impair T cell function by upregulating arginase, which breaks down arginine into ornithine and urea.^[^ ^13^ ^]^ Glutamine serves as a primary energy source for tumor‐infiltrating immune cells, and its deficiency can lead to decreased function of Th1 cells and downregulation of the anti‐tumor effector molecule IFN‐γ.^[^ ^14^ ^]^ Leucine can be taken up by B cells in the colorectal cancer microenvironment, thereby promoting the expression of the immune‐suppressive cytokine TGF‐β1. Consequently, a high leucine diet has been found to inhibit the anti‐tumor immune response in colorectal cancer.^[^ ^15^ ^]^ However, the impact of abnormal amino acid metabolism in tumor tissues on the anti‐tumor immune therapy of NSCLC remains unclear.

To investigate the impact of amino acid metabolism on tumor immunity in the NSCLC microenvironment, an analysis of amino acid metabolism was performed in cancer and adjacent non‐tumor tissues from NSCLC patients. Both cystine and cysteine were significantly upregulated in cancer tissues. Subsequently, we delved into the regulation of cysteine metabolism on tumor immunity and its impact on NSCLC immunotherapy. Our results indicate that macrophages and tumor cells exhibit high expression of cystine transporters. Inhibiting cystine uptake in macrophages promotes M1 polarization and the release of anti‐tumor factors, but it also upregulates PD‐L1, thereby inhibiting the efficacy of immunotherapy. Combining a cystine‐restricted diet with PD‐L1 antibody synergistically enhances anti‐tumor immune responses and suppresses lung cancer progression. Our findings provide a potential combination therapy strategy for patients with lung cancer and establish the foundation for initial research on targeting cystine utilization in combination with checkpoint immunotherapy for clinical applications.

## 2 Results

### 2.1 Abnormal Cystine Metabolism is a Prominent Feature of Lung Cancer Microenvironment

**Figure 1:**
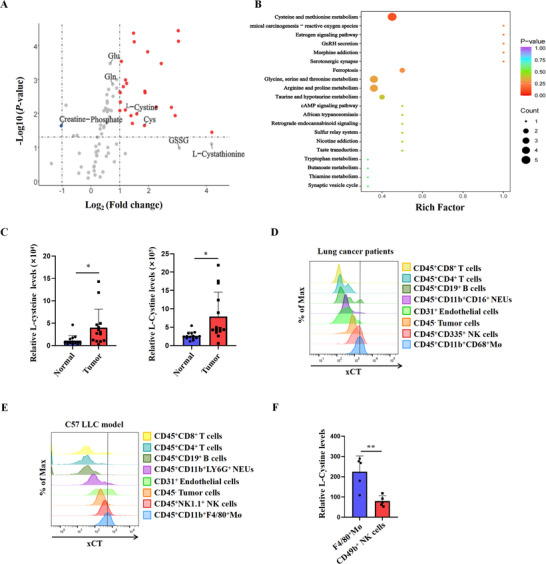
Lung cancer tissues exhibit active cysteine and cystine‐related metabolism. A) Differentially expressed metabolites were identified between lung cancer tissues and adjacent non‐cancerous tissues. |log2 (fold Change)| ≥ 1 and adjusted *P*‐value < 0.05 indicate the threshold for a significant difference. Red indicates upregulation, blue indicates downregulation, and gray indicates no significant difference. B) KEGG enrichment analysis for differential metabolites between lung cancer tissues and adjacent non‐cancerous tissues. The color of the points represents the *P*‐value, while the size indicates the number of differentially enriched metabolites (n = 14/group). C) Expression of L‐cysteine and L‐cystine in cancerous and adjacent non‐cancerous tissues (n = 14 patients). D,E) Flow cytometry analysis of xCT expression in various cell types obtained from patients with lung cancer or subcutaneous LLC tumors in mice (n = 3/group). F) Relative L‐cystine levels were measured in macrophages and NK cells purified from LLC tumors (n = 5/group). Data are shown as mean ± SD. ^*^, *P* < 0.05; ^**^, *P* < 0.01.

To clarify the alterations in amino acid metabolism in lung cancer, we collected 28 tumor tissues and the matched adjacent non‐tumor tissues from NSCLC patients for metabolomics analysis. By screening differential metabolites, we found that the levels of 24 amino acids and their metabolites were significantly increased in NSCLC tissues compared with normal tissues. Specifically, the levels of cysteine and cystine in NSCLC tissues were significantly higher than in adjacent non‐cancerous tissues, whereas creatine phosphate, which releases high‐energy phosphate bonds for energy supply, showed a significant decrease in tumor tissues compared to adjacent non‐cancerous tissues (**Figure** **1**A). When the differential metabolites were mapped to the Kyoto Encyclopedia of Genes and Genomes (KEGG) database, the “Cysteine‐methionine metabolism” was identified as the most enriched pathway (Figure 1B). Analysis of tumor and the paired adjacent non‐tumor tissues confirmed a prominent upregulation of cysteine and cystine levels in tumor tissues in the majority of NSCLC patients (Figure 1C). Next, we performed flow cytometry to investigate the cell‐specific expression of the cystine transporter system xc‐ (xCT) in clinical NSCLC tumor samples and LLC mouse subcutaneous tumor tissues. The results showed that xCT exhibited the highest expression on TAMs compared to tumor cells and other major immune populations, such as T cells, B cells, and NK cells (Figure 1D,E), gating strategy is shown in Figure S1 (Supporting Information). To further investigate, we isolated macrophages and NK cells from murine LLC subcutaneous tumors using F4/80‐ and CD49b‐specific magnetic beads for amino acid metabolomic profiling. The results revealed significantly higher levels of cystine in macrophages compared to NK cells (Figure 1F). The purity of the sorted cells is illustrated in Figure S2 (Supporting Information). These results demonstrate that the abnormally increased cystine/cysteine content is a hallmark of NSCLC microenvironment, which may support tumor development through regulating macrophage functions.

### 2.2 Cystine Deficiency Polarizes Macrophages toward an Anti‐Tumor M1 Phenotype

**Figure 2:**
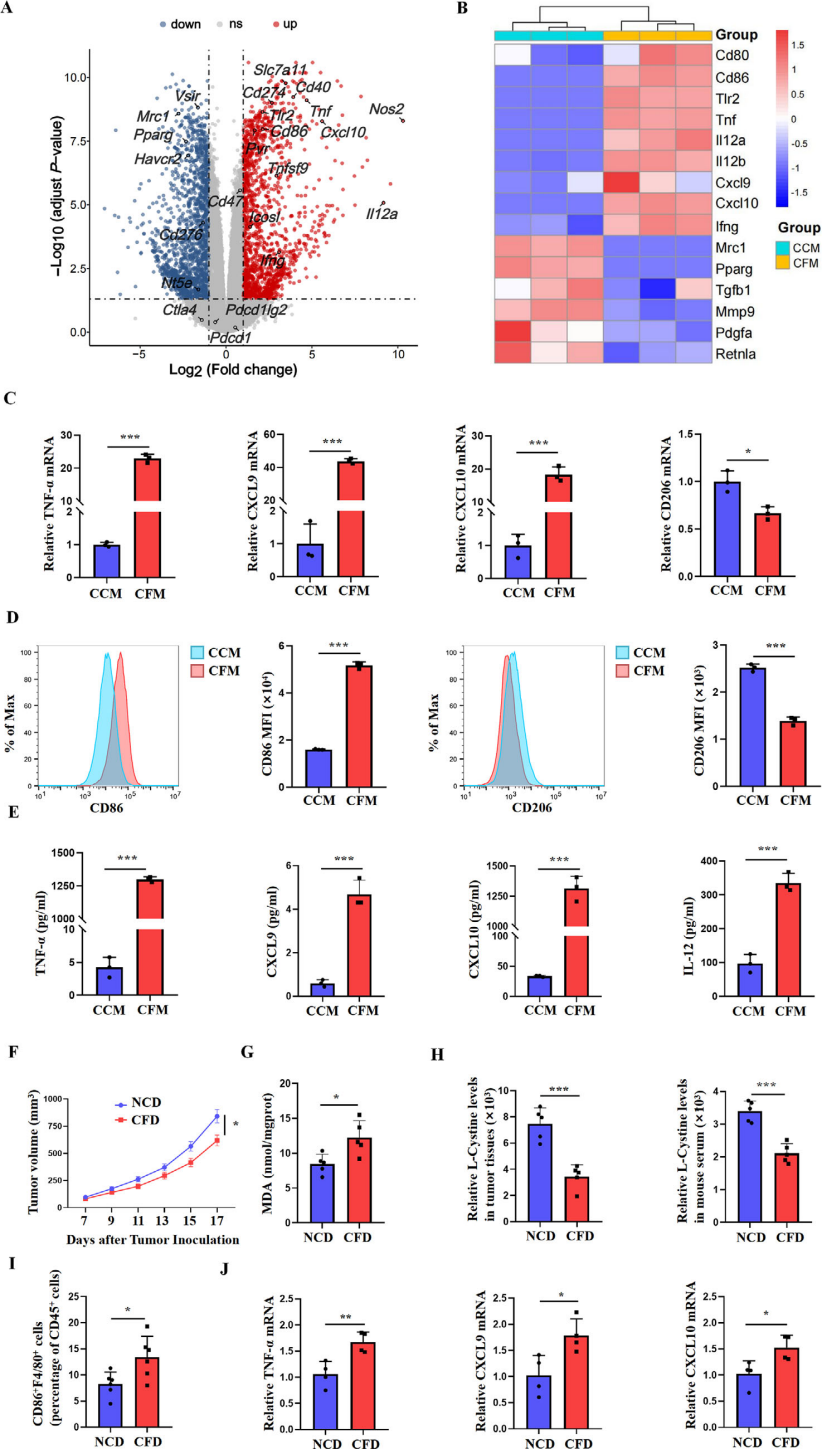
Cystine deficiency induces polarization of macrophages toward M1 phenotype. A) Volcano plot showing differential gene expression between macrophages in the CCM and CFM groups. |log2 (fold change)| ≥ 1 and adjusted *P* < 0.05 represent the threshold for a significant difference. Each point in the plot represents a gene. Red indicates upregulated transcription, blue indicates downregulated transcription, and gray indicates detected gene transcription expression but with no significant difference (n = 3/group). B) Heatmap illustrating the relative expression of genes related to macrophage polarization between the normalized CCM group and CFM group. In the heatmap, red indicates higher relative expression, while blue indicates lower relative expression (n = 3/group). C) RT‐PCR analysis was conducted to assess the relative expression of TNF‐α, CXCL9, CXCL10, and CD206 in macrophages after culturing in CCM or CFM for 24 h (n = 3/group). D) Histograms and statistical charts display the levels of CD86 and CD206 in macrophages after culturing in CCM or CFM for 24 h using flow cytometry (n = 3/group). E) ELISA was used to detect changes in the levels of TNF‐α, CXCL9, CXCL10, and IL‐12 in the supernatant of macrophages from the peritoneal cavity after 24 h of culture in CFM (n = 3/group). F) Growth curve of subcutaneous LLC tumors in NCD and CFD treatment groups (n = 5/group). Data are shown as mean ± SEM. G) Levels of MDA in the LLC model treated with NCD and CFD (n = 5/group). H) Relative L‐cystine levels were measured in tumor tissues and serum from LLC models treated with NCD or CFD (n = 5/group). I) Statistical histogram depicting the proportion of infiltrating CD86^+^F4/80^+^ M1 type TAMs in subcutaneous LLC tumors treated with NCD and CFD (n = 6/group). J) RT‐PCR analysis was conducted to measure the relative expression levels of TNF‐α, CXCL9, and CXCL10 in the LLC model treated with NCD and CFD (n = 4/group). Data are representative of three independent experiments. Data are presented as mean ± SD. NS, Not Significant; ^*^, *P* < 0.05; ^**^, *P* < 0.01; ^***^, *P* < 0.001.

Next, we evaluated the potential impact of cystine on macrophage function. To this end, murine peritoneal macrophages were isolated (Figure S3A, Supporting Information) and cultured in cystine‐free medium (CFM) or complete culture medium (CCM). No change in viability of macrophages was observed (Figure S3B,C, Supporting Information). After performing RNA sequencing (RNA‐seq), we identified 1626 significantly upregulated and 1731 significantly downregulated genes in CFM macrophages compared with CCM macrophages. Among these, the expression of genes such as *Slc7a11*, *Cd274*, and *Cd40* was upregulated, while genes including *Pparg* and *Vsir *were downregulated (**Figure** **2**A). Notably, cystine deficiency led to a prominent upregulation of M1‐like genes, such as *Tnf‐α*, *Cxcl9*, and *Cxcl10*. In contrast, the M2‐like gene *Mrc1* was downregulated (Figure 2B). After CFM treatment, the mRNA levels of TNF‐α, CXCL9, and CXCL10 in peritoneal macrophages were significantly upregulated, and the expression of CD206 was decreased (Figure 2C). Similar to the cystine‐free condition, inhibiting cystine transport by erastin significantly upregulated the mRNA levels of multiple M1‐like genes (Figure S4A, Supporting Information). Flow cytometry analysis also confirmed increased CD86 and decreased CD206 expression on CFM macrophages (Figure 2D). In addition, the protein levels of TNF‐α, CXCL9, CXCL10, and IL‐12 were significantly upregulated in the supernatants of CFM macrophages (Figure 2E). Similarly, treatment of THP1‐derived human macrophages with CFM significantly upregulated TNF‐α, CXCL9, and CXCL10 (Figure S4B, Supporting Information). Further, we co‐cultured LLC tumor cells with peritoneal macrophages at ratios of 1:1 and 3:1, stimulating them with CCM or CFM, respectively, and measured the expression levels of CD86, CD206, TNF‐α, and CXCL10. Flow cytometry and RT‐PCR analyses revealed that both co‐culture ratios increased CD86, TNF‐α, and CXCL10 expression while decreasing CD206 expression, mirroring the effects observed in macrophages stimulated with CFM alone (Figure S5A—C, Supporting Information).

We then investigated the impact of cystine deficiency on polarized macrophages. To this end, macrophages were stimulated with LPS+IFN‐γ or IL‐4 to induce their M1 or M2 differentiation, respectively, followed by CFM treatment for 24 h. Surface levels of CD86 were significantly increased in both M1 and M2 macrophages, while CD206 levels were significantly decreased (Figure S6A, Supporting Information). A significant increase in TNF‐α secretion was observed in both M1 and M2 macrophages, alongside elevated secretion of CXCL9 and CXCL10 in M2 macrophages cultured in CFM (Figure S6B, Supporting Information), suggesting that cystine deficiency endows M2‐polarized macrophages with an M1 phenotype.

Given that limiting cystine utilization polarizes macrophages toward an anti‐tumor M1 phenotype, we hypothesized that a cystine‐restricted diet may have a therapeutic effect on lung tumor growth in vivo. Therefore, we established a murine subcutaneous LLC tumor model, and fed mice on a cystine‐free diet (CFD) or a normal control diet (NCD). The growth of LLC tumors was significantly inhibited in CFD mice (Figure 2F). We conducted a malondialdehyde (MDA) detection assay on tumor tissues from each group to assess the extent of lipid peroxidation damage. The results indicated that the MDA content in tumors from the CFD diet group was elevated (Figure 2G). Additionally, we measured the levels of cystine in both tumor tissues and serum from the two groups using amino acid metabolomics. Our findings revealed that cystine levels were significantly reduced in both tumors and serum of the CFD group (Figure 2H). Furthermore, compared to NCD mice, the percentage of CD86^+^F4/80^+^M1‐like TAMs and the expression levels of M1 markers TNF‐α, CXCL9, and CXCL10 were significantly higher in CFD mice (Figure 2I,J). These results suggest that decreased cystine availability polarizes macrophages toward an anti‐tumor M1 phenotype.

### 2.3 Limiting Cystine Utilization Induces PD‐L1 Expression in Macrophages

**Figure 3:**
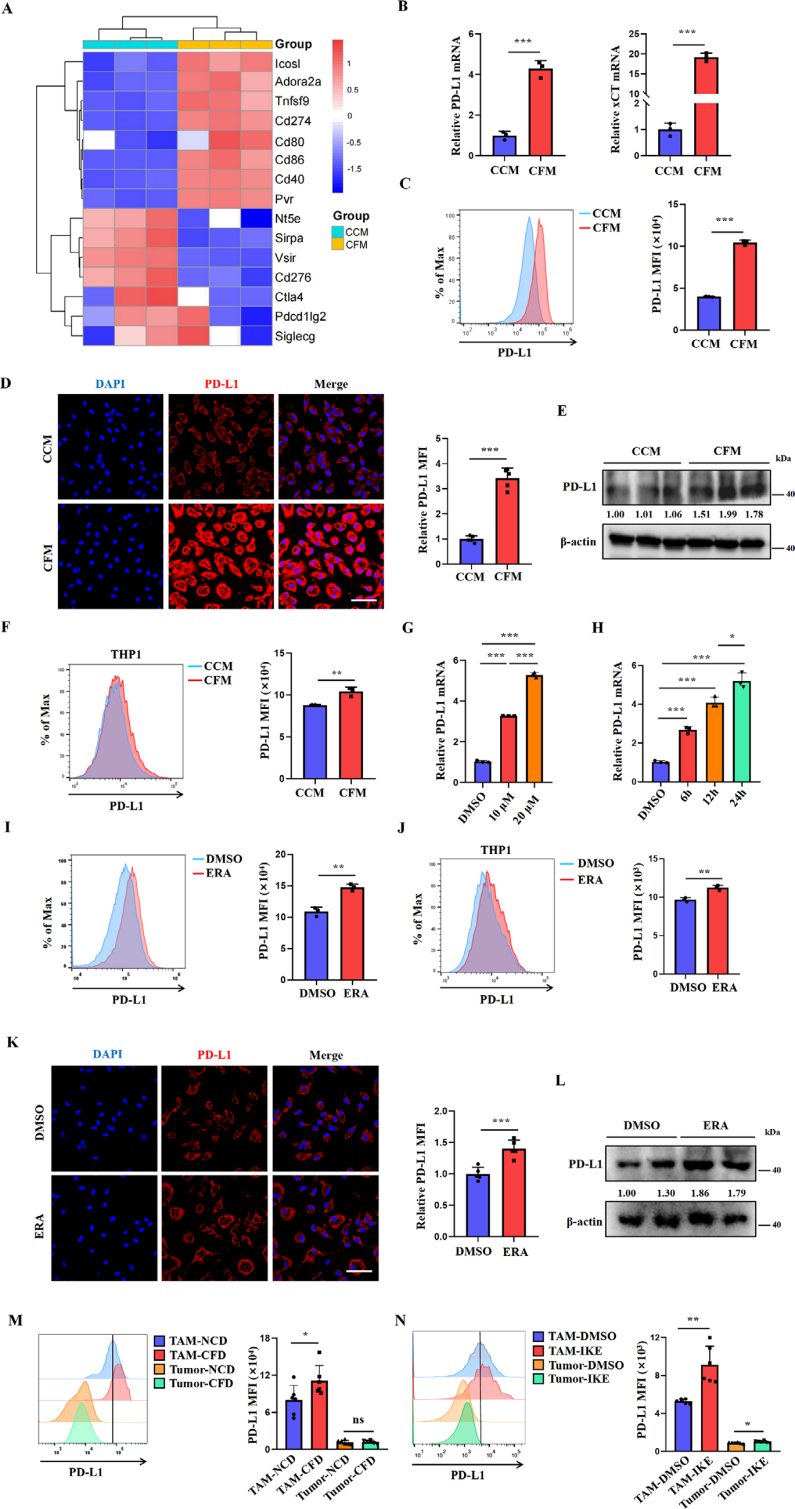
Cystine deficiency upregulates PD‐L1 expression in macrophages. A) Heatmap illustrating the relative expression of immune‐regulatory genes in macrophages between the normalized CCM group and CFM group. In the heatmap, red indicates a higher relative expression, while blue indicates a lower relative expression (n = 3/group). B,C) RT‐PCR analysis and flow cytometry were conducted to measure the relative expression levels of PD‐L1 and xCT in macrophages after culturing in CCM or CFM for 24 h (n = 3/group). D,E) Immunofluorescence assay and western‐blot were conducted to detect PD‐L1 expression in macrophages after culturing in CCM or CFM for 24 h (Scale bar = 50 µm). F) Histograms and statistical charts illustrate the levels of PD‐L1 protein in THP‐1 cells after culturing in CFM (n = 3/group). G–I,K,L) RT‐PCR, flow cytometry, immunoblotting, and immunofluorescence assays were used to detect the expression of PD‐L1 in macrophages of different groups (n = 3/group, 20 µm erastin, Scale bar = 50 µm). J) Histograms and statistical charts depict PD‐L1 protein levels in THP‐1 cells after treatment with erastin (n = 3/group). M,N) The expression of PD‐L1 in tumor cells and TAMs in LLC subcutaneous tumors of the different treatment groups (n = 6/group). Data are representative of three independent experiments. Data are presented as mean ± SD. NS, Not Significant; ^*^, *P* < 0.05; ^**^, *P* < 0.01; ^***^, *P* < 0.001.

Although cystine restriction exerts a tumor‐suppressive function, its therapeutic efficacy is relatively mild and unsatisfactory. Therefore, it is possible that the decreased cystine availability induces specific compensatory mechanisms, thus masking the therapeutic potential of cystine deprivation. A comprehensive examination of our RNA sequencing data indicated that genes including *Ctla4*, *Cd276*, and *Sirpα* exhibited downregulation, whereas the expression level of *Cd274* was significantly elevated in CFM macrophages compared to CCM macrophages (**Figure** **3**A). RT‐PCR results also verified the increased PD‐L1 and xCT expression in CFM macrophages (Figure 3B). The increased PD‐L1 protein expression upon cystine deprivation was further confirmed by flow cytometry, immunofluorescence, and immunoblotting (Figure 3C–E). This phenomenon was also confirmed in human THP1‐derived macrophages (Figure 3F).

In addition to directly depriving cystine using CFM, we further treated macrophages with erastin. As shown in Figure 3G,H, erastin significantly increased the mRNA levels of PD‐L1 in a time‐ and dose‐dependent manner. The ability of erastin to promote the protein expression of PD‐L1 was further validated by flow cytometry, immunofluorescence, and immunoblotting (Figure 3I,K,L). The PD‐L1‐inducing effect of erastin was also observed in human THP1‐derived macrophages (Figure 3J). Interestingly, in contrast to macrophages, treatment with CFM or erastin failed to increase PD‐L1 expression in lung cancer cell lines, including A549, PC9, and H2170 (Figure S7A–C, Supporting Information). In LLC tumor‐bearing mice, we found that a cystine‐restricted diet significantly increased PD‐L1 expression in TAMs but not in CD45‐negative cells (Figure 3M). Likewise, Imidazole Ketone Erastin (IKE), a potent and selective system xc⁻ inhibitor, significantly upregulated PD‐L1 expression in TAMs (Figure 3N). These findings indicate that restricting cystine intake drives PD‐L1 expression in macrophages, which may mask the therapeutic potential of cystine deprivation.

### 2.4 Cystine Restriction Augments the Therapeutic Effectiveness of PD‐L1 Blockade

**Figure 4:**
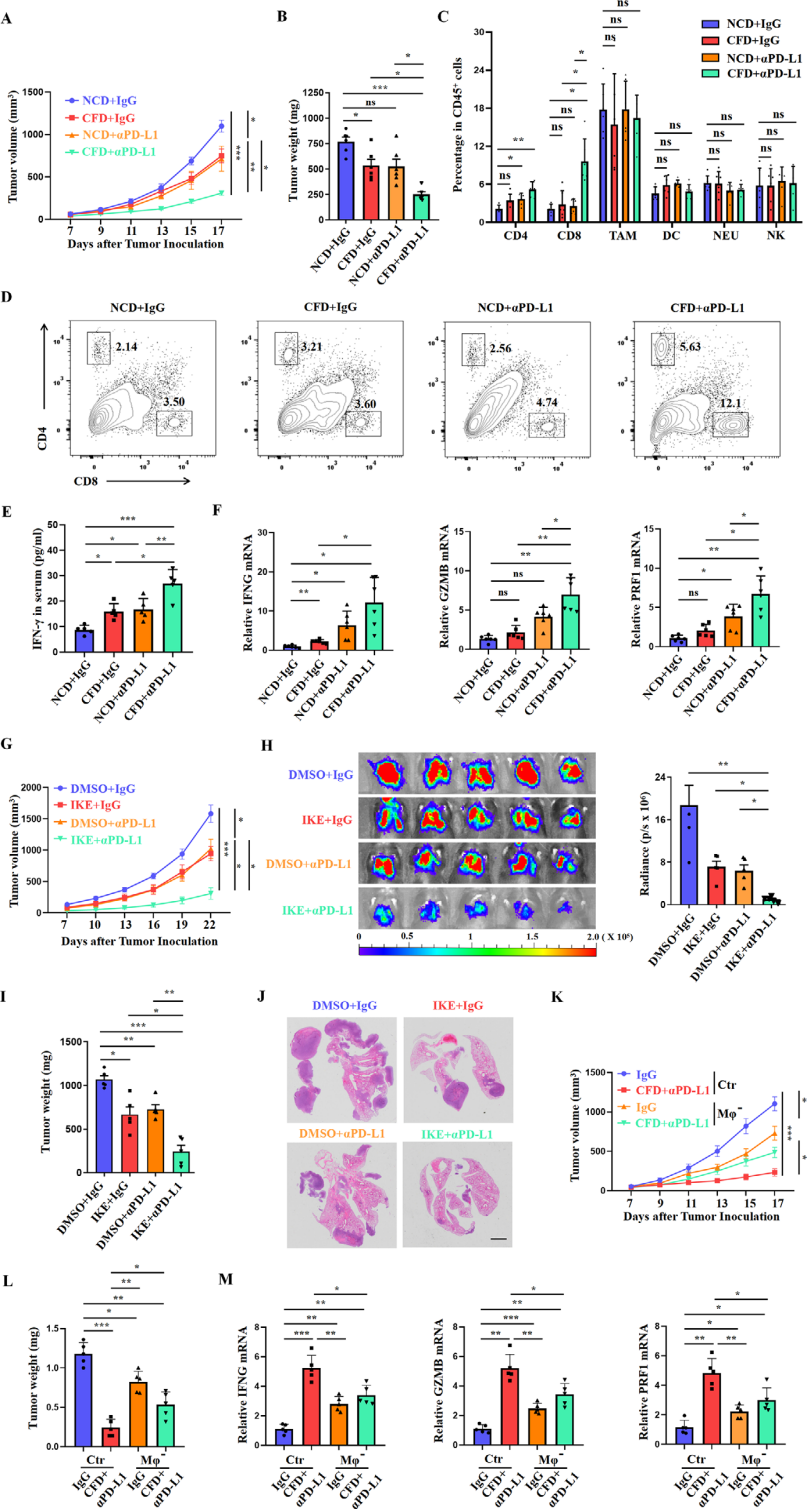
Restricting cystine combined with PD‐L1 antibody therapy synergistically inhibits the progression of lung cancer. A,B) Growth curve and tumor weight of subcutaneous LLC tumors in mice treated with NCD, CFD, αPD‐L1, and CFD+αPD‐L1(n = 6/group). Data are shown as mean ± SEM or mean ± SD, respectively. C) The percentages of CD4^+^T cells, CD8^+^T cells, CD11b^+^F4/80^+^ macrophages, CD11b^+^CD11c^+^ dendritic cells, CD11b^+^Ly6G^+^ neutrophils, and NK1.1^+^ natural killer cells in subcutaneous LLC tumors were analyzed by flow cytometry (gated on CD45^+^ cells) (n = 6/group). D) A representative flow cytometry scatter plot showing infiltrating CD4^+^ and CD8^+^ cells in subcutaneous LLC tumors (gated on CD45^+^ cells). E) Levels of IFN‐γ in the peripheral blood were detected by ELISA (n = 5/group). F) RT‐PCR analysis was conducted to measure the relative expression levels of IFNG, GZMB, and PRF1 in the LLC model treated with NCD, CFD, αPD‐L1, and CFD+αPD‐L1 (n = 6/group). G) Growth curve of subcutaneous LLC tumors in mice treated with DMSO, IKE, αPD‐L1, and IKE+αPD‐L1 (n = 5/group). Data are shown as mean ± SEM. H) Bioluminescence live imaging and statistical charts for the LLC metastasis model (n = 5/group). I) Tumor weight of subcutaneous LLC tumors in mice treated with DMSO, IKE, αPD‐L1, and IKE+αPD‐L1 (n = 5/group). J) Hematoxylin and eosin‐stained lung tissue section of LLC tumor‐bearing mice. Scale bar = 2 mm (n = 5/group). K,L) Growth curve and tumor weight of subcutaneous LLC tumors in different mice treatment groups (n = 5/group). Data are shown as mean ± SEM or mean ± SD, respectively. M) RT‐PCR analysis was conducted to measure the relative expression levels of IFNG, GZMB, and PRF1 in the LLC model treated with NCD, CFD+αPD‐L1, NCD+Clodronate liposomes+IgG, and CFD+Clodronate liposomes+αPD‐L1 (n = 5/group). Data are shown as mean ± SD. NS, Not Significant; ^*^, *P* < 0.05; ^**^, *P* < 0.01; ^***^, *P* < 0.001.

Since restricting cystine intake induces both anti‐tumor phenotypes and PD‐L1 expression in macrophages, we next investigated if PD‐L1 blockade could achieve a better therapeutic effectiveness in the context of low cystine availability. While CFD or anti‐PD‐L1 (αPD‐L1) treatment alone marginally inhibited tumor growth and reduced tumor weight, αPD‐L1 administration in mice on a CFD diet robustly reduced tumor progression (**Figure** **4**A,B). Serum albumin (ALB) levels, blood urea nitrogen (BUN), creatinine (CRE), total cholesterol (TCHO), creatine phosphokinase (CPK), glutamic oxalacetic transaminase (GOT), and glutamic‐pyruvic transaminase (GPT) were comparable among groups, thus excluding the therapy‐induced adverse effects (Figure S8A, Supporting Information). We also compared the proportions of CD4^+^ T cells, CD8^+^ T cells, and NK cells in the spleens of the four groups of mice and found no significant differences (Figure S8B, Supporting Information). M1‐like macrophage‐derived factors, such as CXCL9 and CXCL10, are essential for the recruitment of CD8^+^ T cells and their production of cytotoxic mediators.^[^ ^16^, ^17^ ^]^ We found that the percentages of tumor‐infiltrating CD8⁺ T cells were significantly higher in αPD‐L1‐treated CFD mice compared to IgG‐treated CFD mice, αPD‐L1‐treated NCD mice, and IgG‐treated NCD mice, demonstrating an obvious synergistic effect. The percentages of CD4⁺ T cells, macrophages, dendritic cells, natural killer cell, and neutrophil cells were comparable among groups (Figure 4C,D). ELISA assay also confirmed a significant increase in serum IFN‐γ levels in αPD‐L1‐treated CFD mice compared to other groups (Figure 4E). In agreement, the expression levels of CD8^+^ T cell‐derived cytotoxic mediators, such as IFN‐γ, granzyme B, and perforin, showed a robust increase only in the tumor tissues of αPD‐L1‐treated CFD mice (Figure 4F).

To further address whether pharmaceutical inhibition of cystine transport can sensitize αPD‐L1 therapy, LLC mice were treated with IKE, αPD‐L1, or in combination. Compared to the control group and mono‐treated mice, the combination therapy with IKE and αPD‐L1 robustly inhibited tumor growth and reduced tumor weight (Figure 4G,I). In a metastatic LLC tumor model, the pulmonary tumor burden was significantly reduced in mice treated with IKE plus αPD‐L1, when compared with control and monotherapy groups (Figure 4H). Histological analysis revealed a significantly reduced area of metastatic nodules in the combination therapy group (Figure 4J). Clodronate liposomes (CLs) are commonly used to eliminate macrophages in mice. To confirm whether the cysteine‐low microenvironment enhances the efficacy of PD‐L1 monoclonal antibodies in a macrophage‐dependent manner, we treated mice with CLs. Compared to the CFD plus αPD‐L1 group, the antitumor effect was significantly weakened after macrophage depletion combined with CFD+plus αPD‐L1 (Figure 4K,L), and levels of IFN‐γ, granzyme B, and perforin were also significantly downregulated (Figure 4M). This indicates that the enhancement of PD‐L1 monoclonal antibodies by the cysteine‐low microenvironment mainly depends on macrophages. In summary, our results show that a low cystine tumor microenvironment is favorable for the optimal effectiveness of PD‐L1 blockade therapy, and this treatment relies on macrophages.

### 2.5 Cystine Deficiency Reprogrammes Macrophage Functions through GSH Depletion

**Figure 5:**
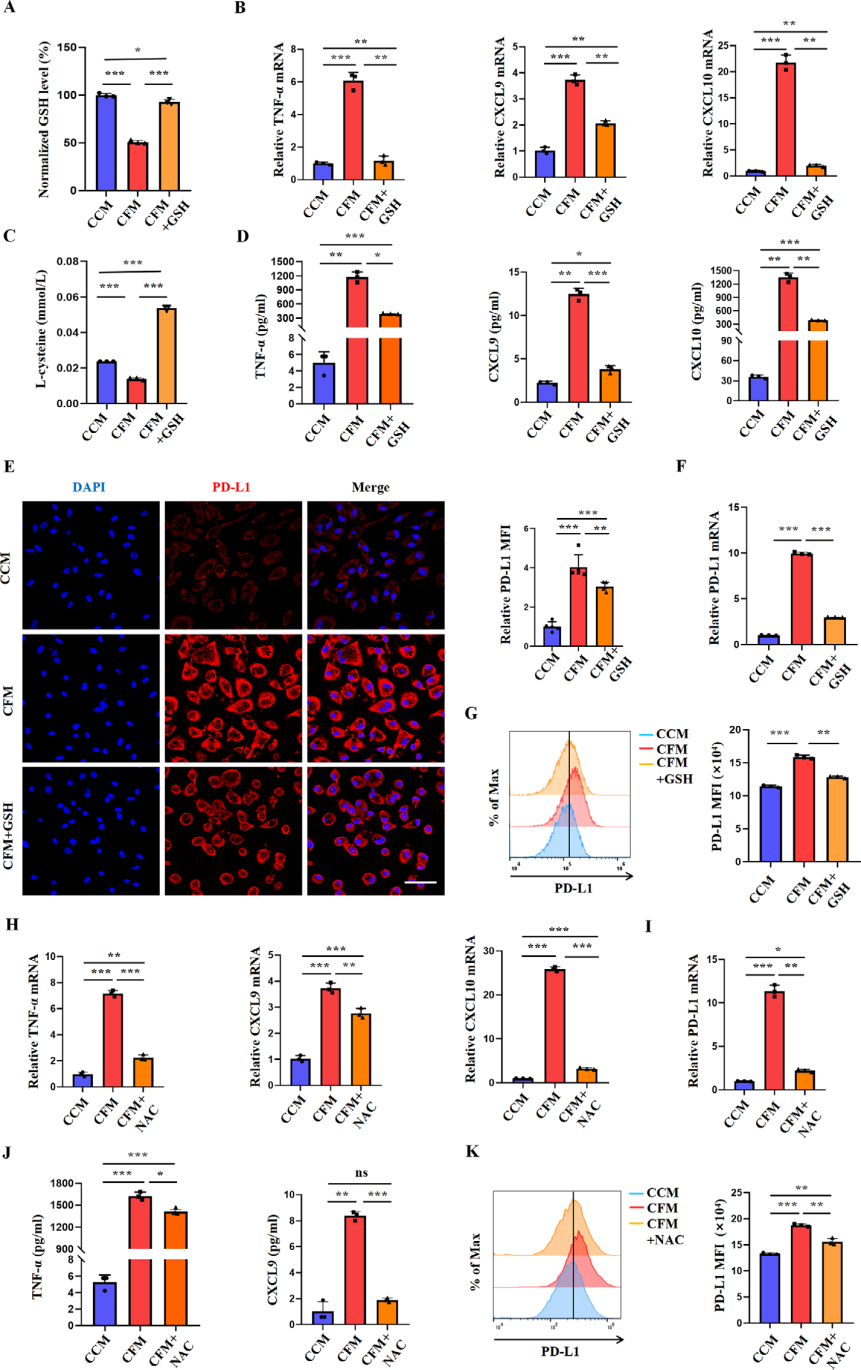
Upregulated PD‐L1 expression and M1 polarization in macrophages under cystine deficiency are related to decreased GSH levels. A) Relative GSH levels in mouse peritoneal macrophages cultured for 24 h in CCM, CFM, and CFM+GSH (5 mm) (n = 3/group). B,D) RT‐PCR analysis and ELISA were conducted to measure the relative expression levels of TNF‐α, CXCL9, and CXCL10 in peritoneal macrophages treated with CCM, CFM, or CFM+GSH for 24 h (n = 3/group). C) Relative L‐cysteine levels were measured in mouse peritoneal macrophages cultured for 24 h in CCM, CFM, and CFM+GSH (5 mm) (n = 3/group). E–G) After treating peritoneal macrophages with CCM, CFM, and CFM+GSH for 24 h, and an immunofluorescence assay, RT‐PCR, and flow cytometry were conducted to detect PD‐L1 expression. (Scale bar = 50 µm, n = 3/group). H,J) RT‐PCR and ELISA was used to detect the levels of TNF‐α, CXCL9, and CXCL10 in peritoneal macrophages treated with CCM, CFM, or CFM+NAC for 24 h (n = 3/group) I,K) After treating peritoneal macrophages with CCM, CFM, and CFM+NAC for 24 h, RT‐PCR and flow cytometry was used to detect PD‐L1 protein expression (n = 3/group). Data are representative of three independent experiments. Data are presented as mean ± SD. NS, Not Significant; ^*^, *P* < 0.05; ^**^, *P* < 0.01; ^***^, *P* < 0.001.

Upon transport into cells, cystine is converted to cysteine, which serves as a rate‐limiting amino acid in GSH biosynthesis. Therefore, we investigated whether cystine deficiency‐induced functional changes in macrophages were associated with decreased GSH synthesis. As expected, GSH levels were significantly decreased in macrophages cultured in CFM or treated with erastin compared to the control group (**Figure** **5**A; Figure S9A, Supporting Information). Supplementation with GSH in the CFM group partially restored intracellular GSH levels (Figure 5A). Similarly, the addition of exogenous GSH increased cellular cysteine levels (Figure 5C). Importantly, exogenous GSH supplementation significantly reversed CFM‐induced enhancement of TNF‐α, CXCL9, and CXCL10 in macrophages, as assessed by both mRNA and protein levels (Figure 5B,D; Figure S9B, Supporting Information). Likewise, GSH restored the mRNA and protein levels of PD‐L1 in CFM‐cultured macrophages (Figure 5E–G; Figure S9C,D, Supporting Information). Similarly, N‐Acetyl‐L‐cysteine (NAC), a GSH precursor which can be transported into cells independent of xCT, prevented the increase in the expression of M1 markers and PD‐L1 in CFM‐ or erastin‐treated macrophages (Figure 5H–K; Figure S9E–G, Supporting Information).

Since cystine deficiency generally causes an upregulation of intracellular ROS levels, we next investigated if ROS is involved in cystine‐regulated macrophage functions. To this end, we treated macrophages with FIN56, RSL3, or FINO2, which serve as ROS inducers (also known as ferroptosis inducers like erastin). As shown in Figure S10A (Supporting Information), although FIN56, RSL3, and FINO2 significantly increased intracellular ROS levels, they cannot deplete GSH in macrophages (Figure S10B, Supporting Information). In addition, none of these ROS inducers significantly altered the expression of PD‐L1 in macrophages (Figure S10C, Supporting Information). Moreover, when we treated macrophages with a ferroptosis inhibitor ferrostatin‐1 (Fer‐1), which reduces intracellular ROS production without affecting GSH levels, it was found that Fer‐1 did not effectively reverse the phenotypic changes of macrophages induced by CFM or erastin (Figure S10D—I, Supporting Information). Therefore, cystine induced functional reprogramming of macrophages through interrupting GSH synthesis.

### 2.6 Cystine Suppresses M1 Polarization of Macrophages by Inhibiting NF‐κB Signaling

**Figure 6:**
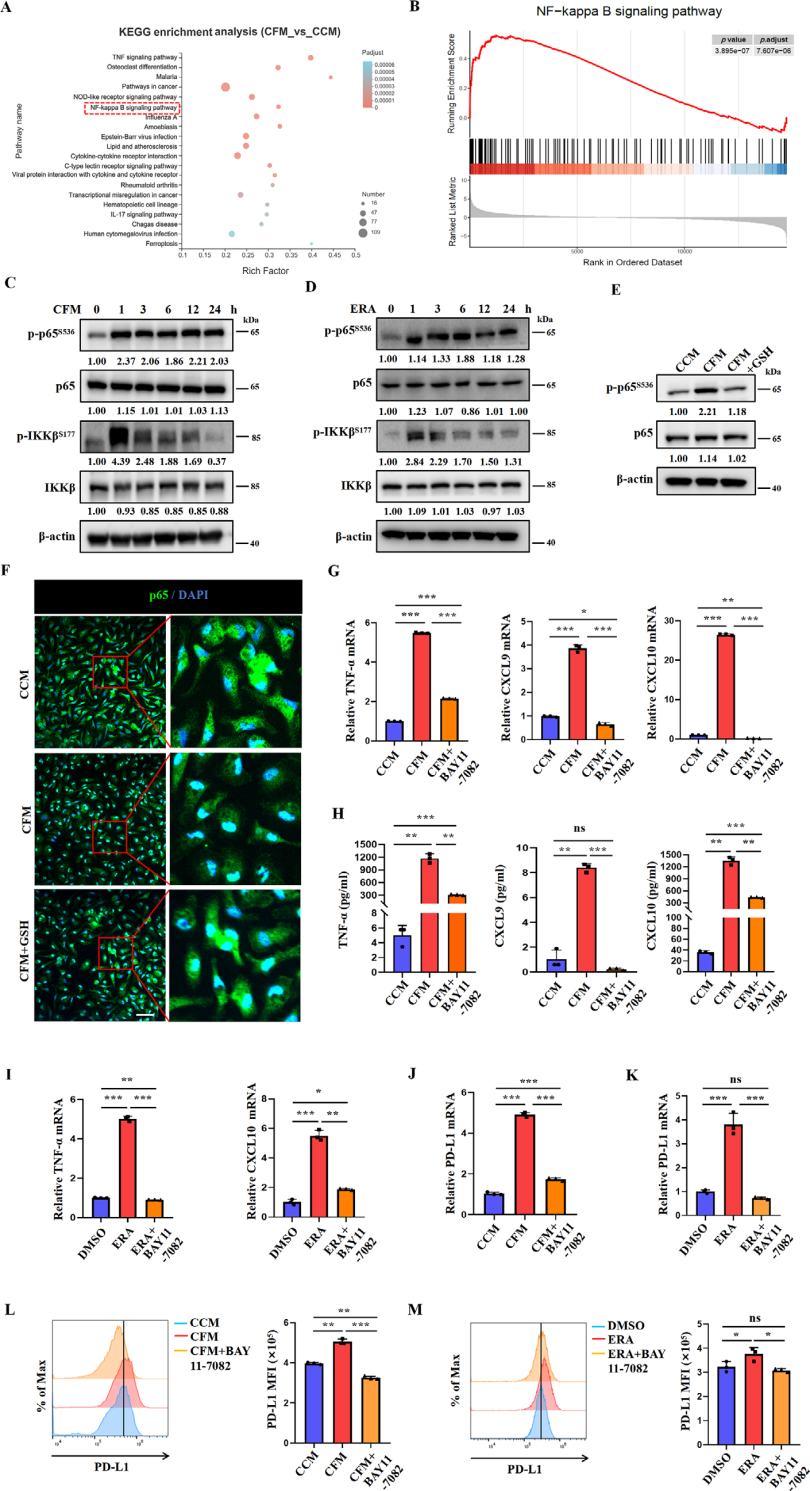
Cystine deficiency upregulates PD‐L1 expression and M1 polarization through activation of the NF‐κB signaling pathway in macrophages. A) Bubble chart illustrating KEGG enrichment analysis of differentially expressed genes in macrophages cultured in CCM and CFM. B) GSEA for the NF‐κB signaling pathway in macrophages cultured in CCM and CFM. C,D) Levels of phosphorylated p65 and phosphorylated IKK in macrophages treated with CFM or erastin for 0, 1, 3, 6, 12, and 24 h were detected by immunoblotting. E,F) Immunoblotting and immunofluorescence assays were used to detect the expression and distribution of p65 in macrophages treated with CCM, CFM, or CFM+GSH for 24 h. Scale bar = 50 µm. G,H) Macrophages were pre‐treated with BAY 11–7082 for 6 h and then RT‐PCR analysis and ELISA were conducted to measure the relative expression levels of TNF‐α, CXCL9, and CXCL10 in peritoneal macrophages treated with CCM, CFM, or CFM+BAY 11–7082 for 24 h (n = 3/group). I) RT‐PCR analysis was conducted to measure the relative expression levels of TNF‐α, CXCL10 in peritoneal macrophages (n = 3/group). J–M) Macrophages were pre‐treated with 5 µm BAY 11–7082 for 6 h and cultured in CCM, CFM, and CFM+BAY 11–7082 or DMSO, erastin, and erastin+BAY 11–7082 for 24 h. RT‐PCR and Flow cytometry were used to detect PD‐L1 expression (n = 3/group). Data are representative of three independent experiments. Data are presented as mean ± SD. NS, Not Significant; ^*^, *P* < 0.05; ^**^, *P* < 0.01; ^***^, *P* < 0.001.

To investigate the molecular mechanisms underlying cystine‐regulated macrophage functionality, KEGG enrichment analysis was performed using the differentially expressed genes (DEGs) between CCM‐ and CFM‐cultured macrophages from our RNA‐seq data. The results revealed that the NF‐κB signaling pathway, a master pathway that controls M1 polarization, was among the top‐ranked KEGG pathways. This finding was further confirmed by GSEA enrichment analysis (**Figure** **6**A,B). Immunoblotting revealed that CFM or erastin treatment induced a rapid and sustained phosphorylation of p65 (the transcription activation subunit of NF‐κB) and IKK in macrophages (Figure 6C,D). The enhanced p65 activation was dampened by exogenous GSH supplementation (Figure 6E). Consistently, nuclear translocation of p65 in macrophages cultured in CFM was significantly increased and attenuated by GSH supplementation (Figure 6F). Notably, the increased p65 phosphorylation was not observed in CFM‐cultured A549, PC9, and H2170 lung cancer cell lines (Figure S11A–C, Supporting Information), nor was it induced by treatment with RSL3, FIN56, or FINO2 in macrophages (Figure S11D–F, Supporting Information). Moreover, BAY 11–7082, a NF‐κB inhibitor, prevented the CFM‐ or erastin‐induced increase in TNF‐α, CXCL9, and CXCL10 in macrophages, as assessed by RT‐PCR and ELISA (Figure 6G–I). Besides, the elevated expression of PD‐L1 in CFM‐ or erastin‐treated macrophages was also counteracted by NF‐κB inhibition (Figure 6J–M). Buthionine sulfoximine (BSO) is an inhibitor that depletes intracellular glutathione levels. Treatment with BSO resulted in the upregulation of CD80, CD86, and PD‐L1 expression in peritoneal macrophages, and this phenomenon was alleviated by the addition of NAC and BAY 11–7082 (Figure S12A–C, Supporting Information). Similarly, nuclear translocation of p65 in macrophages was observed following BSO treatment (Figure S12D, Supporting Information). WB results also indicated an increased expression of phosphorylated p65 and IKK in macrophages (Figure S12E, Supporting Information). However, increased p65 phosphorylation was not observed in BSO‐treated A549, PC9, and H2170 lung cancer cell lines (Figure S12F–H, Supporting Information). Therefore, low cystine condition triggers functional reprogramming of macrophages through activating the NF‐κB signaling pathway.

### 2.7 Glutathionylation of NF‐κB Inhibits its Nuclear Translocation and Activation

**Figure 7:**
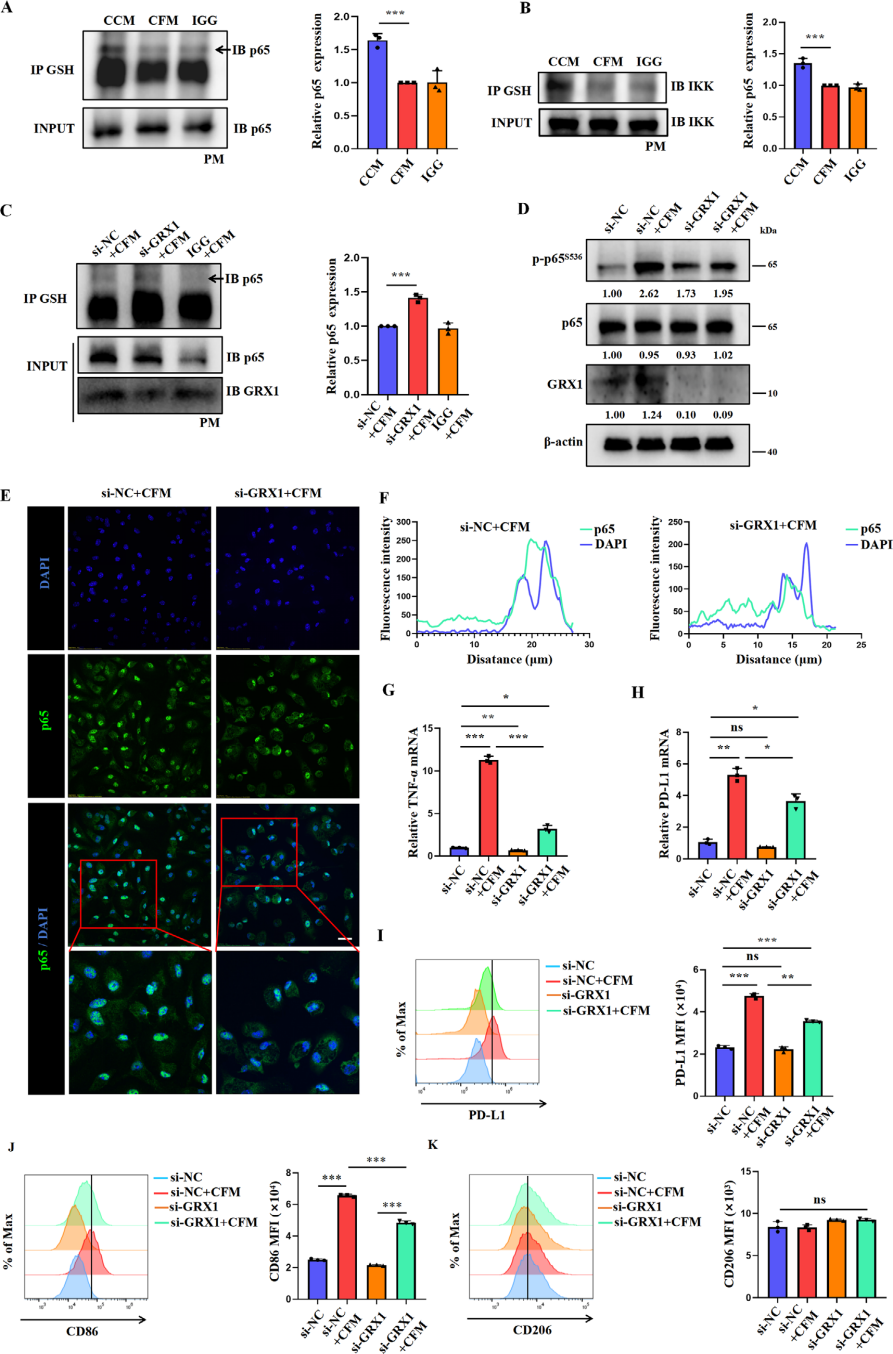
The regulation of nuclear translocation of NF‐κB and downstream transcription by glutathionylation modification. A,B) Co‐immunoprecipitation was used to detect the glutathionylation modification levels of p65 and IKK in macrophages treated with CCM and CFM, the protein quantification analysis was conducted using ImageJ (n = 3/group). C) Co‐immunoprecipitation was used to detect the glutathionylation modification levels of p65 in macrophages treated with si‐NC+CFM, and si‐GRX1+CFM, the protein quantification analysis was conducted using ImageJ (n = 3/group). D) Macrophages were interfered with si‐NC, and si‐GRX1. Levels of phosphorylated p65 in macrophages treated with CCM and CFM for 24 h were detected by immunoblotting. E,F) Macrophages were interfered with si‐NC, and si‐GRX1. An immunofluorescence assay was used to detect the expression and distribution of p65 in macrophages and quantifying fluorescence intensity using ImageJ. Scale bar = 50 µm. G,H) Macrophages were interfered with si‐NC and si‐GRX1, and treated with CCM or CFM for 24 h. RT‐PCR was used to detect TNF‐α and PD‐L1 expression (n = 3/group). I–K) Macrophages were interfered with si‐NC and si‐GRX1, and treated with CCM or CFM for 24 h. Flow cytometry was used to detect PD‐L1, CD86 and CD206 expression (n = 3/group). Data are representative of three independent experiments. Data are presented as mean ± SD. NS, Not Significant; ^*^, *P* < 0.05; ^**^, *P* < 0.01; ^***^, *P* < 0.001.

We have previously shown that GSH‐mediated glutathionylation plays a key role in regulating protein functions.^[^ ^18^ ^]^ Therefore, we interrogated if GSH regulates NF‐κB activation by modulating its glutathionylation. Co‐immunoprecipitation revealed a significant reduction in glutathionylation modification of p65 and IKK after CFM treatment (**Figure** **7**A,B). Our group has identified glutaredoxin‐1 (GRX1) as a crucial enzyme that mediates protein deglutathionylation.^[^ ^18^ ^]^ Therefore, we knocked down the expression of GRX1 in macrophages, then cultured them in CFM (Figure S13A,B, Supporting Information). GRX1 knockdown increased the glutathionylation of p65 in CFM macrophages (Figure 7C). In addition, the phosphorylation of p65 was markedly decreased in GRX1^low^ CFM macrophages compared with control CFM macrophages (Figure 7D). Congruently, immunofluorescence results showed a prominent decrease in nuclear translocation of p65 after GRX1 knockdown (Figure 7E,F). Functional assays showed that knocking down GRX1 significantly attenuated the upregulation of TNF‐α, CD86, and PD‐L1 in CFM macrophages, while having no effect on CD206 expression (Figure 7G–K). These results demonstrate that cystine‐mediated glutathionylation inhibits the activation of NF‐κB signaling, thereby inducing the functional reprogramming of macrophages.

### 2.8 High Serum GSH Levels Predicted Poor Therapeutic Responses in NSCLC Patients Underwent PD‐1 Immunotherapy

**Figure 8:**
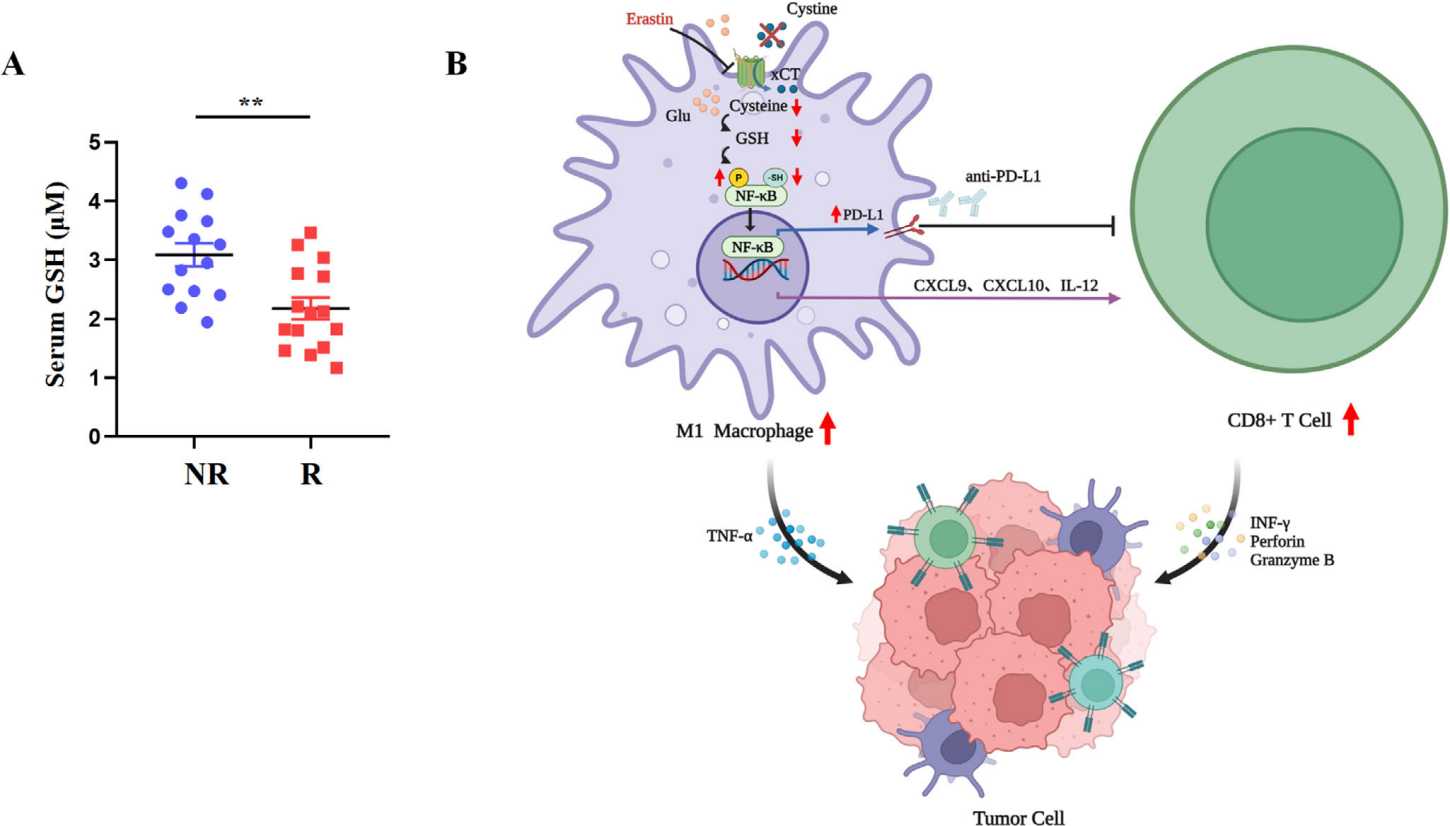
Working model. A) Levels of GSH in the serum of peripheral blood derived from patients with lung cancer before immunotherapy. R represents patients who respond to PD‐1 monoclonal antibody treatment. NR represents patients who are non‐responders to PD‐1 monoclonal antibody treatment (NR = 14, R = 15). Data are shown as mean ± SD. ^**^, *P* < 0.01. B) The schematic diagram illustrates the regulation of macrophage polarization and PD‐L1 expression by cystine deficiency and its synergistic effect with immune checkpoint therapy in inhibiting the progression of lung cancer. Created in BioRender. Xu, Y. (2025) https://BioRender.com/d12mn81.

Given that PD‐1/PD‐L1 blockade immunotherapy is more effective in hosts with restricted cystine availability, we explored the potential association between GSH production and immunotherapy outcomes in NSCLC patients, we collected serum samples from NSCLC patients before receiving anti‐PD‐1 (αPD‐1) therapy. Remarkably, patients who showed clinical benefit from αPD‐1 immunotherapy (responders) had significantly lower serum GSH levels compared to αPD‐1 non‐responders (**Figure** **8**A). This finding indicates that low GSH availability may be essential for achieving optimal therapeutic efficacy in PD‐1 blockade immunotherapy.

Taken together, our study indicates that restricting cystine availability or inhibiting cystine transport reprogrammes macrophages into the anti‐tumor M1‐like phenotype, this effect is further potentiated when combined with PD‐L1/PD‐1 immunotherapy (Figure 8B).

## 3 Discussion and Conclusion

Immunotherapy has fundamentally changed the treatment landscape of lung cancer, highlighting the enormous clinical potential of leveraging the host immune system to recognize and eliminate tumor cells. However, a significant proportion of cancer patients exhibit either intrinsic or acquired resistance to ICI therapy, limiting their efficacy. Accumulating evidence indicates that an immunosuppressive TME is crucial for immune escape and resistance to ICIs.^[^ ^19^ ^]^ TAMs, with high heterogeneity and plasticity, critically affect lung cancer progression and immunotherapy efficacy. The expression and function of TAM can be roughly divided into the classically activated M1 phenotype and the alternatively activated M2 phenotype according to stimulus factors.^[^ ^20^ ^]^ M1‐type TAMs not only possess strong antigen‐presenting capabilities but also produce cytokines such as IL‐12 and TNF‐α, which enhance the cytotoxicity of immune cells against tumor cells.^[^ ^21^ ^]^ Moreover, M1‐type TAMs can secrete chemokines like CXCL9 and CXCL10, promoting the recruitment of Th1 cells and CD8^+^T cells to the tumor microenvironment for activation.^[^ ^22^ ^]^ On the contrary, M2‐type TAMs suppress the proliferation of T cells and NK cells, as well as the expression of cytotoxic effector molecules, by secreting immunosuppressive factors such as Arginase 1, transforming growth factor‐β, and IL‐10.^[^ ^23^, ^24^ ^]^ In most malignant tumors, TAMs typically adopt a tumor‐supportive phenotype, promoting angiogenesis and exerting immunosuppressive functions, thus contributing to poor prognosis.^[^ ^25^ ^]^ Targeting the elimination of the M2 phenotype of TAMs or inducing metabolic reprogramming of the M2 phenotype toward the M1 phenotype are crucial for reversing the immunosuppressive TME.^[^ ^26^ ^]^ Here we demonstrate that cystine deficiency induces macrophage polarization toward the M1 phenotype, increases the secretion of anti‐tumor factors, and upregulates PD‐L1 expression. Targeting cystine utilization along with PD‐L1 antibody therapy enhances anti‐tumor immunity and synergistically suppresses the progression of lung cancer.

The phenotype and function of TAMs are regulated by various amino acid metabolites present in the TME.^[^ ^27^ ^]^ Our experimental results showed that the levels of cystine and cysteine were markedly elevated in the tumor tissues of lung cancer patients compared to the adjacent non‐tumor tissues. Multi‐color flow cytometry analysis of lung cancer patients and the LLC mouse model revealed that xCT is predominantly expressed in tumor cells and macrophages, suggesting that TAMs may exhibit a similar dependency on cystine metabolism as tumor cells. Furthermore, our amino acid metabolomics analysis revealed that macrophages in LLC subcutaneous tumors exhibit elevated levels of cystine. This indicates that the phenotype and function of TAMs could be modulated by cystine.

In cystine‐deficient M1 and M2 macrophages, significant changes were observed in the expression of polarization marker genes, with a marked increase in TNF‐α secretion. RT‐PCR analysis showed a significant upregulation in the transcription levels of CXCL9 and CXCL10 in macrophages under cystine‐deficient conditions. CXCL9 and CXCL10 are critical chemokines that recruit CD8⁺ T cells expressing CXCR3 into tumors, which is crucial for the anti‐tumor immune response mediated by ICIs.^[^ ^16^, ^17^ ^]^ Similarly, our in vivo experiments showed an increase in infiltrating CD8⁺ T cells in tumors of mice fed CFD combined with PD‐L1 antibody.

Our in vivo studies have shown that a CFD alone induces lipid peroxidation in tumor tissues but fails to achieve satisfactory anti‐tumor effects. Therefore, we analyzed the effect of cystine deficiency on the expression of immune checkpoint molecules. We found that restricting cystine utilization upregulated PD‐L1 expression in macrophages both in vitro and in vivo. Glutamine is a crucial amino acid responsible for maintaining GSH levels and regulating PD‐L1 expression in lung and colon cancer cells. Inhibiting glutamine utilization can induce the upregulation of PD‐L1 expression in tumor cells.^[^ ^28^ ^]^ However, we found that cystine deprivation in macrophages strongly induced PD‐L1 expression. This difference may be related to variances in metabolic reprogramming between cancer cells and macrophages, as different cells rely heavily on specific nutrients to adapt to a TME with high oxidative stress.^[^ ^29^, ^30^ ^]^ Proteins related to endogenous cysteine synthesis pathways, such as cystathionine β‐synthase, are highly expressed in tumor cells, and may partially alleviate metabolic stress induced by short‐term external cystine deprivation.^[^ ^31^ ^]^ xCT inhibition upregulates PD‐L1 expression in melanoma cells,^[^ ^32^ ^]^ however, our experimental results showed that cystine deficiency did not significantly upregulate PD‐L1 expression in human lung cancer cell lines. This discrepancy may be due to differences in tumor types and the use of different xCT inhibitors. The specific mechanisms underlying these differences require further investigation.

Mice fed with a cystine‐deficient diet in combination with PD‐L1 antibody immunotherapy showed better anti‐tumor effects. In tumors, inhibiting cystine utilization combined with PD‐L1 immunotherapy increased the mRNA or protein levels of cytotoxic effector molecules, such as IFN‐γ, perforin, and granzyme. Erastin, an inhibitor of cystine transport, promotes ferroptosis in tumor cells. However, the efficacy of using erastin alone in inhibiting tumors is often insignificant in animal experiments. This could be due to the induction of immune escape of tumors by erastin‐mediated increase in tumor cell immunogenicity, leading to upregulation of PD‐L1 expression in TAMs. Previous studies have mainly focused on the role of erastin in inducing ferroptosis in tumor cells,^[^ ^33^ ^]^ overlooking the potential impact of cystine deficiency on the tumor immune microenvironment. Combining PD‐L1 antibodies with erastin or cyst(e)inase can synergistically induce ferroptosis in melanoma cells, effectively enhancing the anti‐tumor effect of PD‐L1 antibodies.^[^ ^34^ ^]^ Our results are consistent with the above findings. In subcutaneous or tail vein‐injected lung metastasis models, the combination of erastin and PD‐L1 monoclonal antibody synergistically inhibited lung cancer growth. After depleting macrophages and then combining with CFD plus PD‐L1 antibodies, the antitumor effect was not significant, indicating that the enhancement of PD‐L1 monoclonal antibodies by the low cystine microenvironment primarily depends on macrophages.

Cystine is converted to cysteine upon entering cells and participates in GSH synthesis. Therefore, we investigated whether decreased GSH synthesis induced by cystine deprivation was the main factor leading to the upregulation of PD‐L1 expression in macrophages. Our experimental results showed that GSH supplementation effectively attenuated the upregulation of M1 macrophage markers and PD‐L1 expression induced by cystine deficiency. GSH depletion results in imbalanced redox homeostasis in cells, inducing ferroptosis in various tumor cells.^[^ ^35^ ^]^ BSO‐induced GSH exhaustion also promotes the upregulation of M1 macrophage markers and the expression of PD‐L1. Furthermore, we investigated whether GSH depletion was related to the upregulation of ROS and the occurrence of ferroptosis along with PD‐L1 expression. When macrophages were treated with GPX4 inhibitors, such as FIN56, RSL3, and FINO2, a significant increase in ROS levels was observed, while PD‐L1 expression remained unchanged. The differential effects of these two types of ferroptosis inducers on PD‐L1 expression may be related to the changes in GSH levels they mediate. Specifically, erastin inhibits cystine transport, leading to a reduction in GSH synthesis. GPX4 inhibitors prevent GPX4 from actively consuming GSH to reduce lipid peroxides, resulting in enhanced ROS levels without a significant decrease in GSH levels.^[^ ^36^, ^37^ ^]^ The ferroptosis inhibitor FER‐1 did not reverse the upregulation of M1 markers and PD‐L1 expression induced by cystine deficiency. The ROS inhibitor NAC attenuated the upregulation of M1 markers and PD‐L1 expression induced by cystine deficiency. This may be because NAC directly scavenges free radicals owing to its active thiol group. It serves as an external source of cysteine that enters the cells and supplements GSH levels,^[^ ^38^ ^]^ thereby attenuating PD‐L1 expression. In summary, cystine deficiency‐mediated GSH depletion does not primarily depend on the upregulation of ROS or ferroptosis to induce PD‐L1 expression.

NF‐κB is a crucial positive regulator of PD‐L1 expression. It can directly induce gene transcription by binding to the PD‐L1 promoter or indirectly regulate the transcription and translation levels of PD‐L1.^[^ ^39^, ^40^ ^]^ Through KEGG enrichment analysis, DEGs were found to be enriched in the NF‐κB signaling pathway. We validated the activation of the NF‐κB signaling pathway in macrophages through immunoblotting experiments. Treatment of macrophages with the NF‐κB‐specific inhibitor, BAY 11–7082, significantly attenuated the upregulation of M1 markers and PD‐L1 expression induced by cystine deficiency. Immunofluorescence experiments revealed that supplementation with GSH partially attenuated the nuclear translocation of p65 under conditions of cystine deficiency. Therefore, cystine deficiency‐mediated GSH depletion primarily relies on activating the NF‐κB signaling pathway to induce M1 markers and PD‐L1 expression.

Protein S‐glutathionylation is a common oxidative‐reductive modification that regulates numerous cellular processes, including cell division, migration, phagocytosis, signal transduction, calcium homeostasis, and energy sensing.^[^ ^41^, ^42^ ^]^ Research has reported that the activity of NF‐κB can be influenced by post‐translational S‐glutathionylation, which occurs reversibly between its cysteine residues and GSH, forming mixed disulfide bonds.^[^ ^43^ ^]^ Decreased levels of GSH in tumor cells inhibit the glutathionylation of SERCA, leading to reduced activity and an increase in cytoplasmic Ca2^+^ levels, which activates NF‐κB signal transduction. Phosphorylated p65 enters the nucleus and drives transcriptional upregulation by binding to the PD‐L1 promoter.^[^ ^28^ ^]^ We further discovered that in macrophages, NF‐κB underwent glutathionylation directly. When we knocked out the glutathionylation enzyme GRX1, NF‐κB remained glutathionylated, leading to reduced nuclear translocation and downstream transcription. The specific modification sites and their role in regulating nuclear translocation warrant further investigation.

Clinical data revealed that patients with lung cancer showing better response to immunotherapy (PR+CR) had relatively lower serum GSH levels before treatment compared to those exhibiting poorer responses (SD+PD).

In conclusion, our findings suggest that inhibiting cystine utilization in macrophages can promote their polarization toward the M1 phenotype, amplifying the secretion of TNF‐α, and enhancing the transcription of CXCL9 and CXCL10. Cystine deficiency results in reduced synthesis of GSH, decreased glutathionylation of NF‐κB, and increased nuclear translocation, consequently activating the NF‐κB signaling pathway and upregulating PD‐L1 expression in macrophages. Mice subjected to a CFD or treated with erastin in combination with PD‐L1 immunotherapy showed enhanced anti‐tumor immunity with synergistic inhibition of lung cancer growth. Therefore, targeting cystine utilization in combination with PD‐L1 immune checkpoint therapy is a potential combination therapy strategy for patients with lung cancer.

## 4 Experimental Section

### Human Specimens

Human peripheral blood samples and lung tissue samples were obtained from patients clinically diagnosed with NSCLC. Blood samples were collected from patients before treatment, and taken from NSCLC patients receiving PD‐1 immunotherapy. PR+CR represents patients with effective treatment responses (R), while PD+SD represents patients with ineffective treatment responses (NR). Cancerous and adjacent non‐cancerous tissues were obtained after surgical procedures. Informed consent was obtained from the patients to collect blood and tissue samples. The study was approved by the Human Research Ethics Committees of Zhejiang University School of Medicine Affiliated Second Hospital and Affiliated Fourth Hospital (Approval numbers: 2020–907, K2022202).

### Animals

Healthy specific pathogen‐free (SPF) male mice, aged 6–8 weeks and weighing between 20 and 22 g, belonging to the C57BL/6J strain, were obtained from Shanghai SLAC Laboratory Animal Co., Ltd. (Shanghai, China). All experimental mice were housed in SPF‐grade animal facilities at Zhejiang University's Experimental Animal Center. All animal experiments were approved by the Zhejiang University Animal Welfare and Ethics Review Committee (Approval numbers: ZJU20230010).

### Cell Lines

A549, PC9, and H2170 lung cancer cell lines were obtained from the American Type Culture Collection, ATCC, USA. These cell lines were routinely passaged and cultured in RPMI‐1640 complete medium or cystine‐deficient medium. The mouse Lewis lung cancer cell line (LLC) with luciferase marker was purchased from PerkinElmer in Waltham, USA. Primary peritoneal macrophages were isolated and extracted from C57BL/6J mice. These cells were grown in Dulbecco's Modified Eagle Medium (DMEM) with either complete or cystine‐deficient medium (Keygen, Nanjing, China). Both media were supplemented with 10% fetal bovine serum (FBS) and 1% penicillin‐streptomycin. Cystine‐deficient medium exclusively lacks L‐Cystine 2HCl, while all other amino acids were supplemented at standard concentrations. All cells were cultured in a humidified atmosphere with 5% CO_2_ at 37 °C in a cell culture incubator.

### Extraction of Mouse Peritoneal Macrophages

1 mL of sterile 3% thioglycolate broth was injected into the peritoneal cavity of 6‐ to 8‐week‐old C57BL/6J mice. The mice were sacrificed after three days. The peritoneal cavity of the mice was rinsed with DMEM medium. Cells were incubated at 37 °C for 6 h. After incubation, the supernatant was discarded, and purified adherent macrophages derived from the peritoneal cavity of mice were obtained.

### Real‐Time Quantitative PCR

RNA was extracted from each sample using the TRIzol reagent following the manufacturer's protocol (Pufei Biology, Shanghai, China). cDNA was synthesized by reverse transcription using the ReverTra Ace Kit (Toyobo, Osaka, Japan). RT‐PCR was performed using SYBR RT‐PCR Master Mix (Vazyme, Nanjing, China) on the CFX‐Touch 96 fluorescent quantitative PCR System. The primer sequences used in this study are listed in Table S1 (Supporting Information).

### Enzyme‐Linked Immunosorbent Assay (ELISA)

Cytokine levels in the supernatant of cell culture and mouse peripheral blood were measured by ELISA. All operations were conducted according to the manufacturer's protocol. Specific antibodies for the following proteins were utilized: Anti‐mouse IL‐1β (Invitrogen, Carlsbad, CA, USA); Anti‐mouse IL‐10 (Invitrogen, Carlsbad, CA, USA); Anti‐mouse IL‐12 (Invitrogen, Carlsbad, CA, USA); Anti‐mouse TNF‐α (Invitrogen, Carlsbad, CA, USA); Anti‐mouse CXCL9 (Multi Sciences, Hangzhou, China); Anti‐mouse CXCL10 (Abclonal, Wuhan, China). Samples in a 96‐well microplate were measured in duplicates at 450 and 570 nm absorbance to determine the optical densities (OD) by a microplate reader.

### Immunofluorescence Staining

Macrophages were seeded on sterile small round slides in a 24‐well plate. After stimulation, cells were fixed with paraformaldehyde and permeabilized with Triton X‐100. Cells were incubated with anti‐p65 (CST, BOS, USA) and anti‐PD‐L1 (Proteintech, CHI, USA) antibodies at 4 °C overnight, and incubation with a fluorophore‐conjugated secondary antibody for 1 h in a dark, humidified chamber. After washing, cell nuclei were stained with DAPI (Beyotime, Shanghai, China) and imaged using an Olympus FV3000 confocal laser microscope (Olympus, Tokyo, Japan).

### Detection of Cell Activity and Cytotoxicity by Calcein AM/PI

Macrophages were seeded in confocal culture dishes. After stimulation, 1000 × Calcein AM/PI dye was diluted (Beyotime, Shanghai, China), and the working solution was prepared, added to the macrophages in the confocal petri dish, and incubated at 37 °C for 30 min away from light and examined under a fluorescence microscope (Olympus, Tokyo, Japan).

### Immunoblotting Analysis

After stimulation, cells were lysed in radioimmunoprecipitation assay (RIPA) buffer containing protease and phosphatase inhibitors (Beyotime, Shanghai, China). The extracted proteins were separated by sodium dodecyl sulfate‐polyacrylamide gel electrophoresis (SDS‐PAGE) after quantification using a BCA Protein Assay Kit (Beyotime, Shanghai, China). Proteins were transferred onto polyvinylidene fluoride (PVDF) membranes. The following antibodies against the proteins of interest were used in this study are listed in Table S2 (Supporting Information).

### In Vivo Metastatic Assay

LLC lung cancer cells labeled with the luciferase marker were injected into the tail vein of C57BL/6J mice. Specifically, 1.5 × 10^6^ tumor cells were injected per mouse. After 11 days, the mice were randomly divided into four treatment groups: control, imidazole ketone erastin (IKE) (TargetMol, MA, USA), anti‐PD‐L1 (αPD‐L1), and IKE+αPD‐L1 combined. After grouping, each mouse was administered an intraperitoneal injection of 30 mg kg^−1^ IKE or 100 µg αPD‐L1 alone or in combination every three days; the same volume of 5% DMSO+95% corn oil and IgG2b isotype antibody was administered to the control mice. After the fifth administration, mice were anesthetized with 1% sodium pentobarbital solution, followed by an intraperitoneal injection of 2 mg luciferin/mouse. Images were captured using the IVIS Spectrum Small Animal Live Imaging System (PerkinElmer, MA, USA). A live imaging software (PerkinElmer, MA, USA) was used to analyze the fluorescence imaging intensity of the lung region.

### Subcutaneous Tumor Model

LLC lung cancer cells (1 × 10^6^ cells per mouse) were subcutaneously injected into the lateral abdomen of C57BL/6J mice. When the tumor was palpable, the mice were randomly divided into the following groups: normal control diet (NCD) group, cystine‐free diet (CFD) group, αPD‐L1 treatment group, and CFD+αPD‐L1 treatment group, and the frequency of administration was once every 3 days. Otherwise, the animals were divided into a DMSO+IgG group, IKE+IgG group, DMSO+αPD‐L1 group, or IKE+αPD‐L1 combined treatment group, and intraperitoneal injection was administered once every three days. In the macrophage depletion experiment, 100 µl of clodronate liposomes (FormuMax, CA, USA) was injected into the mice's peritoneal cavity twice a week.The length and width of the maximum diameter of the subcutaneous tumor were measured, and the volume was calculated as follows: Tumor volume (mm^3^) = length (mm) × width^2^ (mm^2^) /2. When the unilateral diameter of the tumor exceeded 20 mm, or the volume of the tumor was greater than 2000 mm^3^, the tumor volume was considered to have reached the “terminal stage”, after which the mice were sacrificed.

### Flow Cytometry Analysis

A single‐cell suspension obtained from cell lines or tissue dissociation was stained with different fluorescein dyes conjugated to antibodies at 4 °C for 30 min. Using Fixable Viability Dye eFluor staining (eBioscience, San Diego, CA, USA), the effect of dead cells on experimental results was excluded. Samples were subjected to analysis on an ACEA Novocyte flow cytometer and analyzed using the FlowJo software (Tree Star). The following combination of flow cytometry antibody markers were listed in Table S3 (Supporting Information).

### Magnetic Bead Sorting

Single‐cell suspensions were prepared from subcutaneous LLC tumors. Macrophages and NK cells were isolated using F4/80‐ and CD49b‐conjugated magnetic beads (Cat# 100–0659, Cat# 18 755), respectively, following the manufacturer's protocol (Stemcell, Vancouver, Canada). The samples were analyzed using an ACEA Novocyte flow cytometer and processed with FlowJo software (Tree Star).

### siRNA Interference

Briefly, 100 µl of Opti‐MEM and 5 µl of transits‐TKO interference reagent were mixed thoroughly. The interference sequence siRNA corresponding to the target gene (50 nM) was added and mixed (GenePharma, Suzhou, China). The mixture was incubated at 26 °C for 15 min. The mixture was added to macrophages, and proteins were detected 48 h later to verify the interference effect. The interference sequences were listed in Table S4 (Supporting Information).

### Reactive Oxygen Species (ROS), Glutathione (GSH) and Malondialdehyde (MDA) Detection

Primary peritoneal macrophages of mice were treated with drugs and incubated with a DCFH‐DA (10 mm) probe at 37 °C for 20 min. The mean fluorescence intensity of FITC channels was measured by ACEANovocyte flow cytometry, representing the intracellular ROS levels (Beyotime, Shanghai, China). The supernatant of the macrophages was collected to determine GSH levels following the steps outlined in the GSH content detection kit (Solarbio, Beijing, China) and MDA content detection kit (NanJing Jiancheng, Nanjing, China). The OD values at 412 and 532 nm were measured on the microplate absorbance reader, and the relative GSH and MDA contents were calculated.

### Detection of Reactive L‐cysteine Levels in Macrophages

Collect peritoneal macrophages treated with CCM, CFM, and CFM+GSH for 24 h. The cells were disrupted through mechanical homogenization, followed by centrifugation at 4 °C and 10 000 × g for 10 min. Add the reagents to the collected supernatant sequentially, following the instructions provided in the Cysteine Colorimetric Assay Kit (Elabscience, Wuhan, China). Incubate at room temperature for 10 min. The OD values at 600 nm were measured using a microplate absorbance reader, and the relative L‐cysteine levels were calculated.

### Metabolomics Analysis

Fresh lung cancer tissues and adjacent non‐cancerous tissues were weighed, and a 500 µl pre‐cooled 70% methanol‐water solution was added. The mixture was vortexed for 2 min, centrifuged at 12 000 × g at 4 °C for 10 min, and the supernatant was obtained. The supernatant was then incubated at −20 °C for 30 min and centrifuged at 12 000 rpm at 4 °C for 10 min. Subsequently, 200 µl of the centrifuged supernatant was detected by liquid chromatography‐tandem mass spectrometry (PTM BIO, Hangzhou, China). MultiQuant 3.0.3 software was used to identify and annotate the detected metabolites, and quantitative analysis and calculations were performed. The metabolite content data were normalized by unit variance scaling, and a principal component analysis (PCA) was performed using the built‐in statistical prcomp function in the R software. Differential metabolites were screened using |log2(fold change)| ≥ 1 and *P* < 0.05 as the threshold for a significant difference.

### RNA Sequencing

Cystine‐free medium (CFM) or complete culture medium (CCM)‐cultured macrophages were homogenized in TRIzol on ice. Cell RNA was subjected to mRNA sequencing utilizing next‐generation sequencing (NGS) by Biomarker Technologies Co., Ltd. (Beijing, China). The Limma (Version 3.16) software package was used to analyze differentially expressed genes in CFM or CCM‐cultured macrophages. |log2 (fold change)| ≥ 1 and *P* < 0.05 were set as the screening threshold for significant differences. Differential gene volcano maps were plotted using the ggplot2 (Version 3.4.1) software package. Immune checkpoint genes were labeled using the ggrepel (Version 0.9.1) software package. Heat maps were plotted using the pheatmap (Version 1.0.12) software package.

### Kyoto Encyclopedia of Genes and Genomes (KEGG) Analysis and Gene Set Enrichment Analysis (GSEA)

All differentially expressed genes were sorted from largest to smallest according to |log2 (fold change)|. KEGG analysis was performed using the clusterProfiler (Version 4.6.0) R package, and GSEA was performed using the enrichplot (Version 1.18.3) R package to visualize the enrichment of the NF‐κB pathway.

### Statistical Analysis

Unless otherwise stated, all data were expressed as mean ± SD. The normality of data distribution was analyzed using the Shapiro–Wilk test. Data between the two groups were compared using the double‐tailed unpaired Student's *t*‐test. One‐way analysis of variance (ANOVA) and log‐rank test were subjected to Tukey's test. *P* < 0.05 indicated a statistically significant difference. All data were analyzed using GraphPad Prism 8.

### Institutional Review Board Statement

The study was approved by the Human Research Ethics Committees of Zhejiang University School of Medicine Affiliated Second Hospital and Affiliated Fourth Hospital (Approval numbers: 2020–907, K2022202). All animal experiments were approved by the committee of Zhejiang University School of Medicine (Approval numbers: ZJU20230010).

## Conflict of Interest

The authors declare no conflict of interest.

## Author Contributions

Y.X., S.L., J.W., and S.X. contributed equally to this work. X.Y., P.X., and K.W. designed the study. S.M.L., S.M.X., M.S., C.Y.W., and J.J.W. provided the databases. J.H.Y., Z.Y.X., Y.L.S., N.N.L., and Y.K.Y. assembled and analyzed the data. L.A., J.N.Z., Y.Y., P.Y., J.C., J.H.S., and M.S.L. wrote the manuscript. The final submitted version has been approved by all authors.

## Supporting information



Supporting Information

Supporting Information

Supporting Information

Supporting Information

Supporting Information

Supporting Information

Supporting Information

Supporting Information

Supporting Information

Supporting Information

Supporting Information

Supporting Information

Supporting Information

Supporting Information

Supporting Information

Supporting Information

Supporting Information

Supporting Information

Supporting Information

## Data Availability

The data that support the findings of this study are openly available in NCBI at https://www.ncbi.nlm.nih.gov/bioproject/?term=PRJNA1144237, reference number 1144237.
