# Supplementary material for: Targeting Cystine Metabolism in the Lung Cancer Environment Enhances the Efficacy of Immune Checkpoint Inhibition
Source: Adv Sci (Weinh). 2025 Jul 10;12(35):e13084. doi: 10.1002/advs.202413084 (PMC12463131; doi:10.1002/advs.202413084)
Supplement: Supplementary file 1 — Supporting Information [file ADVS-12-e13084-s011.docx]

**Targeting cystine metabolism in the** **lung cancer environment enhances the efficacy of immune checkpoint inhibition**

**Supplementary Figure legends**

Figure S1. Gating strategy for different cell types within tumor tissues.

(A-B) Gating strategy diagram for single-cell suspensions prepared from lung cancer patient tissues and subcutaneous LLC tumors in mice for flow cytometric analysis.

Figure S2. Purity of magnetic bead-sorted macrophages and NK cells in subcutaneous tumor tissues from LLC mice.

(A-B) Single-cell suspensions were prepared from mouse tumor tissues. Macrophages and NK cells were isolated using F4/80 and CD49b magnetic beads, respectively, and the purity of the sorted cells was analyzed using flow cytometry.

Figure S3. Effect of cystine on macrophage activity.

1. Purity of macrophages as measured by the expression of CD11b and F4/80 (n=6/group). (B) CCK8 assay was performed to determine the viability of macrophages cultured for different durations in CFM (n=5/group). (C) After 24 hours of culture in CCM or CFM, immunofluorescence images of live cells stained with Calcein AM and dead cells stained with PI were obtained. Scale bar = 50 μm. Data are representative of three independent experiments. Data are presented as mean±SD. NS, Not Significant.

Figure S4. Cystine deficiency induces mouse peritoneal macrophages and human monocytes to polarize towards M1 phenotype.

(A) RT-PCR analysis was conducted to assess the relative expression of polarization markers, including TNF-α, CXCL9, CXCL10, IL-12a, IL-12b and CD206 in mouse peritoneal macrophages after culturing in DMSO or erastin for 8 hours (n=3/group). (B) RT-PCR analysis was conducted to assess the relative expression of polarization markers, including TNF-α, CXCL9, and CXCL10 in THP-1 after culturing in CCM or CFM for 24 hours (n=3/group). Data are representative of three independent experiments. Data are presented as mean±SD. NS, Not Significant; **, *P* < 0.01; ***, *P* < 0.001.

Figure S5. Cystine deprivation promotes M1 polarization in macrophages co-cultured with tumor cells.

(A-B) The histograms and statistical graphs show the membrane surface levels of CD86 and CD206 on peritoneal macrophages, analyzed by flow cytometry after 24 hours of co-culture with LLC cells at different ratios in CCM or CFM (n=3/group). (C) RT-PCR analysis was conducted to assess the relative expression of polarization markers, including TNF-α, and CXCL10 in peritoneal macrophages after 24 hours of co-culture with LLC cells at different ratios in CCM or CFM (n=3/group). Data are representative of three independent experiments. Data are presented as mean±SD. NS, Not Significant; *, *P* < 0.05; ***, *P* < 0.001.

Figure S6. Cystine deficiency induces repolarization of M2-type macrophages toward the M1-type.

1. Histograms and statistical graphs show the levels of CD86 and CD206 proteins on the cell membrane surface of M1 and M2 macrophages after culturing in CCM or CFM for 24 hours, as analyzed by flow cytometry (n=5/group). (B) ELISA was performed to detect changes in the levels of TNF-α, IL-12, IFN-γ, IL-1β, IL-10, and TGF-β in the supernatant of M1 and M2 type macrophages after culturing in CCM or CFM for 24 hours (n=3/group). Data are representative of three independent experiments. Data are presented as mean±SD. NS, Not Significant; *, *P* < 0.05; **, *P* < 0.01; ***, *P* < 0.001.

Figure S7 Cystine restriction does not influence PD-L1 expression in human lung cancer cells.

(A-C) Histograms and statistical charts depict PD-L1 protein levels in lung cancer cells (A549, PC9, and H2170) after culturing in CFM or treatment with erastin (n=3/group). Data are representative of three independent experiments. Data are presented as mean±SD. NS, Not Significant. **, *P* < 0.01.

Figure S8. Serological parameters and splenic immune cell counts in mice following cystine combined with PD-L1 antibody therapy.

1. Biochemical indicators in mouse blood treated with NCD, CFD, αPD-L1, and CFD+αPD-L1(n=6/group). ALB, albumin; BUN, blood urea nitrogen; CRE, creatinine; TCHO, total cholesterol; CPK creatine phosphokinase; GOT, glutamic oxalacetic transaminase; GPT, glutamic-pyruvic transaminase. (B) Proportions of different immune cell subpopulations infiltrating in spleen (n=6/group). Data are shown as mean ± SD. NS, Not Significant.

Figure S9. The increased PD-L1 expression and M1 polarization in macrophages during cystine deficiency are linked to reduced GSH levels.

1. Relative GSH levels in mouse peritoneal macrophages treated with DMSO or 20 μM erastin for 24 hours (n=3/group) . (B) RT-PCR analysis was conducted to measure the relative expression levels of TNF-α and CXCL10 in peritoneal macrophages treated with DMSO, erastin, and erastin+GSH for 24 hours (n=3/group). (C) After treating peritoneal macrophages with DMSO, erastin, and erastin+GSH for 24 hours, RT-PCR was used to detect the mRNA levels of PD-L1 (n=3/group) .(D) After treating peritoneal macrophages with DMSO, erastin, and erastin+GSH (5 mM) for 24 hours, and flow cytometry was used to detect PD-L1 protein expression (n=3/group) . (E) RT-PCR analysis was conducted to measure the relative expression levels of TNF-α and CXCL10 in peritoneal macrophages treated with DMSO, erastin, and erastin+NAC for 24 hours (n=3/group). (F) After treating peritoneal macrophages with DMSO, erastin, and erastin+NAC for 24 hours, RT-PCR was used to detect the mRNA levels of PD-L1 (n=3/group) . (G) After treating peritoneal macrophages with DMSO, erastin, and erastin+NAC (1 mM) for 24 hours, and flow cytometry was used to detect PD-L1 protein expression (n=3/group) . Data are representative of three independent experiments. Data are presented as mean±SD. NS, Not Significant; *, *P* < 0.05; **, *P* < 0.01; ***, *P* < 0.001.

Figure S10. Cystine deficiency upregulates PD-L1 expression and M1 polarization in a ferroptosis-independent manner.

1. Peritoneal macrophages were treated with erastin (20 μM), FIN56 (2 μM), RSL3 (0.5 μM), or FINO2 (5 μM) for 12 hours. Subsequently, they were stained with the ROS probe DCFH-DA to detect intracellular ROS levels (n=3/group). (B) Relative GSH levels in peritoneal macrophages after treatment with different ferroptosis inducers for 24 hours (n=3/group). (C) Peritoneal macrophages were treated with erastin, FIN56, RSL3, or FINO2 for 24 hours, and flow cytometry was used to detect PD-L1 protein expression (n=3/group). (D-E) Peritoneal macrophages were treated with CCM, CFM, and CFM+FER-1 or DMSO, erastin, and erastin+FER-1 for 24 hours. Subsequently, RT-PCR was used to detect the mRNA levels of PD-L1 (n=3/group). (F-G) Peritoneal macrophages were treated with CCM, CFM, and CFM+FER-1 or DMSO, erastin, and erastin+FER-1 for 24 hours. Flow cytometry was used to detect the levels of PD-L1 (n=3/group). (H) RT-PCR analysis was conducted to measure the relative expression levels of TNF-α, CXCL9, and CXCL10 in peritoneal macrophages treated with CCM, CFM, or CFM+FER1 for 24 hours (n=3/group). (I) ELISA was used to detect changes in the levels of TNF-α, CXCL9, and CXCL10 in the supernatant of macrophages treated with CCM, CFM, or CFM+FER1 for 24 hours (n=3/group). Data are shown as mean ± SD. NS, Not Significant; *, *P* < 0.05; **, *P* < 0.01; ***, *P* < 0.001.

Figure S11. Cystine deficiency does not affect other signaling pathways regulating PD-L1 expression.

1. C) Levels of phosphorylated p65 and phosphorylated IKK in A549, PC9, and H2170 cells treated with CFM or 20 μM erastin for 0, 1, 3, 6, 12, and 24 hours were detected by immunoblotting. (D-F) Levels of phosphorylated p65 and phosphorylated IKK in macrophages treated with RSL3, FIN56, or FINO2 for 0, 1, 3, 6, 12, and 24 hours were detected by immunoblotting. Data are representative of three independent experiments.

Figure S12. BSO-induced GSH depletion drives concurrent PD-L1 elevation and M1 polarization in macrophages.

(A-C) After treating peritoneal macrophages with DMSO, BSO, BSO+NAC and BSO+BAY 11-7082 for 24 hours, flow cytometry was employed to measure the levels of CD80, CD86, and PD-L1 (n=3/group). (D) An immunofluorescence assay was used to detect the expression and distribution of p65 in macrophages treated with DMSO and BSO for 3 hours. Scale bar = 50 μm. (E-H) Levels of phosphorylated p65 and phosphorylated IKK in peritoneal macrophages, A549, PC9, and H2170 cells treated with DMSO or 400 μM BSO for 0, 1, 3, 6, 12, and 24 hours were detected by immunoblotting. Data are representative of three independent experiments. Data are presented as mean±SD. *, *P* < 0.05; **, *P* < 0.01; ***, *P* < 0.001.

Figure S13. The knockout efficiency of GRX1 in macrophages.

(A-B) RT-PCR and immunoblottingt were performed to detect the knockout efficiency of GRX1 in macrophages (n=3/group). Data are representative of three independent experiments. Data are presented as mean±SD. ***, *P* < 0.001.
