# Supplementary figures and images for: Targeting Cystine Metabolism in the Lung Cancer Environment Enhances the Efficacy of Immune Checkpoint Inhibition
Source: Adv Sci (Weinh). 2025 Jul 10;12(35):e13084. doi: 10.1002/advs.202413084 (PMC12463131; doi:10.1002/advs.202413084)

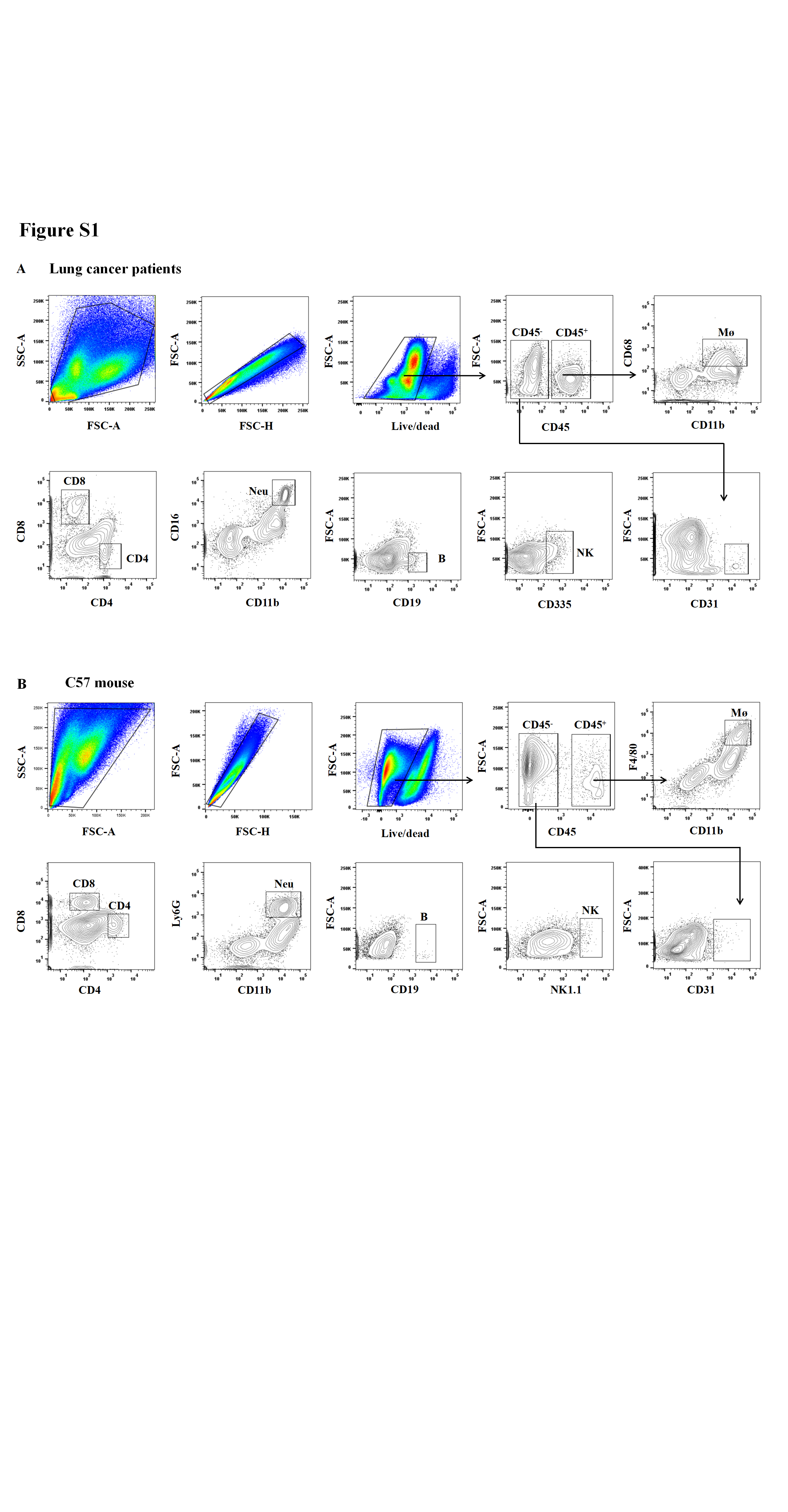

Supplement: Supplementary file 2 — Supporting Information [file ADVS-12-e13084-s007.tiff]

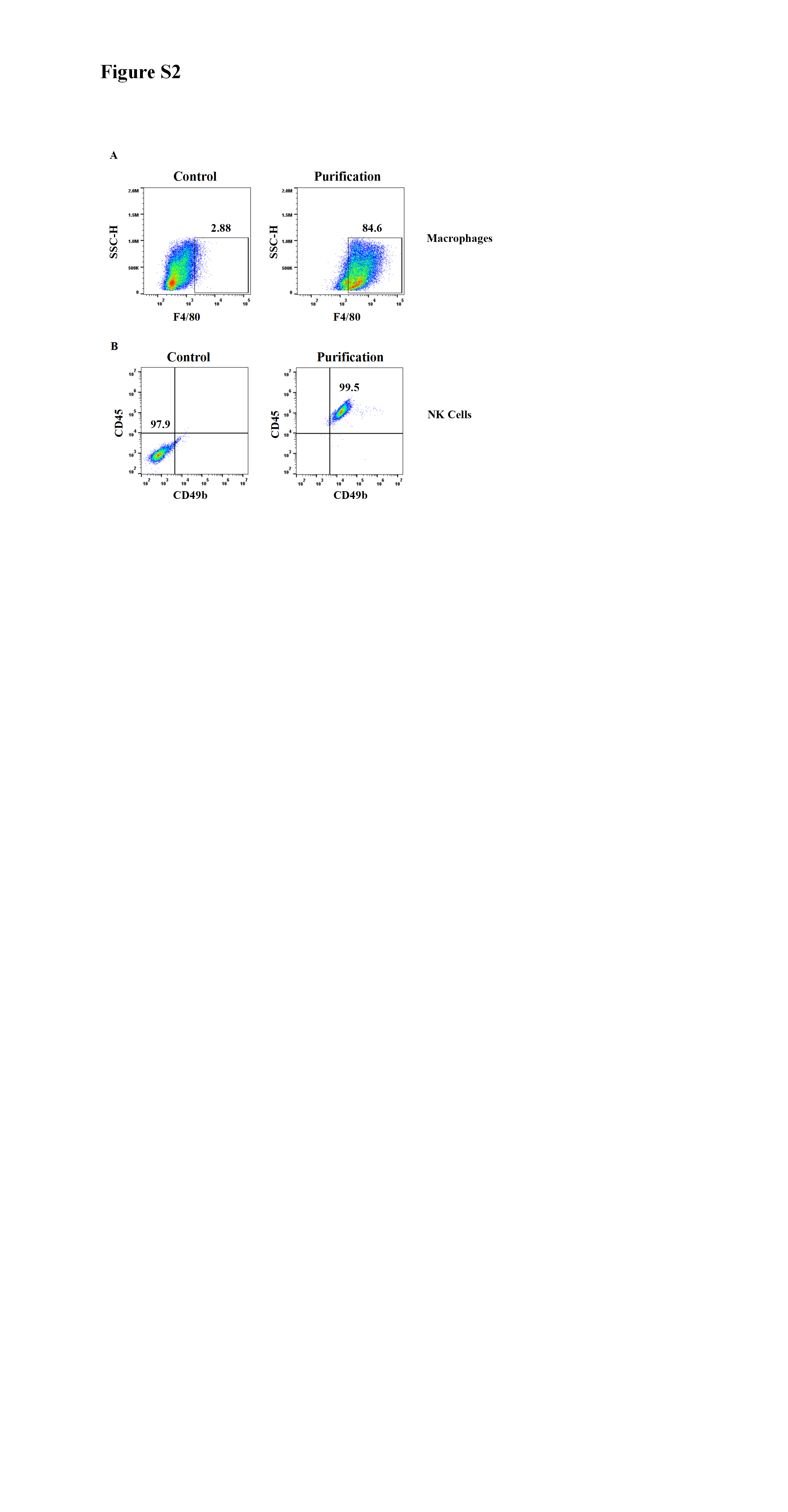

Supplement: Supplementary file 3 — Supporting Information [file ADVS-12-e13084-s002.tiff]

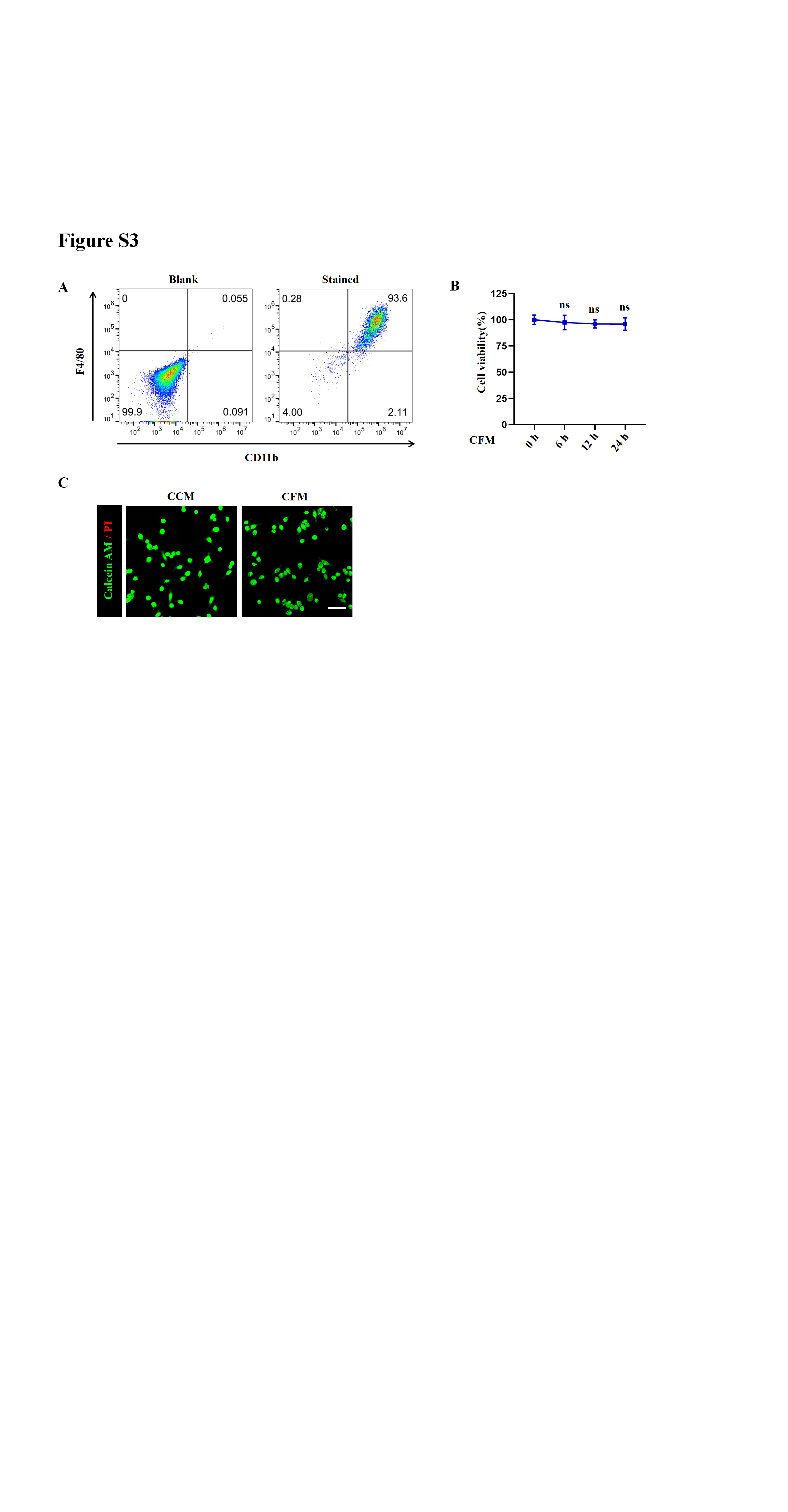

Supplement: Supplementary file 4 — Supporting Information [file ADVS-12-e13084-s009.tiff]

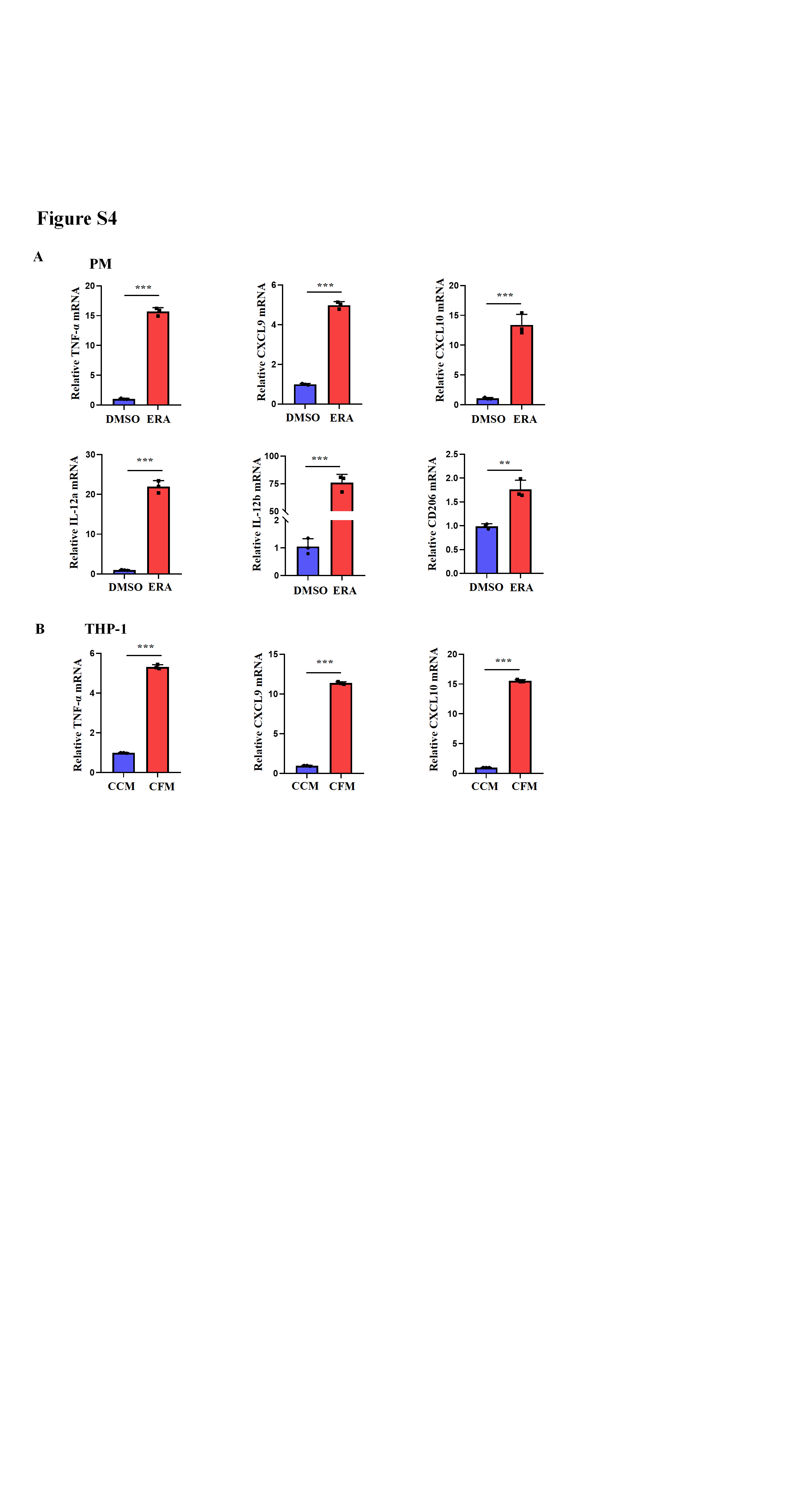

Supplement: Supplementary file 5 — Supporting Information [file ADVS-12-e13084-s019.tiff]

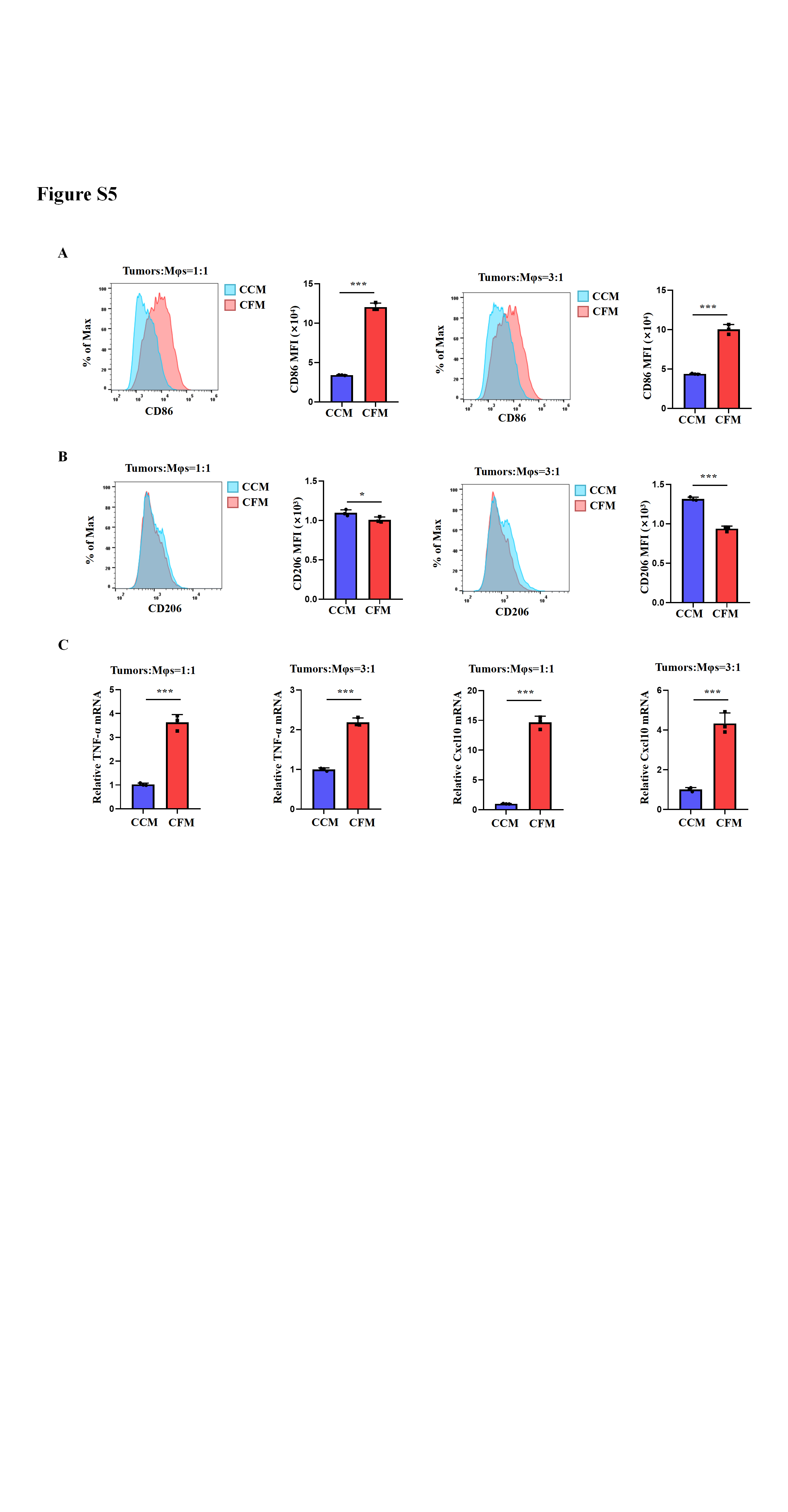

Supplement: Supplementary file 6 — Supporting Information [file ADVS-12-e13084-s004.tiff]

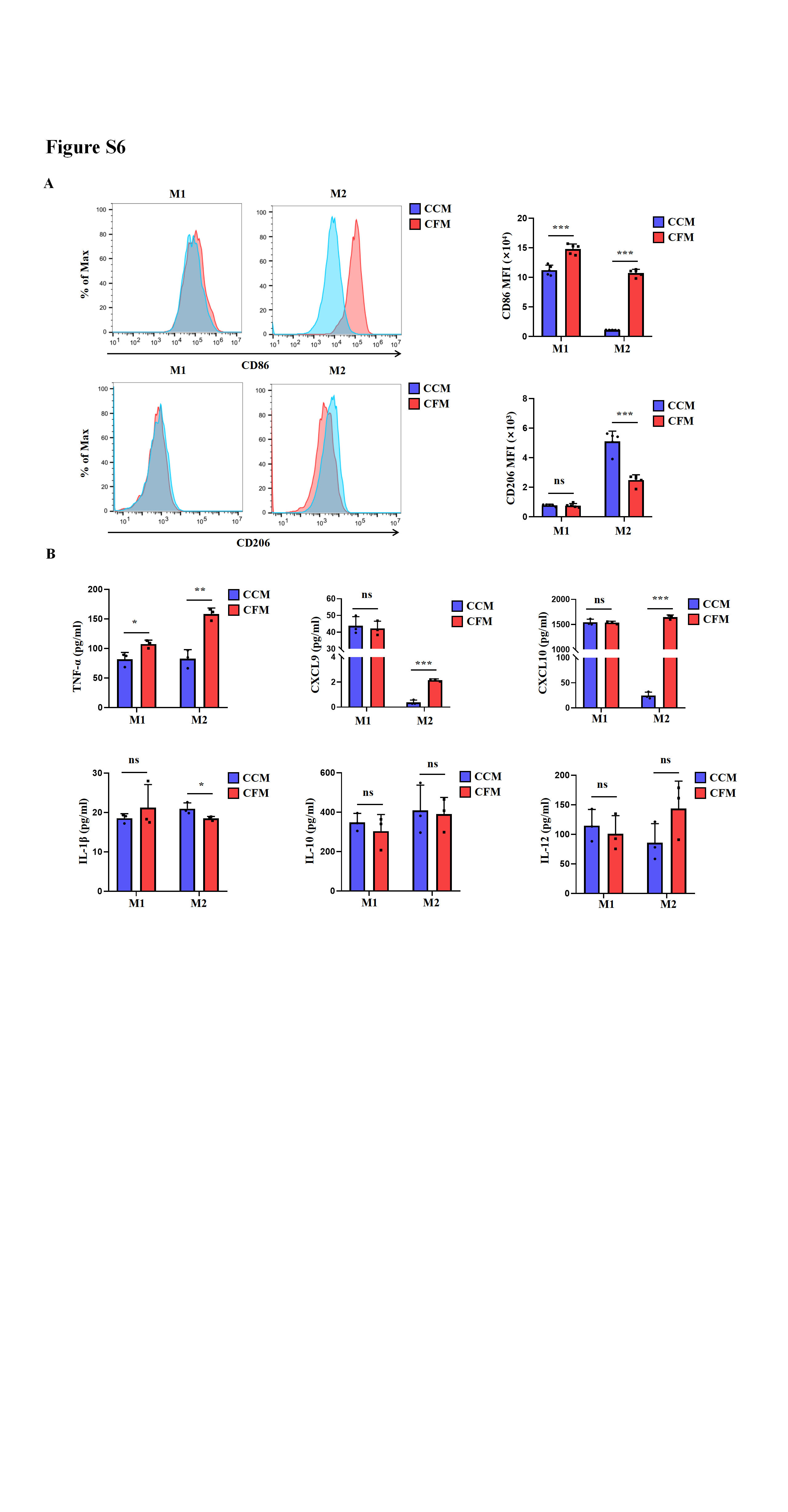

Supplement: Supplementary file 7 — Supporting Information [file ADVS-12-e13084-s003.tiff]

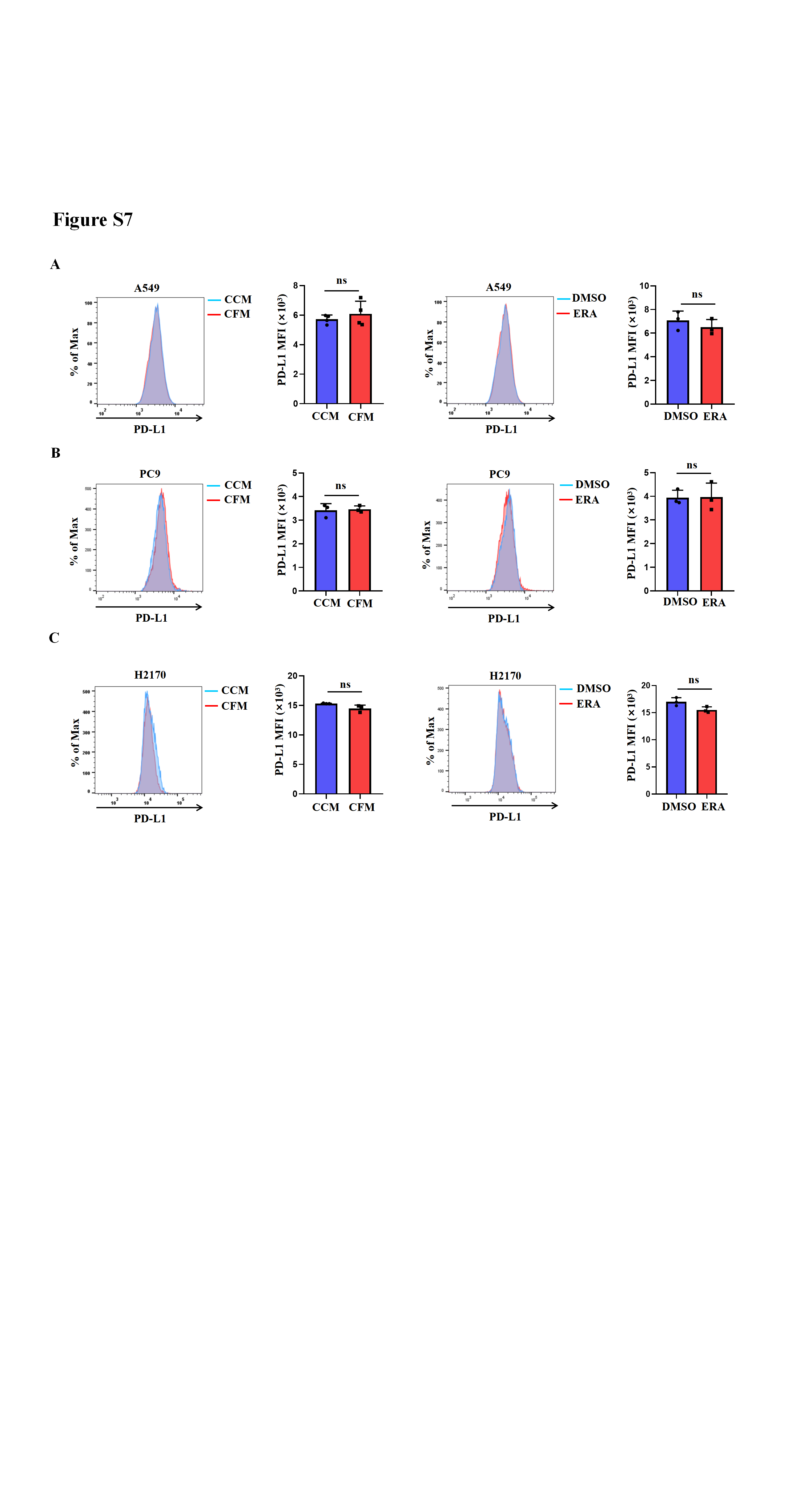

Supplement: Supplementary file 8 — Supporting Information [file ADVS-12-e13084-s015.tiff]

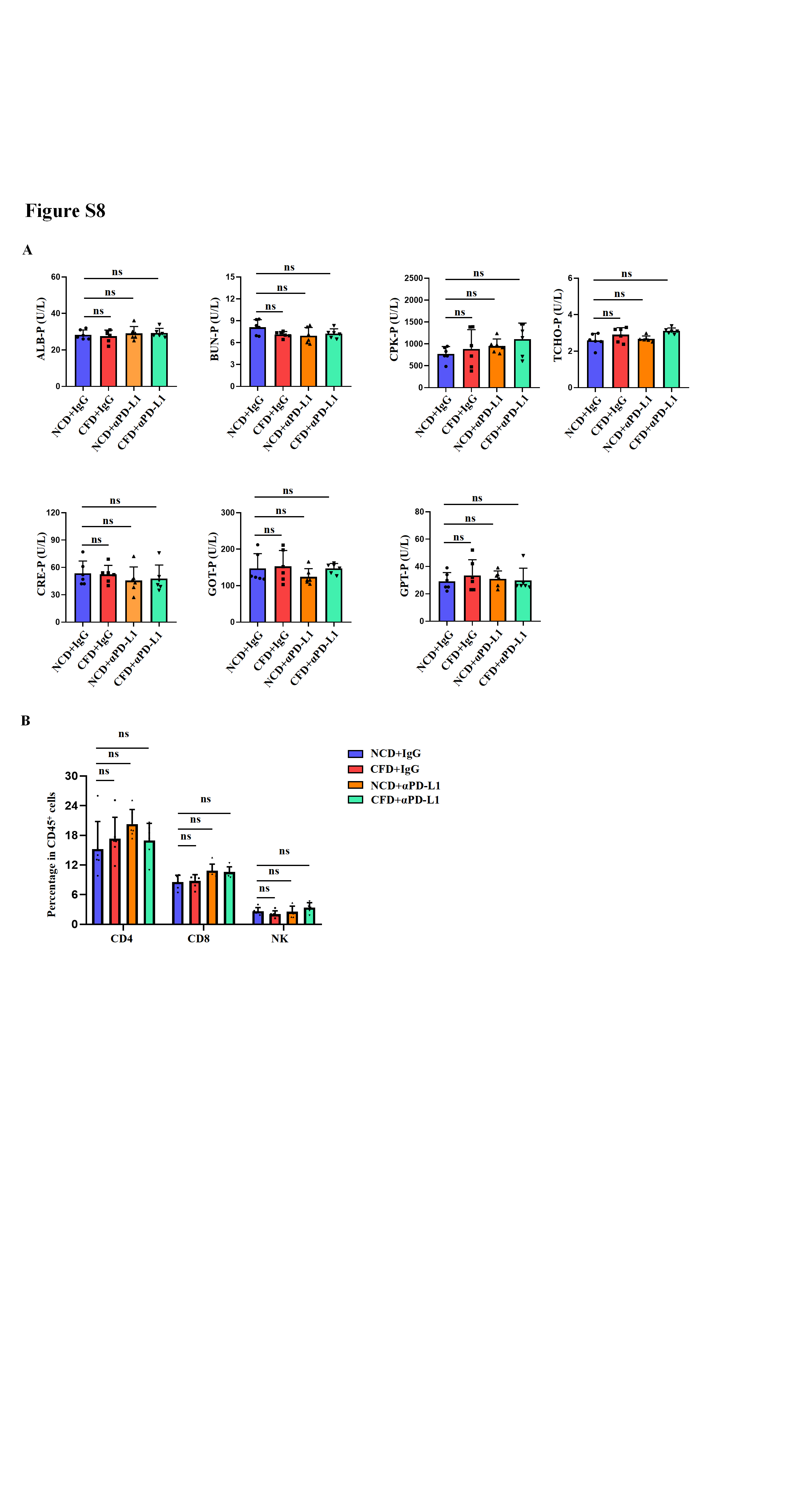

Supplement: Supplementary file 9 — Supporting Information [file ADVS-12-e13084-s017.tiff]

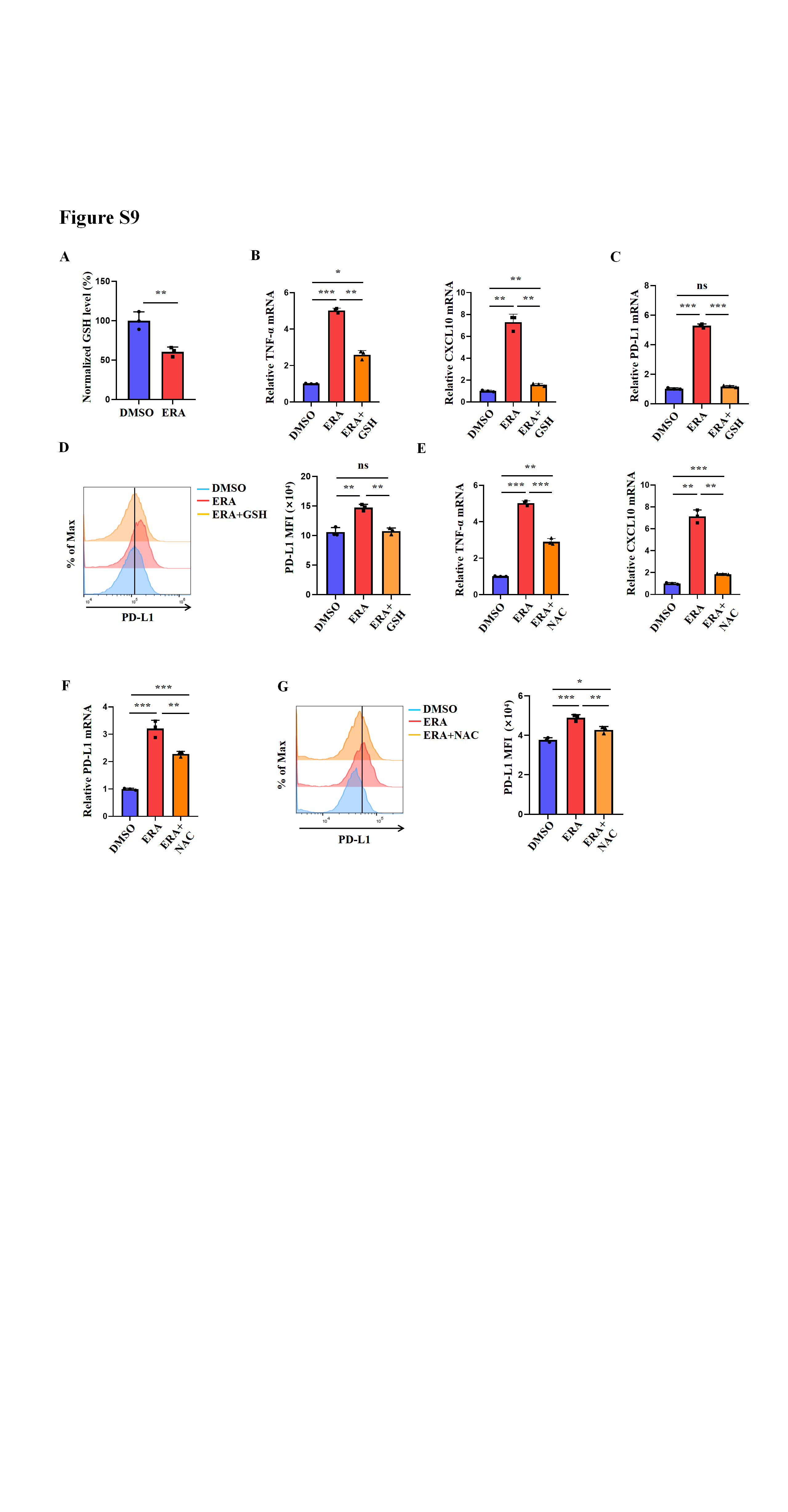

Supplement: Supplementary file 10 — Supporting Information [file ADVS-12-e13084-s018.tiff]

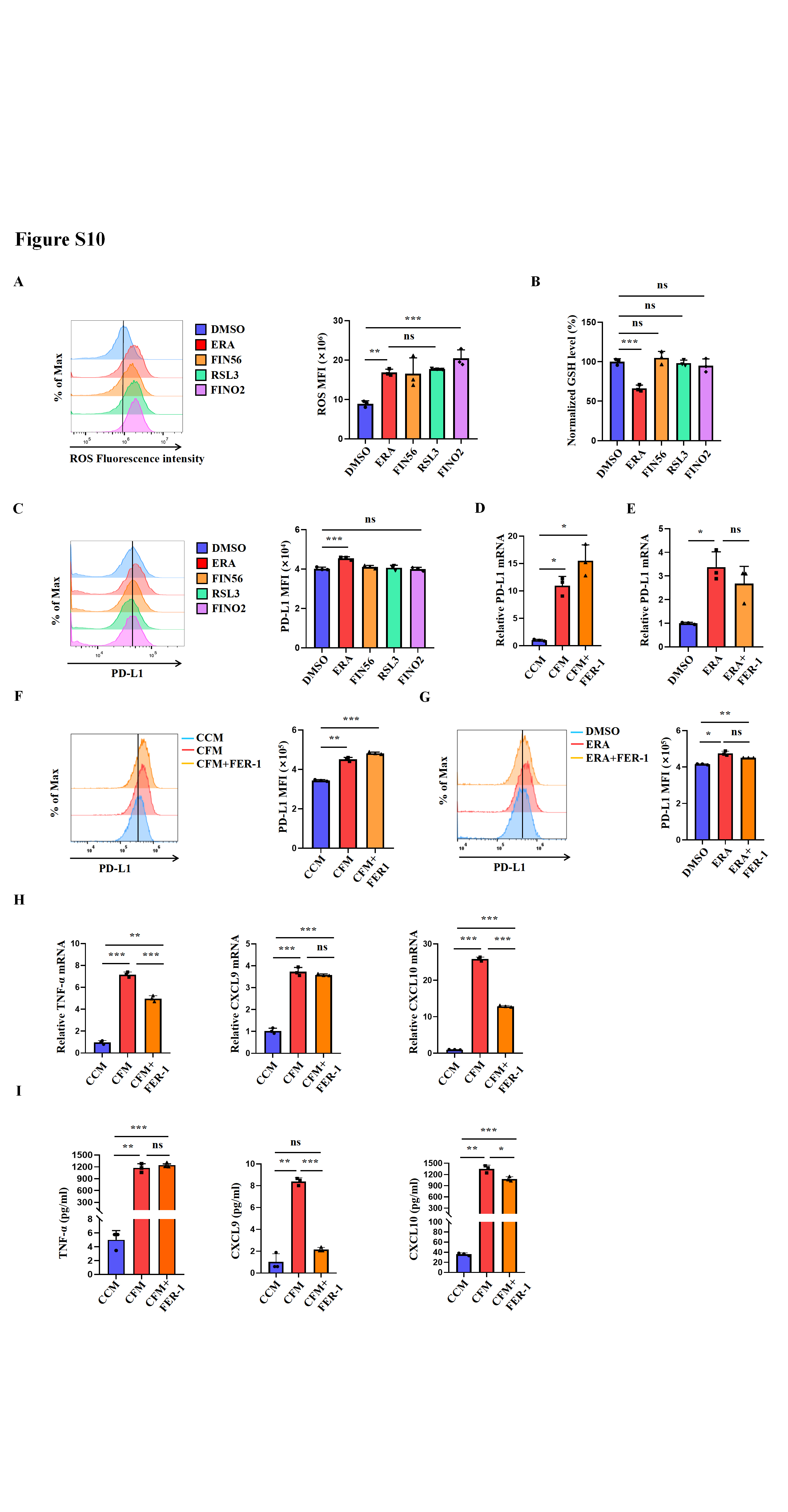

Supplement: Supplementary file 11 — Supporting Information [file ADVS-12-e13084-s016.tiff]

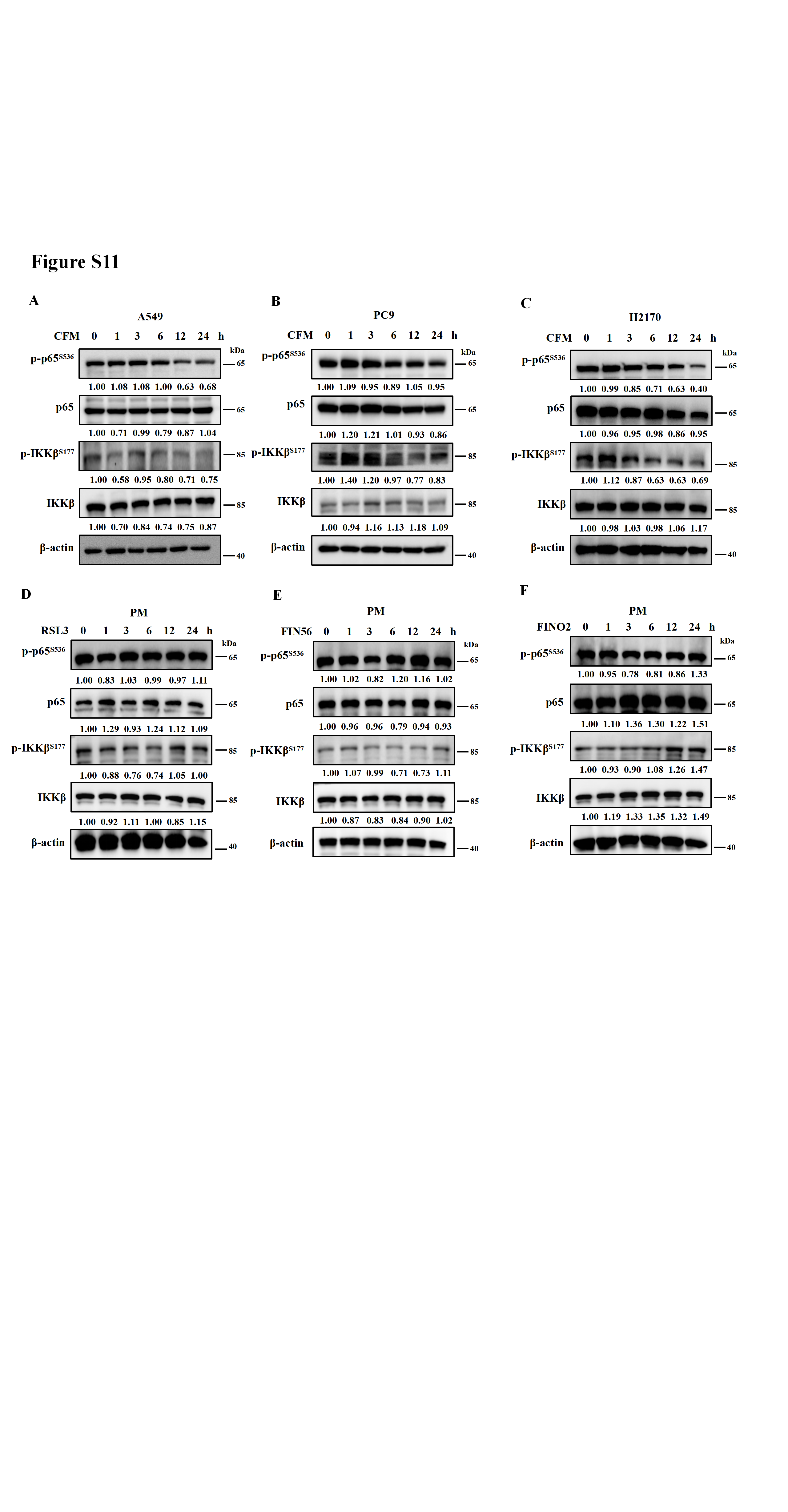

Supplement: Supplementary file 12 — Supporting Information [file ADVS-12-e13084-s008.tiff]

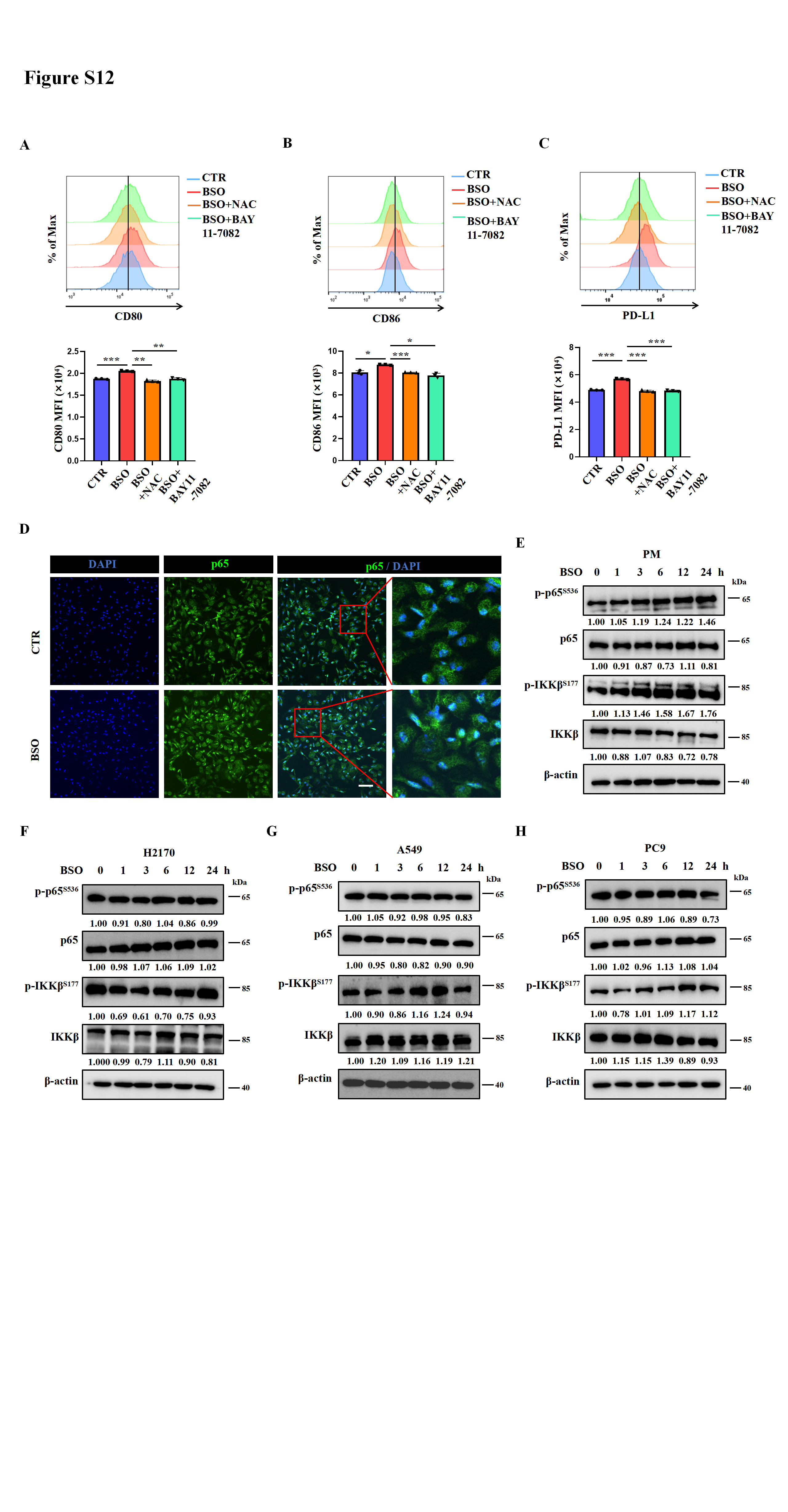

Supplement: Supplementary file 13 — Supporting Information [file ADVS-12-e13084-s012.tiff]

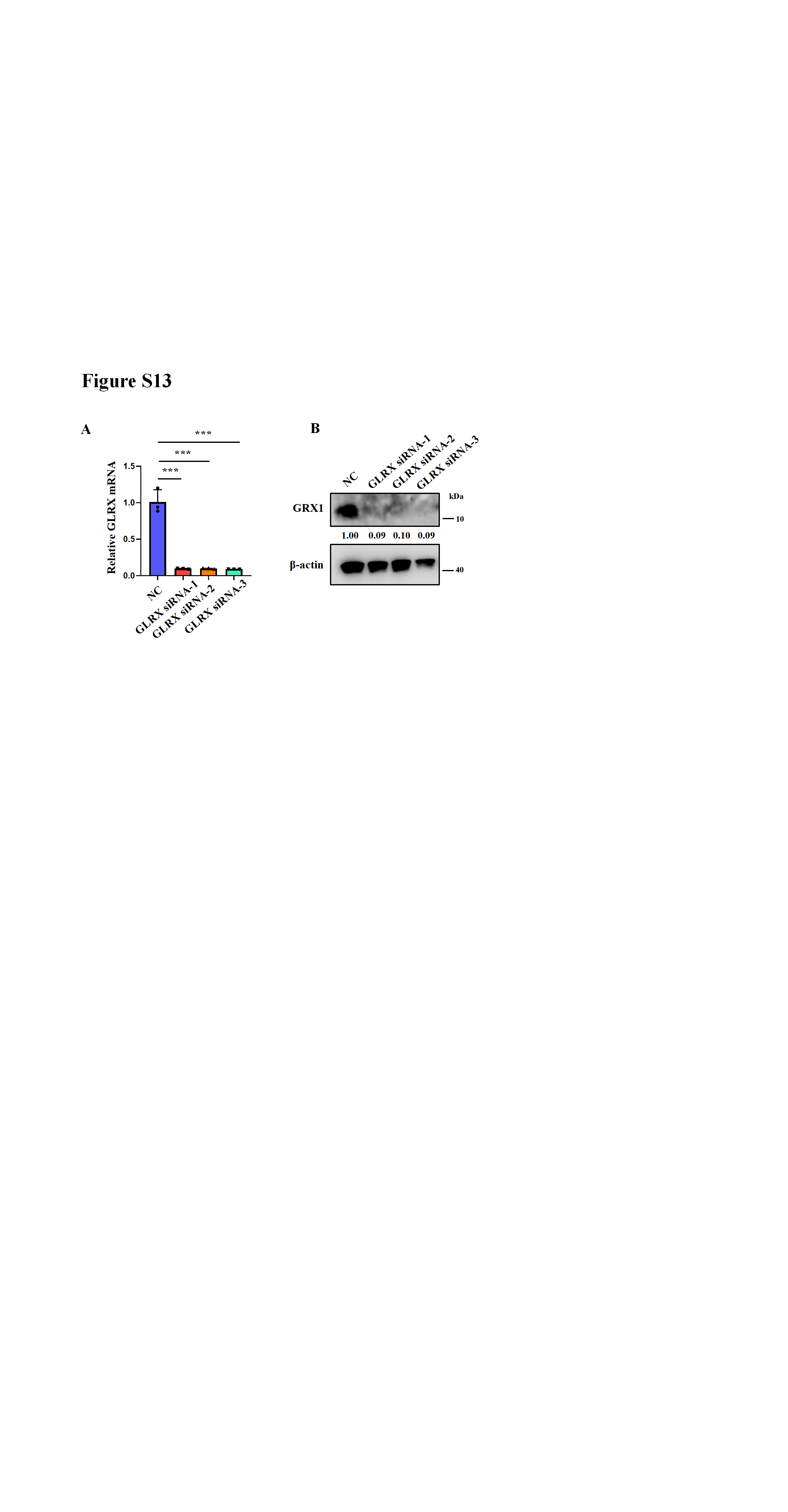

Supplement: Supplementary file 14 — Supporting Information [file ADVS-12-e13084-s001.tiff]
