# Supplementary material for: Targeting Cystine Metabolism in the Lung Cancer Environment Enhances the Efficacy of Immune Checkpoint Inhibition
Source: Adv Sci (Weinh). 2025 Jul 10;12(35):e13084. doi: 10.1002/advs.202413084 (PMC12463131; doi:10.1002/advs.202413084)
Supplement: Supplementary file 15 — Supporting Information [file ADVS-12-e13084-s014.docx]

**Table S1.** **Primers used for RT-PCR**

| **Target** | **Forward** | **Reverse** |
| --- | --- | --- |
| Mus-*Cxcl9* | TCCTTTTGGGCATCATCTTCC | TTTGTAGTGGATCGTGCCTCG |
| Mus-*Cxcl10* | CCAAGTGCTGCCGTCATTTTC | GGCTCGCAGGGATGATTTCAA |
| Mus-*Tnfa* | CCCTCACACTCAGATCATCTTCT | GCTACGACGTGGGCTACAG |
| Mus-*Il12a* | CTGTGCCTTGGTAGCATCTATG | GCAGAGTCTCGCCATTATGATTC |
| Mus-*Mrc1* | CTCTGTTCAGCTATTGGACGC | CGGAATTTCTGGGATTCAGCTTC |
| Mus-*CD274* | GCTCCAAAGGACTTGTACGTG | TGATCTGAAGGGCAGCATTTC |
| Mus-*Slc7a11* | GGCACCGTCATCGGATCAG | CTCCACAGGCAGACCAGAAAA |
| Mus-*Ifng* | ATGAACGCTACACACTGCATC | CCATCCTTTTGCCAGTTCCTC |
| Mus-*Prf1* | AGCACAAGTTCGTGCCAGG | GCGTCTCTCATTAGGGAGTTTTT |
| Mus-*Gzmb* | CCACTCTCGACCCTACATGG | GGCCCCCAAAGTGACATTTATT |
| Mus-*Actb* | GGCTGTATTCCCCTCCATCG | CCAGTTGGTAACAATGCCATGT |
| Homo-*Cxcl9* | GATTGGTGCCCAGTTAGCCT | CCACCGGACAGCACTCTAAA |
| Homo-*Cxcl10* | GTGGCATTCAAGGAGTACCTC | TGATGGCCTTCGATTCTGGATT |
| Homo-*Tnfa* | AGAACTCACTGGGGCCTACA | GCTCCGTGTCTCAAGGAAGT |
| Homo-*18S* | GCAATTATTCCCCATGAACG | GGGACTTAATCAACGCAAGC |
