# Supplementary material for: Targeting Cystine Metabolism in the Lung Cancer Environment Enhances the Efficacy of Immune Checkpoint Inhibition
Source: Adv Sci (Weinh). 2025 Jul 10;12(35):e13084. doi: 10.1002/advs.202413084 (PMC12463131; doi:10.1002/advs.202413084)
Supplement: Supplementary file 16 — Supporting Information [file ADVS-12-e13084-s013.docx]

**Table S2. Antibodies used for western blotting**

| **Antibody** | **Source** |
| --- | --- |
| Anti-p65 | CST |
| Anti-p-p65 | CST |
| Anti-IKKβ | CST |
| Anti-p-IKKβ | CST |
| Anti-PD-L1 | CST |
| Anti-PD-L1 | Proteintech |
| Anti-PD-L1 | Abclonal |
| Anti-GRX1 | Proteintech |
| Anti-Glutathione | VIROGEN |
| Anti-β-Actin | HuaBio |
| Goat anti-mouse IgG HRP | HuaBio |
| Goat anti-rabbit IgG HRP | HuaBio |
| Goat anti-mouse IgG iFluor594 | HuaBio |
| Goat anti-Rabbit IgG iFluor594 | HuaBio |
| Goat anti-mouse IgG | Invitrogen |
| Goat anti-rabbit IgG | Invitrogen |
| InVivoMAb anti-mouse PD-L1 | BioXcell |
| InVivoMAb rat IgG2b | BioXcell |
