# Supplementary material for: Targeting Cystine Metabolism in the Lung Cancer Environment Enhances the Efficacy of Immune Checkpoint Inhibition
Source: Adv Sci (Weinh). 2025 Jul 10;12(35):e13084. doi: 10.1002/advs.202413084 (PMC12463131; doi:10.1002/advs.202413084)
Supplement: Supplementary file 17 — Supporting Information [file ADVS-12-e13084-s005.docx]

**Table S3: Antibodies used for flow cytometry**

| **Antibody** | **Source** |
| --- | --- |
| Anti-mouse CD3 FITC | Biolegend |
| Anti-mouse CD4 APC-CY7 | Biolegend |
| Anti-mouse CD4 PE | eBioscience |
| Anti-human CD4 APC-CY7 | Biolegend |
| Anti-mouse CD8a APC | Biolegend |
| Anti-mouse CD8a BV650 | Biolegend |
| Anti-human CD8 BV650 | Biolegend |
| Anti-mouse/human CD11b APC | Biolegend |
| Anti-mouse CD11b PE | eBioscience |
| Anti-mouse CD11b PE-Cy7 | eBioscience |
| Anti-mouse CD11c APC | Biolegend |
| Anti-mouse CD11c BV605 | Biolegend |
| Anti-human CD11c BV605 | Biolegend |
| Anti-human CD14 APC | Biolegend |
| Anti-human CD16 BV421 | Biolegend |
| Anti-mouse CD19 BV605 | Biolegend |
| Anti-human CD19 BV650 | Biolegend |
| Anti-mouse CD31 PE | Biolegend |
| Anti-human CD31 BV605 | Biolegend |
| Anti-mouse CD45 APC-Cy7 | Biolegend |
| Anti-mouse CD45 PE | Biolegend |
| Anti-mouse CD45 APC | Biolegend |
| Anti-human CD45 PE | Biolegend |
| Anti-human CD68 APC | Biolegend |
| Anti-mouse CD86 PE | eBioscience |
| Anti-mouse CD140a BV421 | Biolegend |
| Anti-mouse CD206 APC | eBioscience |
| Anti-mouse CD206 eFluor450 | eBioscience |
| Anti-human CD335 PE-CY7 | Biolegend |
| Anti-mouse F4/80 FITC | Biolegend |
| Anti-mouse F4/80 APC-Cy7 | Biolegend |
| Anti-mouse F4/80 PE | Biolegend |
| Anti-mouse Ly6G BV421 | Biolegend |
| Anti-mouse Ly6G BV650 | Biolegend |
| Anti-mouse Ly6C APC | Biolegend |
| Anti-mouse MHC II PE | Biolegend |
| Anti-mouse NK1.1 PE | Biolegend |
| Anti-mouse NK1.1 PE-CY7 | Biolegend |
| Anti-mouse PD-L1 APC | Biolegend |
| Anti-human PD-L1 APC | Biolegend |
| Anti-human/mouse XCT FITC | NOVUSBIO |
| Fixable Viability Dye eFluor450 | eBioscience |
| Fixable Viability Dye | eBioscience |
| Zombie | Biolegend |
