# Supplementary material for: Targeting Cystine Metabolism in the Lung Cancer Environment Enhances the Efficacy of Immune Checkpoint Inhibition
Source: Adv Sci (Weinh). 2025 Jul 10;12(35):e13084. doi: 10.1002/advs.202413084 (PMC12463131; doi:10.1002/advs.202413084)
Supplement: Supplementary file 18 — Supporting Information [file ADVS-12-e13084-s010.docx]

**Table S4. Sequences of siRNA**

| **Gene** | **Sequence** |
| --- | --- |
| NC siRNA F | UUCUCCGAACGUGUCACGUdTdT |
| NC siRNA R | ACGUGACACGUUCGGAGAAdTdT |
| Mus-*Glrx1* siRNA-1 F | CAGUGCGAUUCAAGAUUAUTT |
| Mus-*Glrx1* siRNA-1 R | AUAAUCUUGAAUCGCACUGTT |
| Mus-*Glrx1* siRNA-2 F | GACCCAAGAAAUCCUCAGUTT |
| Mus-*Glrx1* siRNA-2 R | ACUGAGGAUUUCUUGGGUCTT |
| Mus-*Glrx1* siRNA-3 F | GCAGUGAUCUAAUCUCCAUTT |
| Mus-*Glrx1* siRNA-3 R | AUGGAGAUUAGAUCACUGCTT |
