# Supplementary material for: Targeting Cystine Metabolism in the Lung Cancer Environment Enhances the Efficacy of Immune Checkpoint Inhibition
Source: Adv Sci (Weinh). 2025 Jul 10;12(35):e13084. doi: 10.1002/advs.202413084 (PMC12463131; doi:10.1002/advs.202413084)
Supplement: Supplementary file 19 — Supporting Information [file ADVS-12-e13084-s006.ppt]

## Slide 1
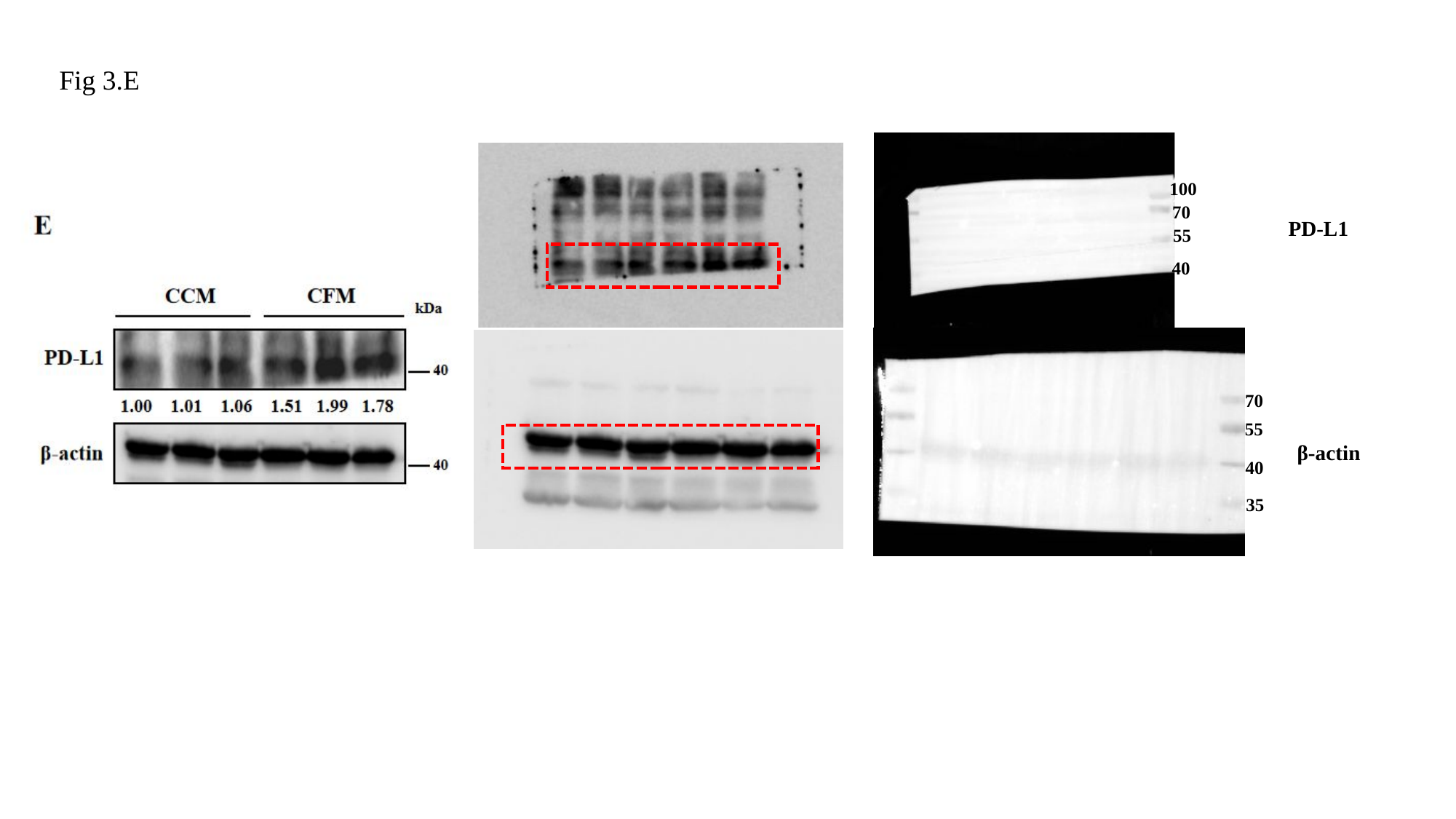

Fig 3.E
100
70
PD-L1
55
40
70
55
β-actin
40
35

## Slide 2
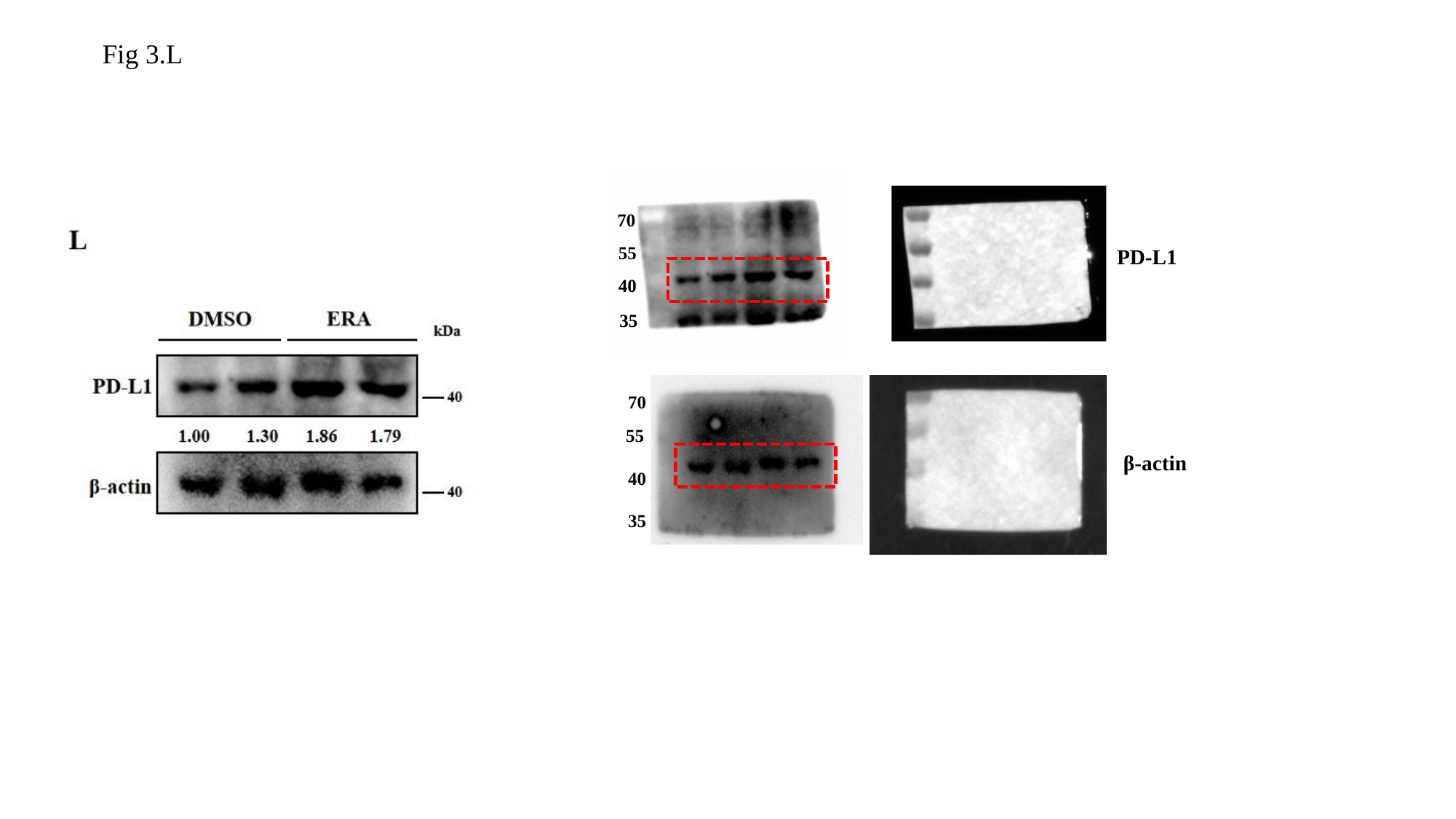

Fig 3.L
70
55
PD-L1
40
35
70
55
β-actin
40
35

## Slide 3
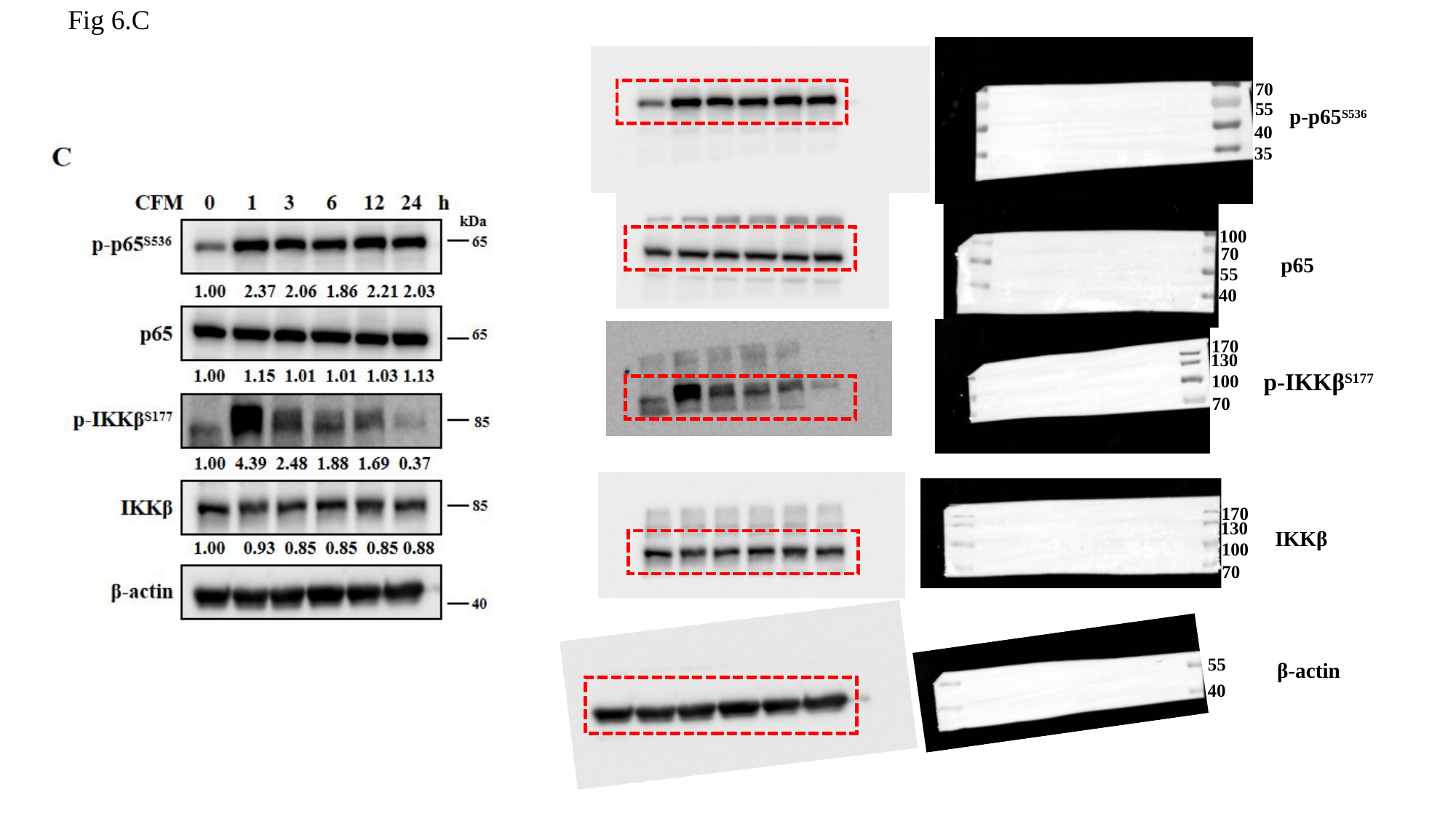

Fig 6.C
70
55
p-p65S536
40
35
100
70
p65
55
40
170
130
p-IKKβS177
100
70
170
130
IKKβ
100
70
55
β-actin
40

## Slide 4
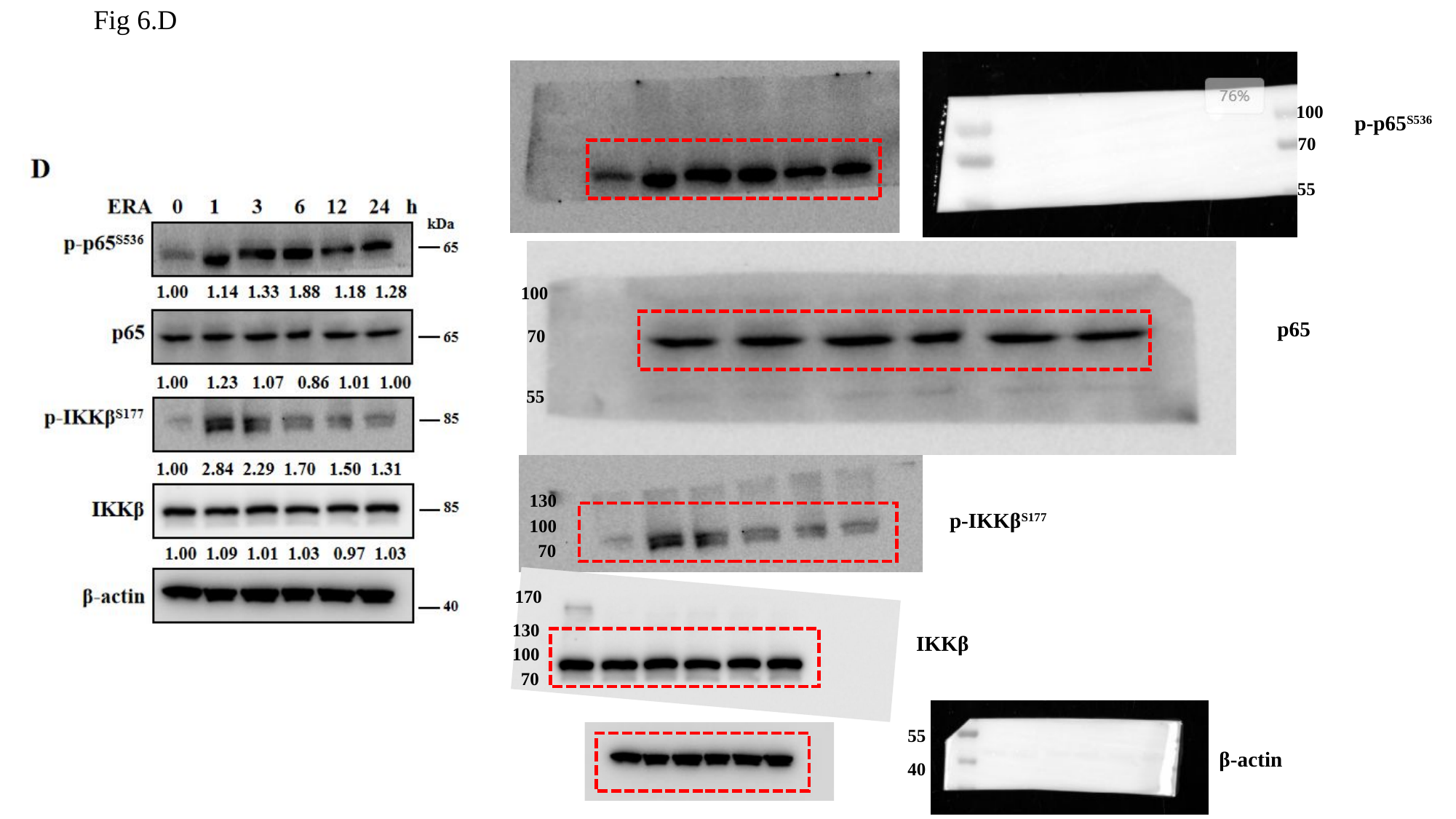

Fig 6.D
100
p-p65S536
70
55
100
p65
70
55
130
p-IKKβS177
100
70
170
130
IKKβ
100
70
55
β-actin
40

## Slide 5
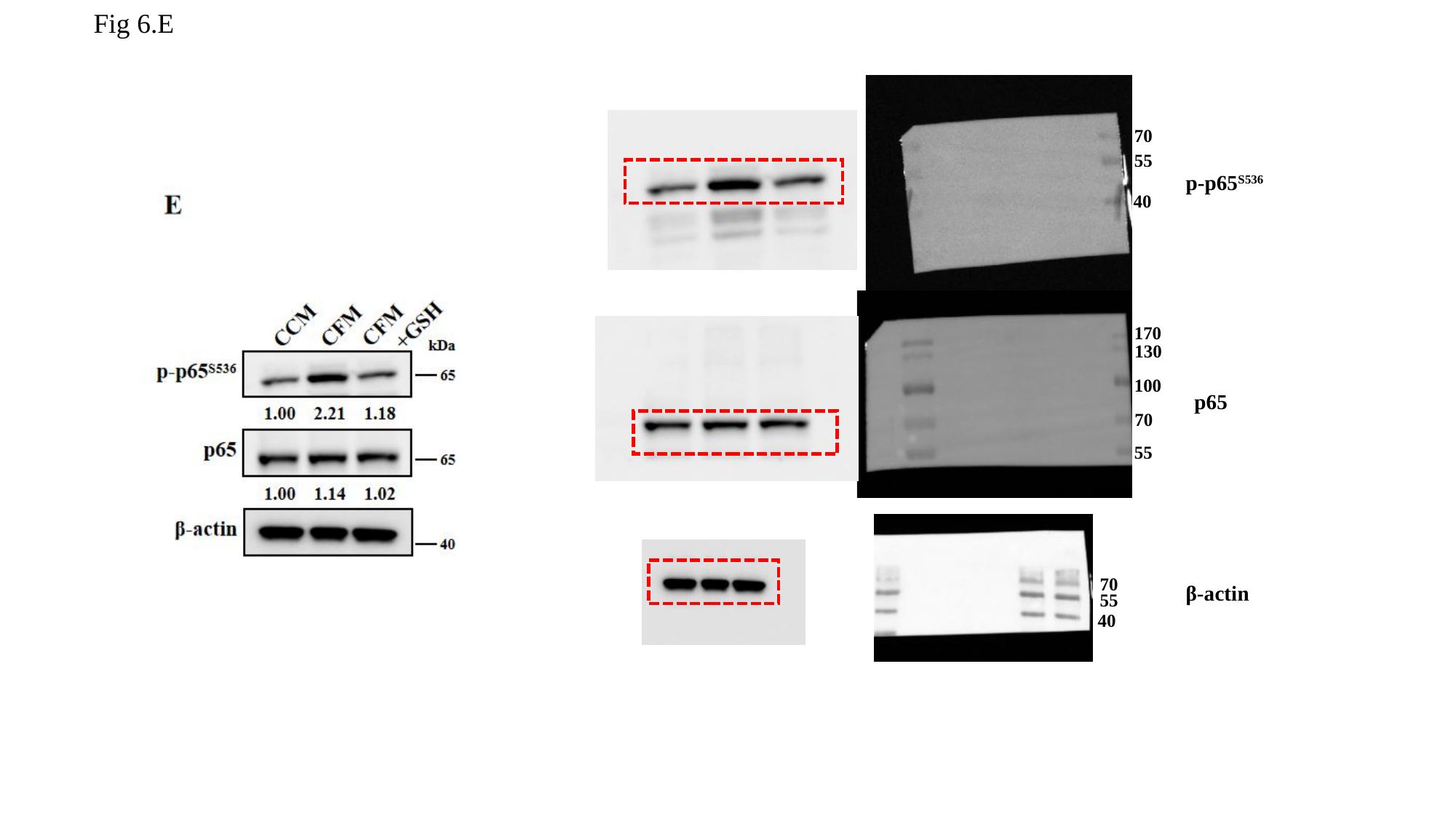

Fig 6.E
70
55
p-p65S536
40
170
130
100
p65
70
55
70
β-actin
55
40

## Slide 6
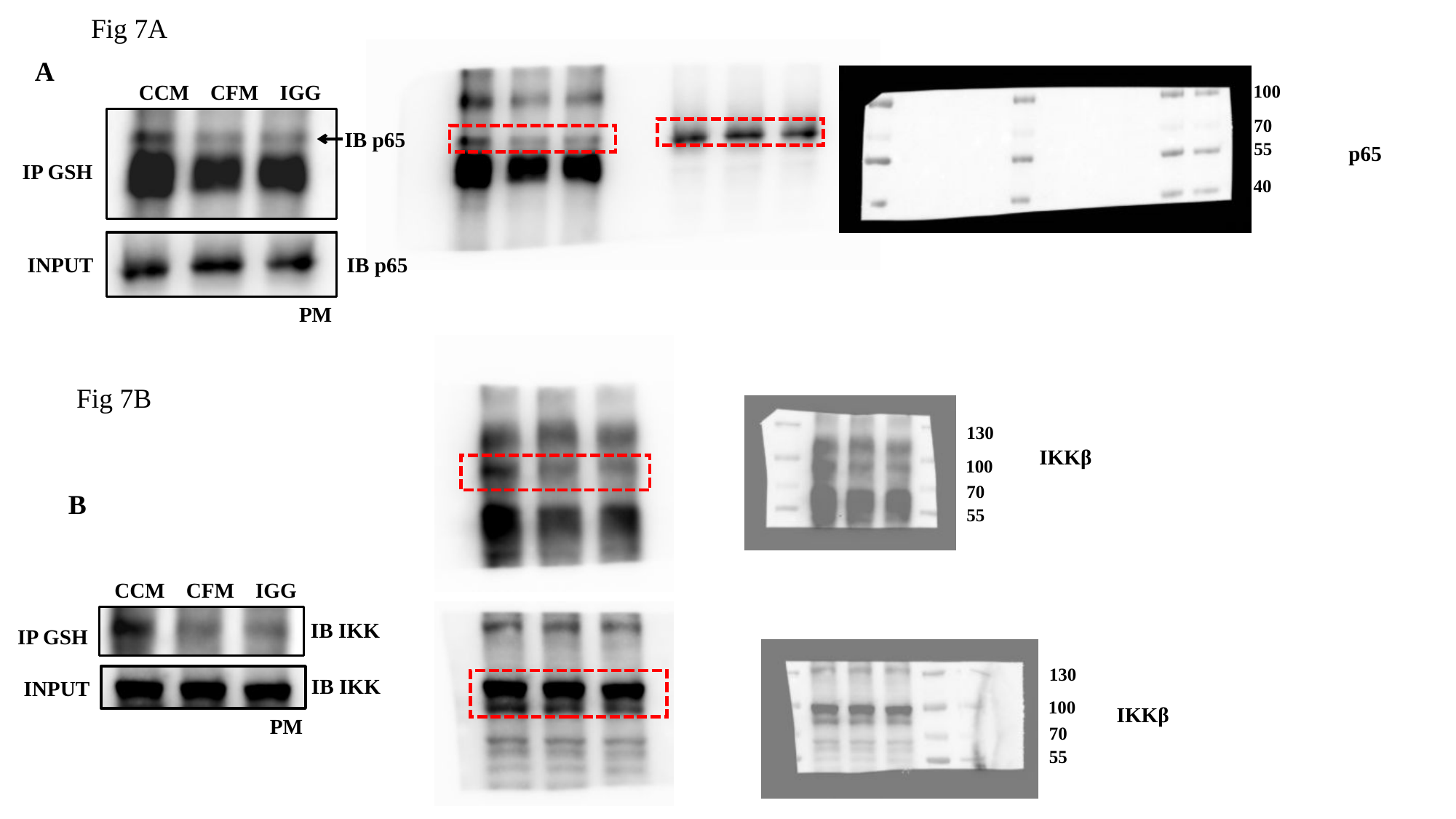

Fig 7A
A
CCM CFM IGG
IB p65
IP GSH
INPUT
IB p65
PM
100
70
55
p65
40
Fig 7B
130
IKKβ
100
70
B
CCM CFM IGG
IB IKK
IP GSH
IB IKK
INPUT
PM
55
130
100
IKKβ
70
55

## Slide 7
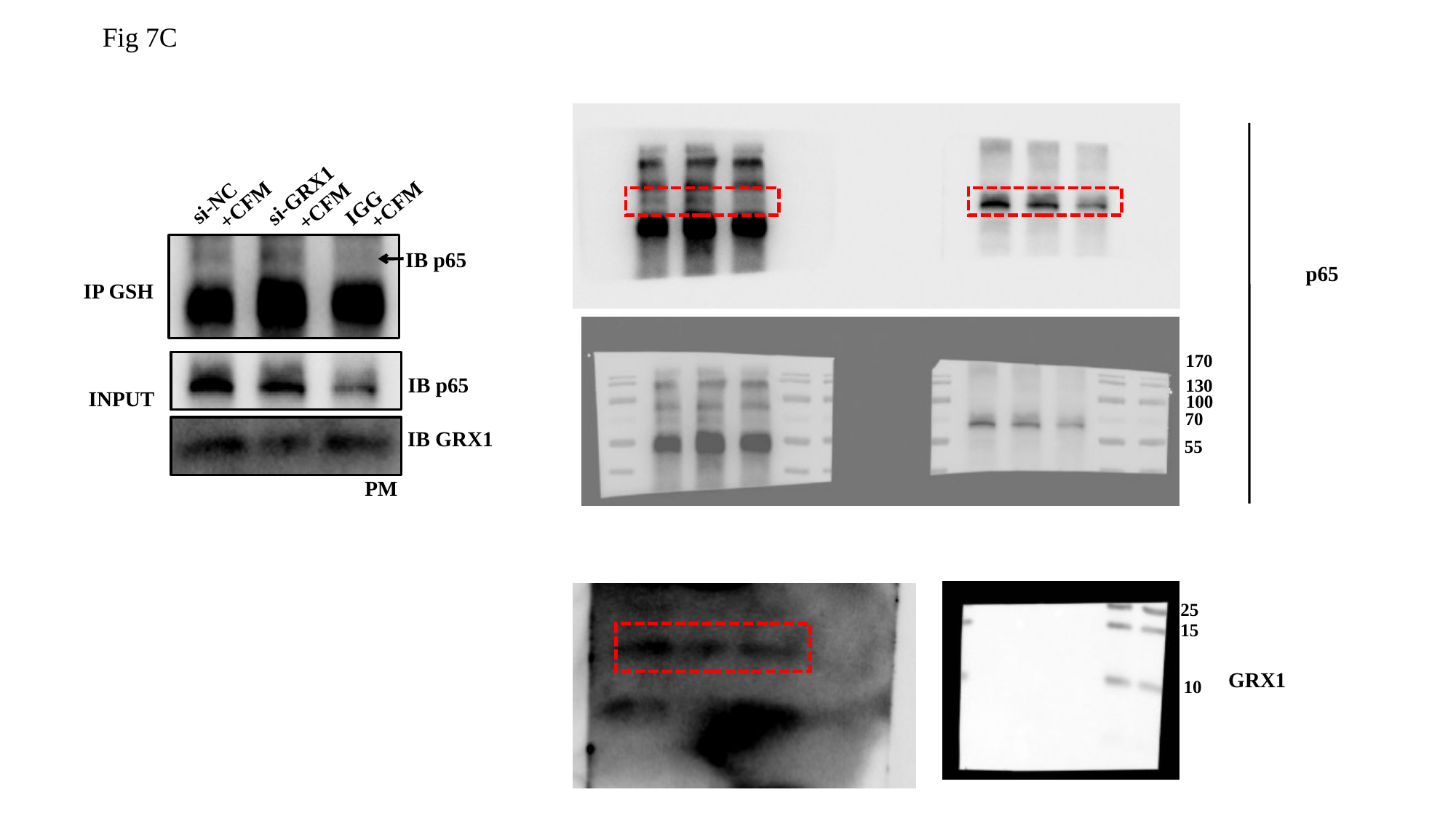

Fig 7C
si-NC
si-GRX1
IGG
IB p65
IP GSH
IB p65
INPUT
PM
IB GRX1
+CFM
+CFM
+CFM
p65
170
130
100
70
55
25
15
GRX1
10

## Slide 8
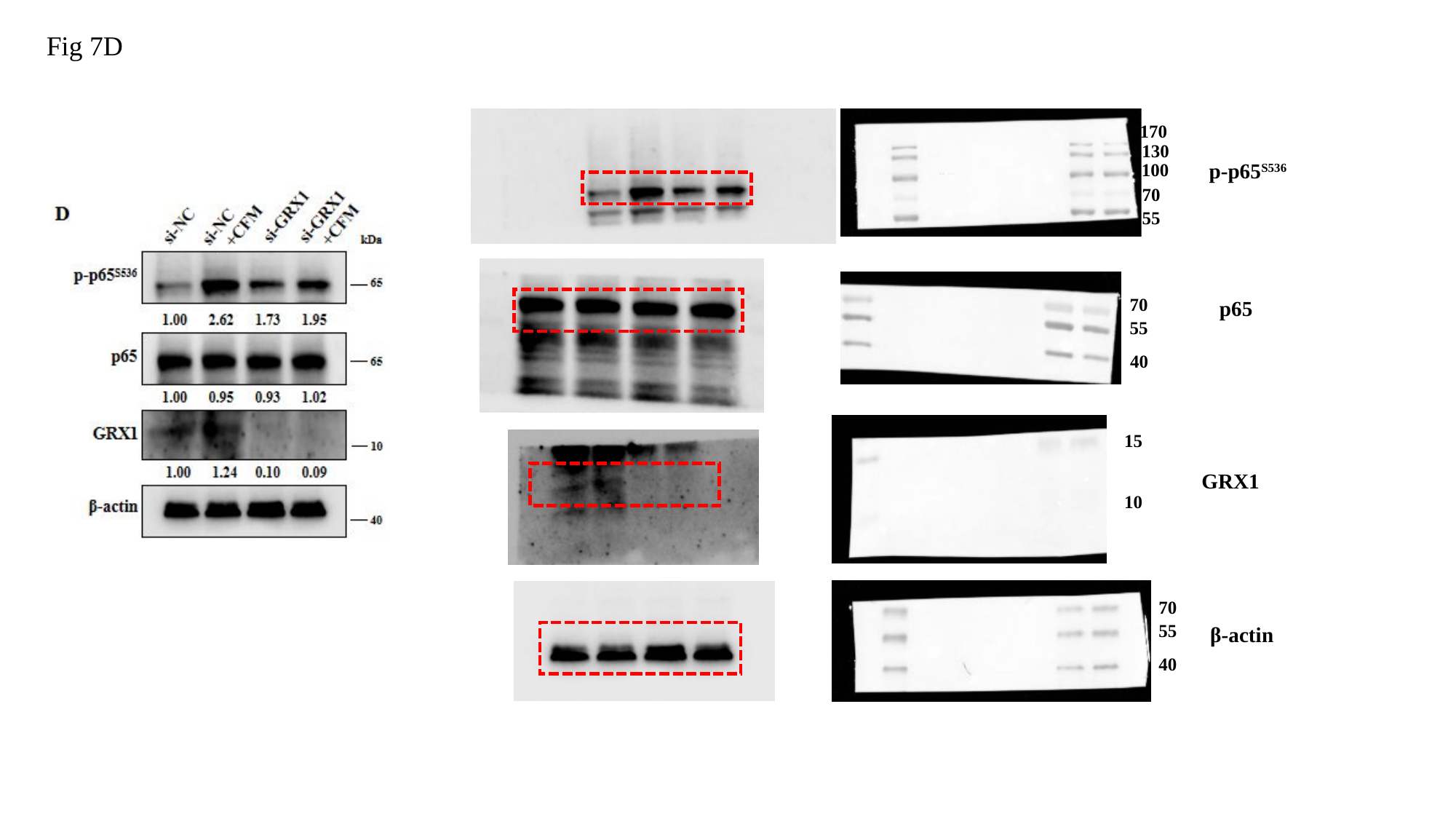

Fig 7D
170
130
p-p65S536
100
70
55
70
p65
55
40
15
GRX1
10
70
55
β-actin
40

## Slide 9
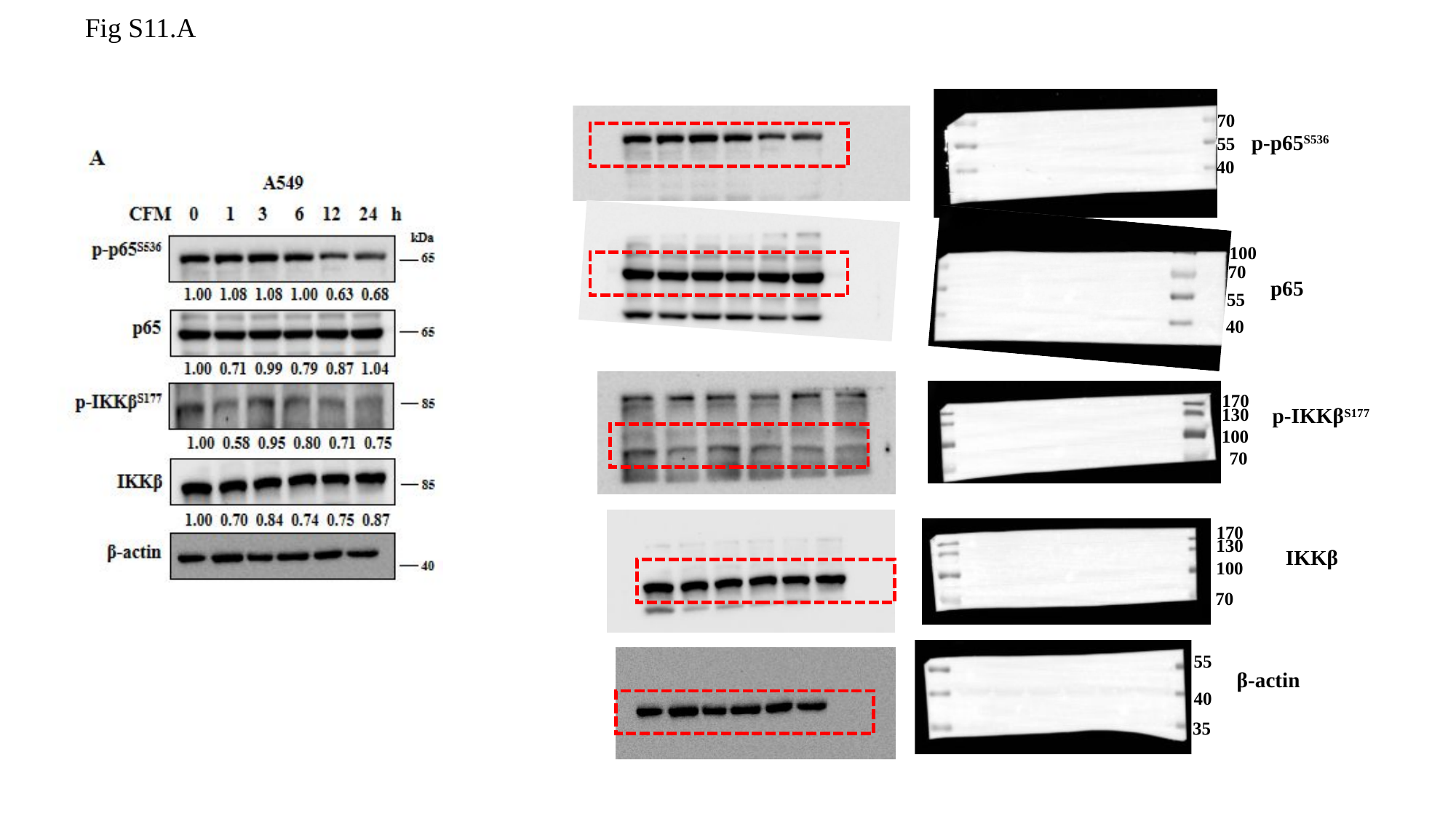

Fig S11.A
70
p-p65S536
55
40
100
70
p65
55
40
170
p-IKKβS177
130
100
70
170
130
IKKβ
100
70
55
β-actin
40
35

## Slide 10
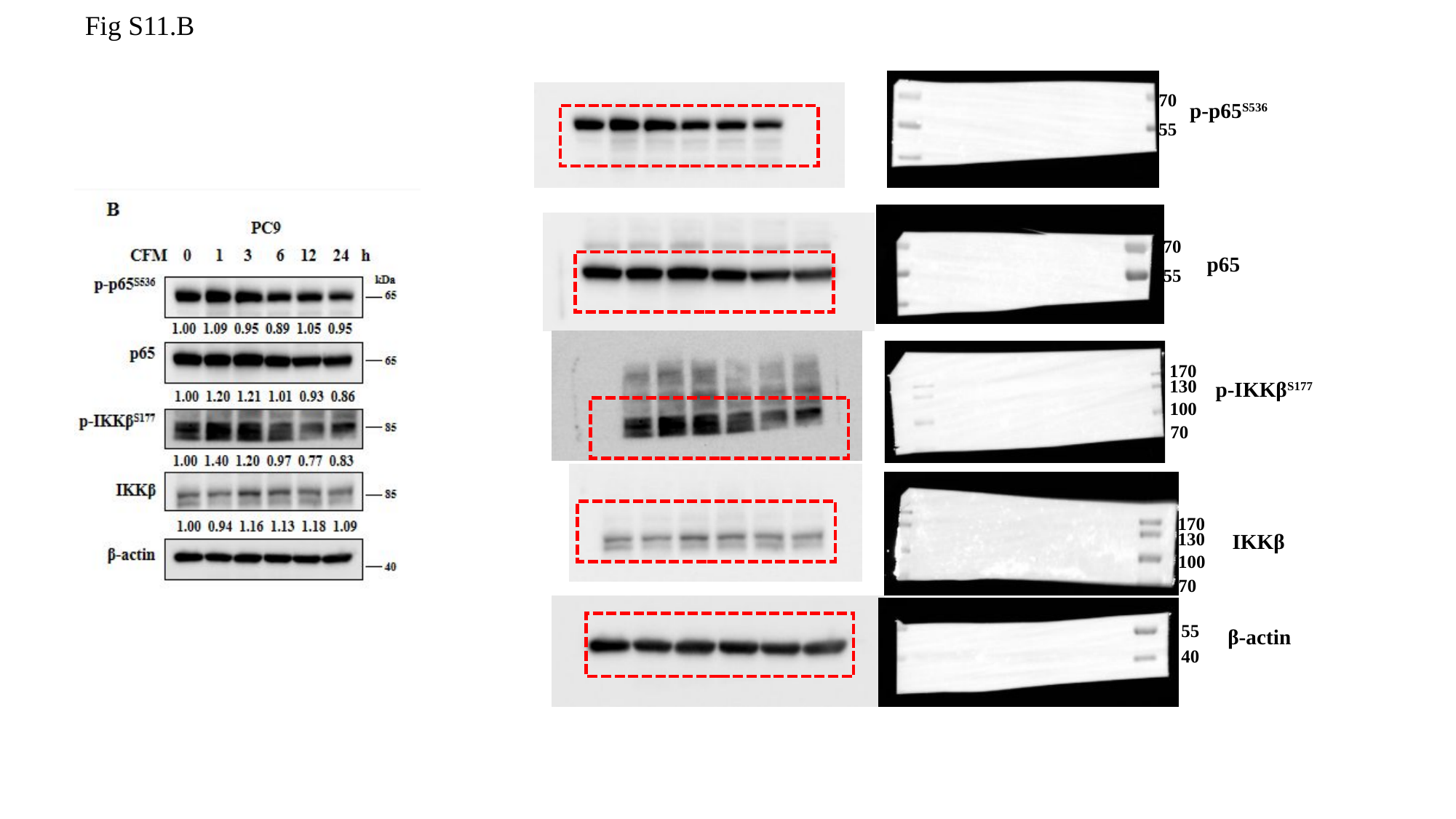

Fig S11.B
70
p-p65S536
55
70
p65
55
170
130
p-IKKβS177
100
70
170
130
IKKβ
100
70
55
β-actin
40

## Slide 11
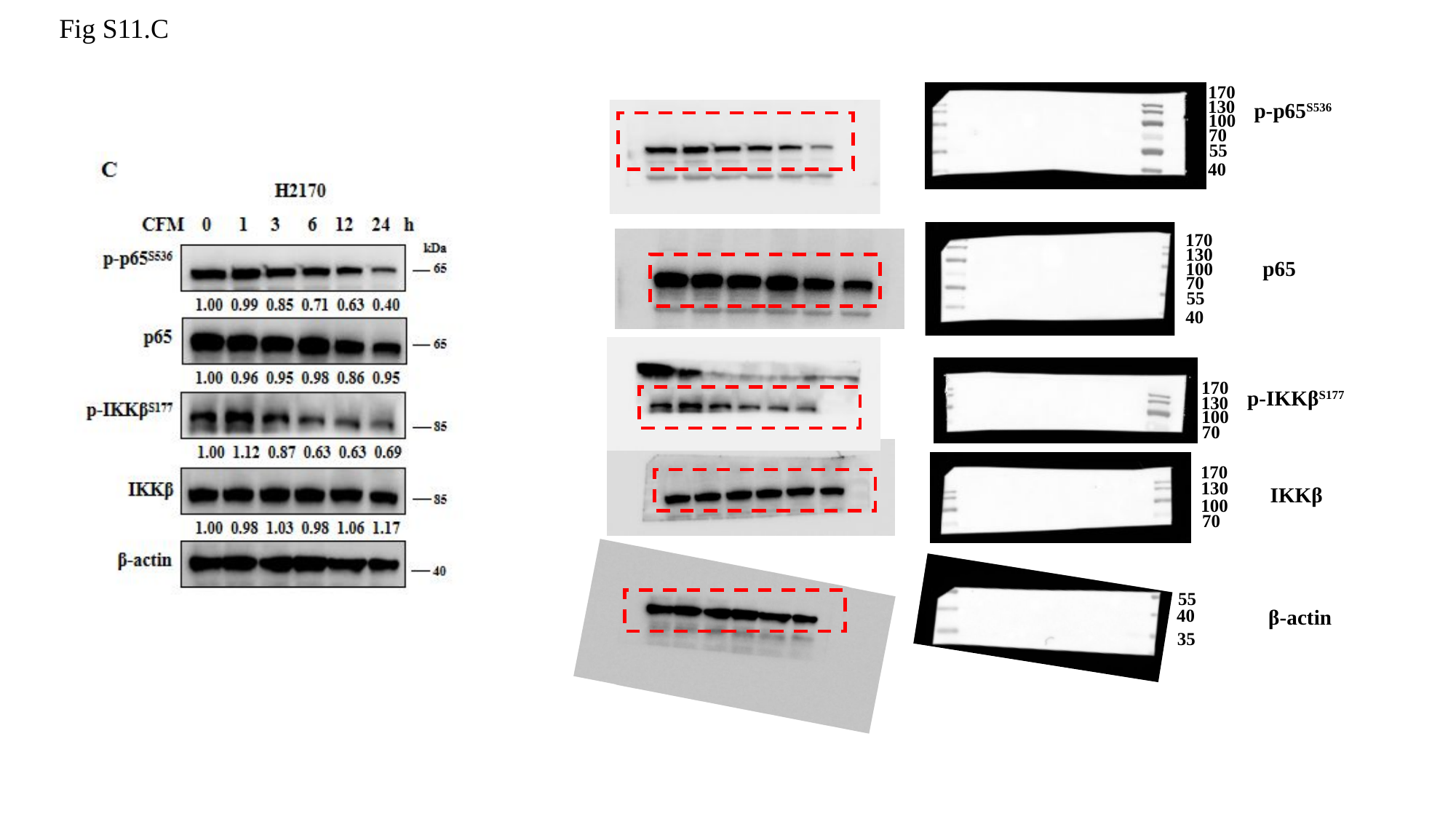

Fig S11.C
170
130
p-p65S536
100
70
55
40
170
130
p65
100
70
55
40
170
p-IKKβS177
130
100
70
170
130
IKKβ
100
70
55
40
β-actin
35

## Slide 12
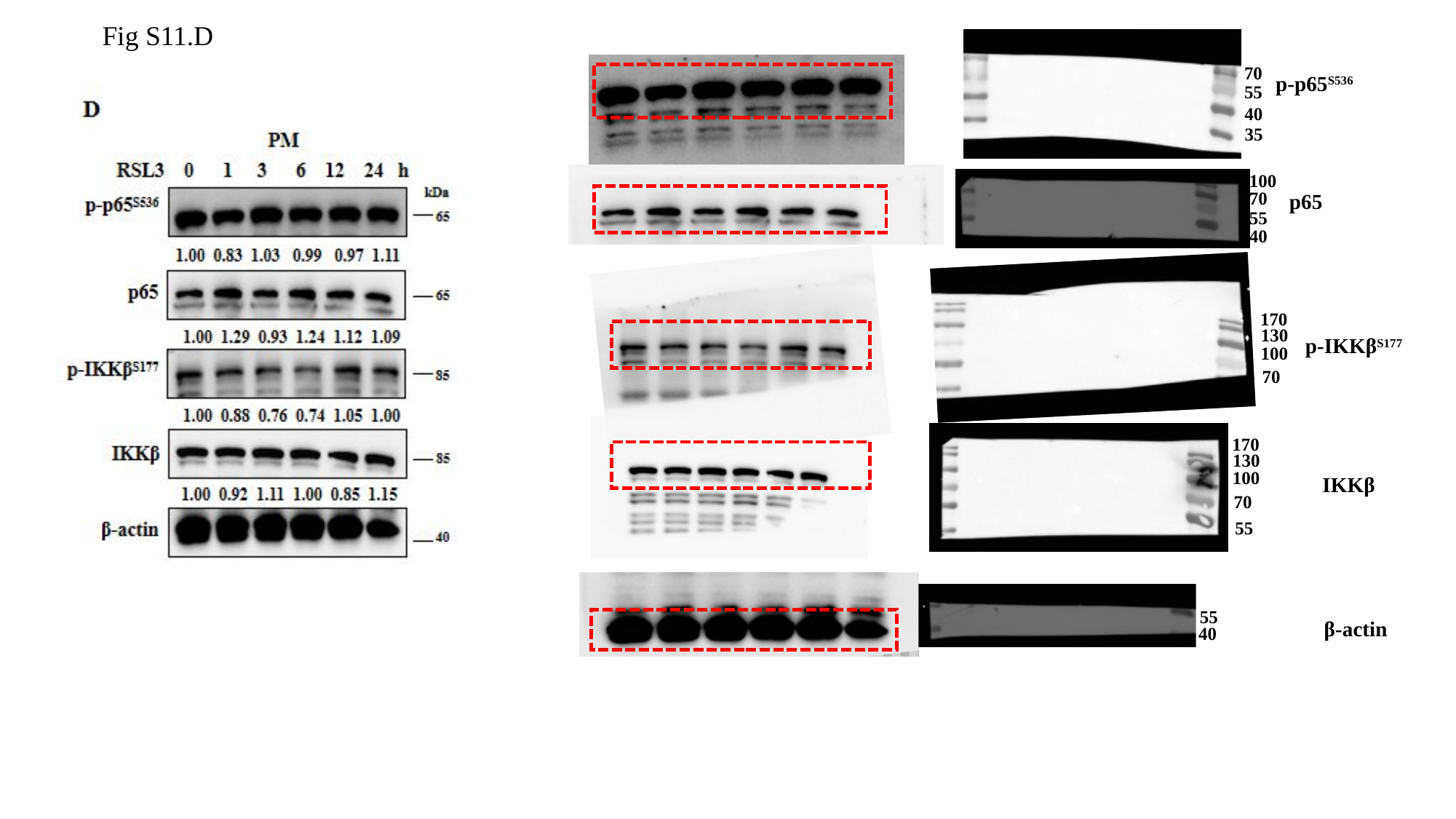

Fig S11.D
70
p-p65S536
55
40
35
100
70
p65
55
40
170
130
p-IKKβS177
100
70
170
130
100
IKKβ
70
55
55
β-actin
40

## Slide 13
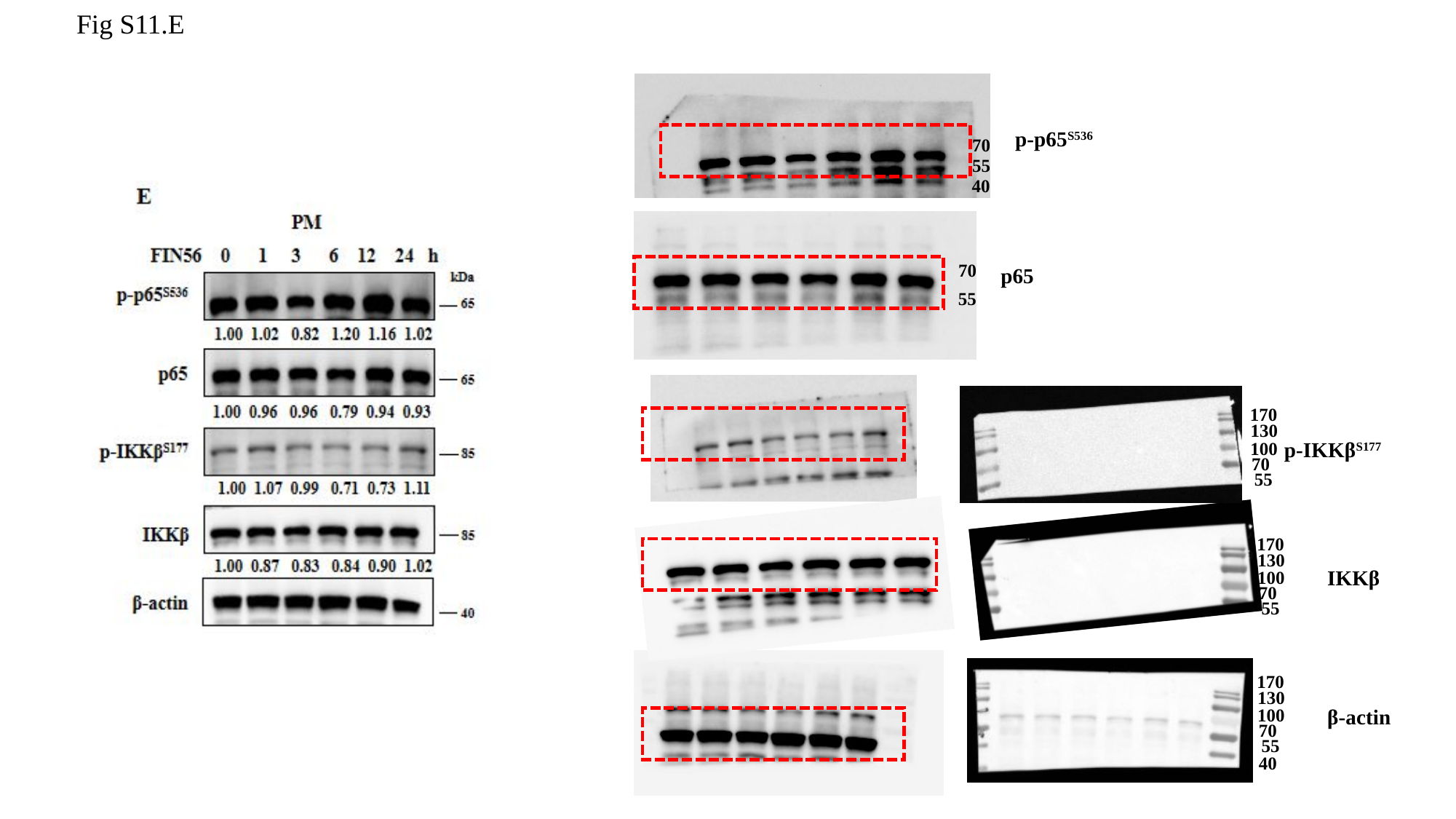

Fig S11.E
p-p65S536
70
55
40
70
p65
55
170
130
p-IKKβS177
100
70
55
170
130
IKKβ
100
70
55
170
130
β-actin
100
70
55
40

## Slide 14
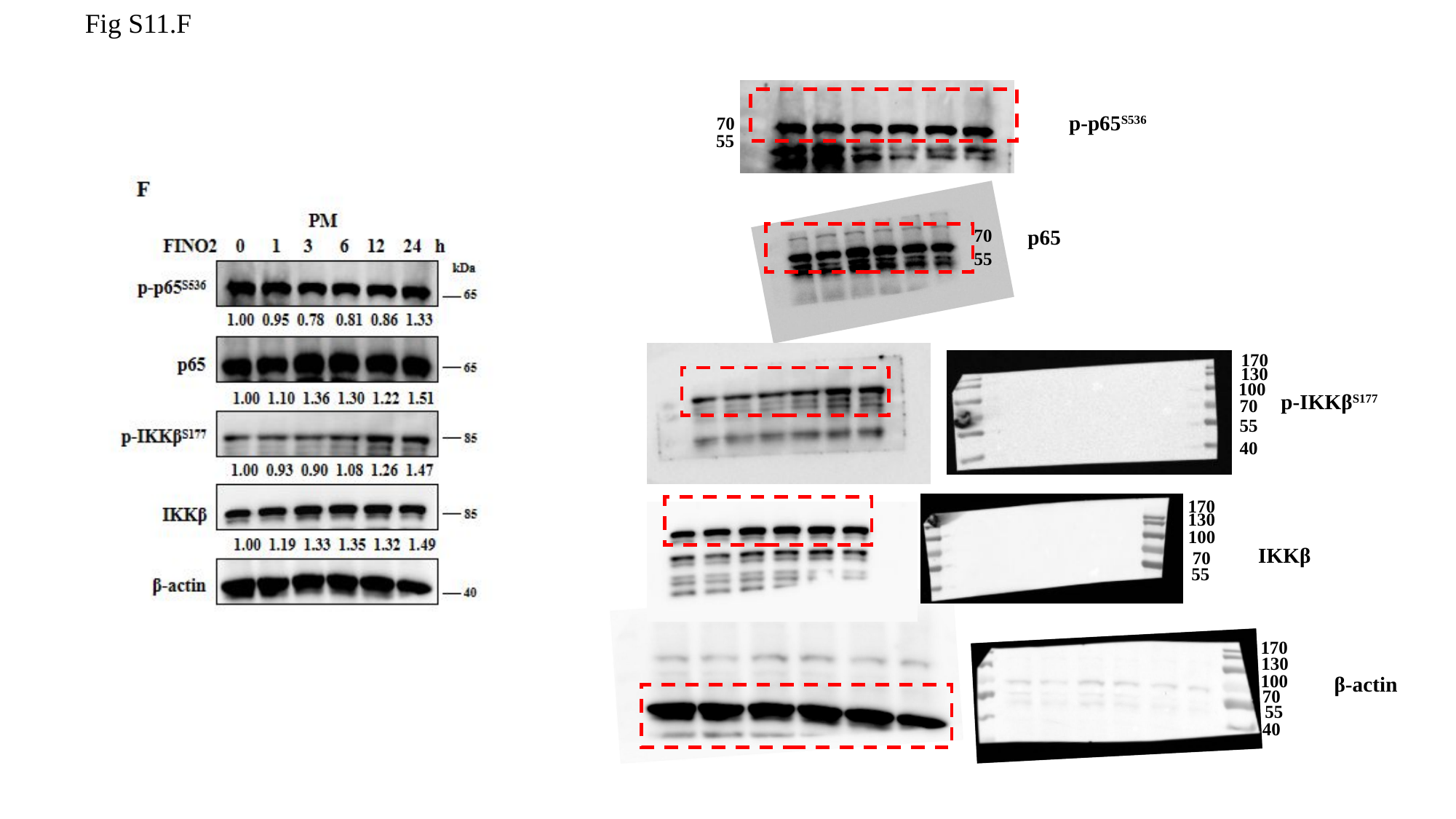

Fig S11.F
p-p65S536
70
55
70
p65
55
170
130
100
p-IKKβS177
70
55
40
170
130
100
IKKβ
70
55
170
130
100
β-actin
70
55
40

## Slide 15
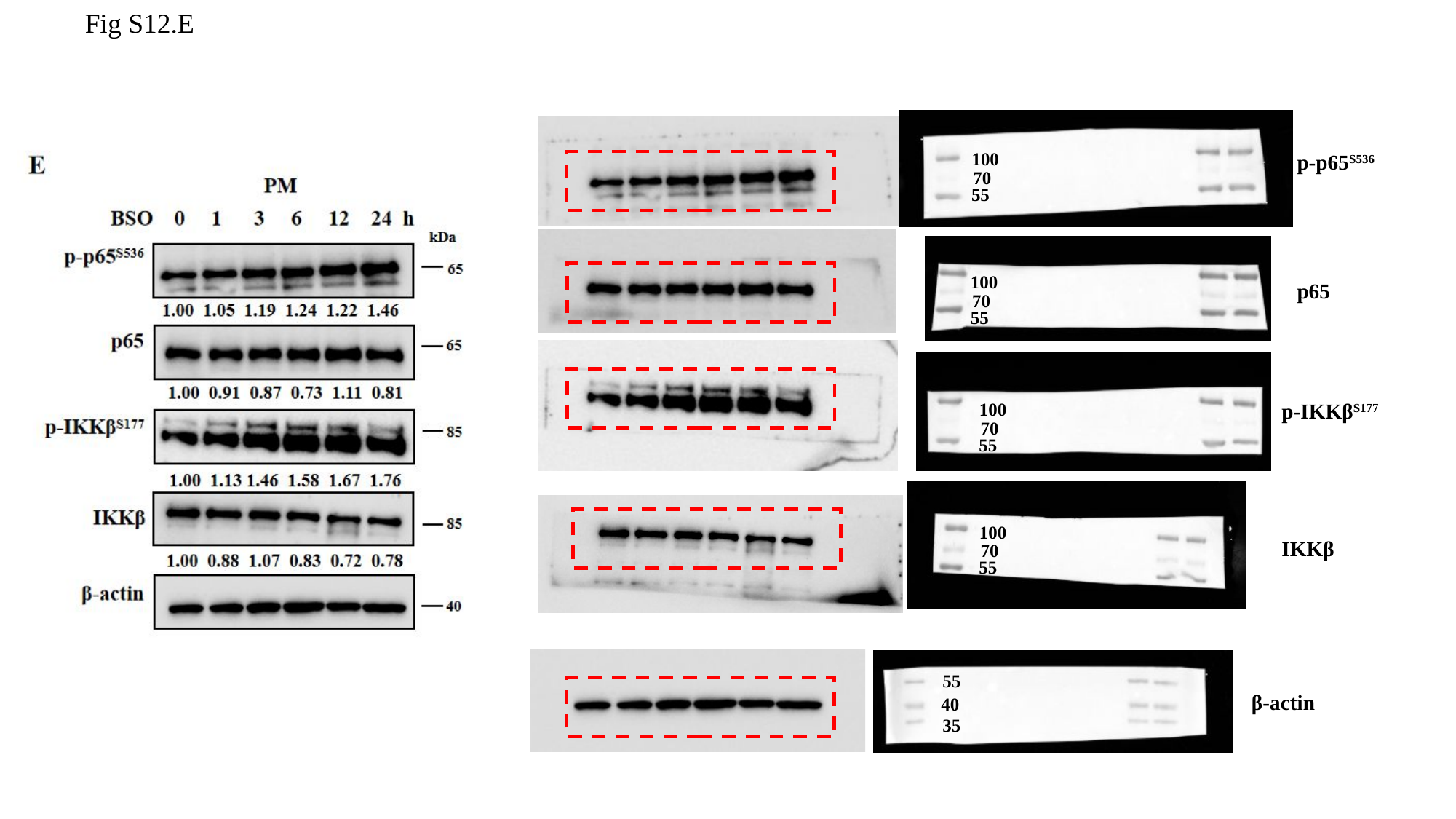

Fig S12.E
100
p-p65S536
70
55
100
p65
70
55
100
p-IKKβS177
70
55
100
IKKβ
70
55
55
β-actin
40
35

## Slide 16
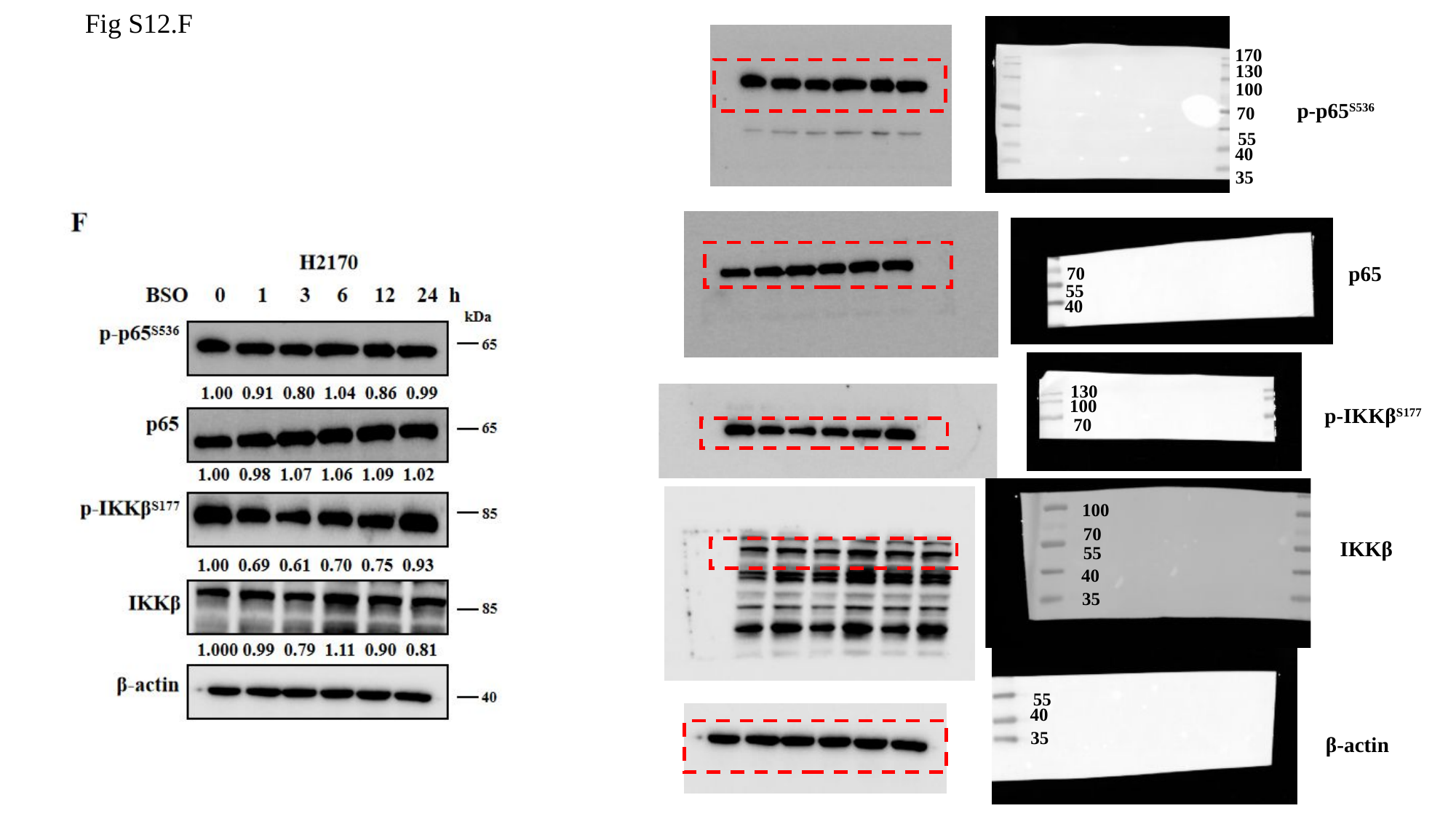

Fig S12.F
170
130
100
p-p65S536
70
55
40
35
p65
70
55
40
130
100
p-IKKβS177
70
100
70
IKKβ
55
40
35
55
40
35
β-actin

## Slide 17
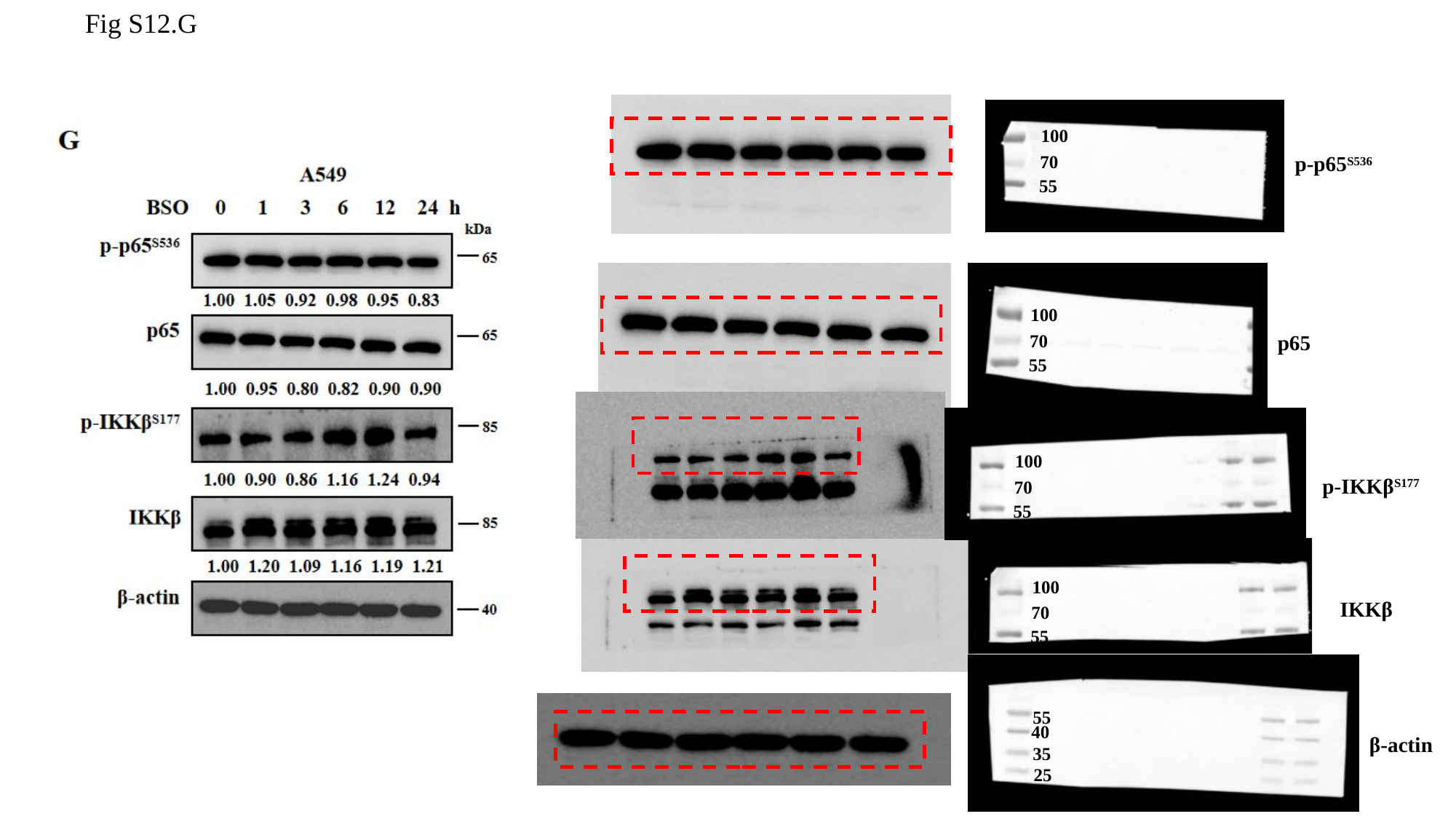

Fig S12.G
100
70
p-p65S536
55
100
p65
70
55
100
p-IKKβS177
70
55
100
IKKβ
70
55
55
40
β-actin
35
25

## Slide 18
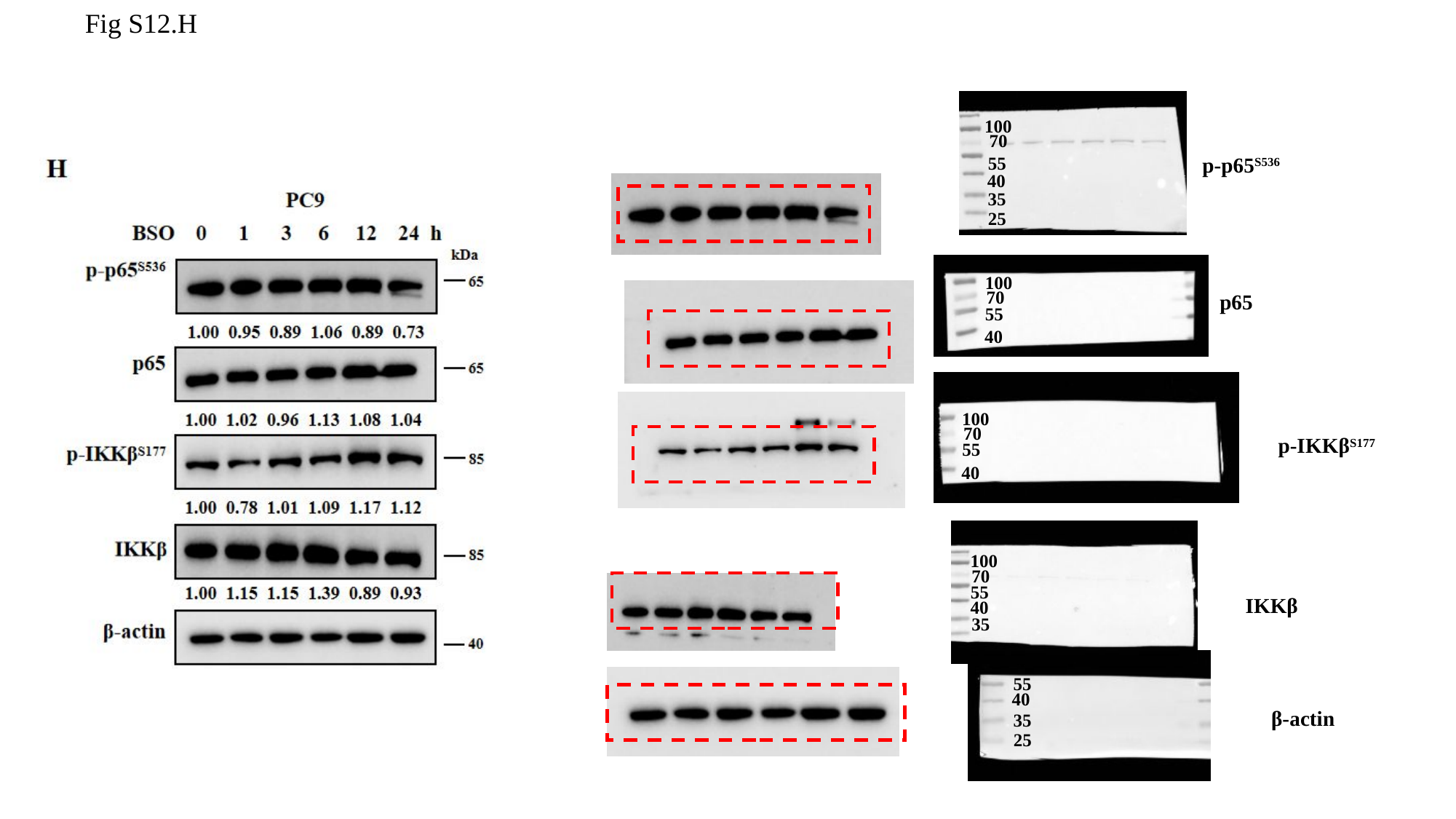

Fig S12.H
100
70
55
p-p65S536
40
35
25
100
70
p65
55
40
100
70
p-IKKβS177
55
40
100
70
55
IKKβ
40
25
35
55
40
β-actin
35
25

## Slide 19
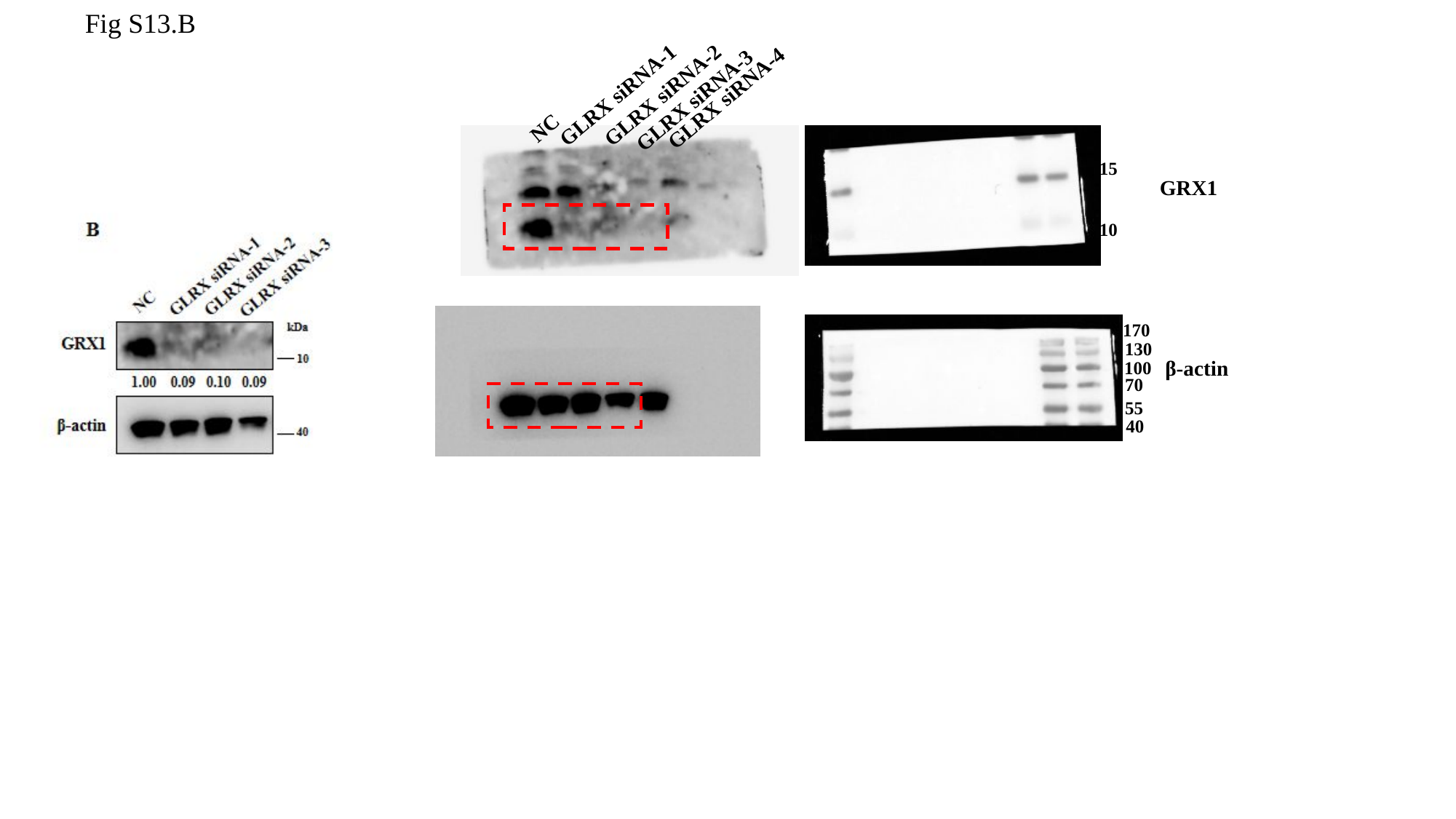

Fig S13.B
GLRX siRNA-2
GLRX siRNA-4
 GLRX siRNA-1
GLRX siRNA-3
NC
15
GRX1
10
170
130
β-actin
100
70
55
40
